# miR-128a Acts as a Regulator in Cardiac Development by Modulating Differentiation of Cardiac Progenitor Cell Populations

**DOI:** 10.3390/ijms21031158

**Published:** 2020-02-10

**Authors:** Sarah C. Hoelscher, Theresia Stich, Anne Diehm, Harald Lahm, Martina Dreßen, Zhong Zhang, Irina Neb, Zouhair Aherrahrou, Jeanette Erdmann, Heribert Schunkert, Gianluca Santamaria, Giovanni Cuda, Ralf Gilsbach, Lutz Hein, Rüdiger Lange, David Hassel, Markus Krane, Stefanie A. Doppler

**Affiliations:** 1 Department of Cardiovascular Surgery, German Heart Center Munich at the Technische Universität München, 80636 Munich, Germany; sarah.c.hoelscher@gmail.com (S.C.H.); theresia.stich@t-online.de (T.S.); anne.kistner@gmx.de (A.D.); lahm@dhm.mhn.de (H.L.); dressen@dhm.mhn.de (M.D.); dor.zhangzhong@gmail.com (Z.Z.); neb@dhm.mhn.de (I.N.); lange@dhm.mhn.de (R.L.); 2 Institute for Translational Cardiac Surgery (INSURE), Cardiovascular Surgery, 80636 Munich, Germany; 3 German Center for Cardiovascular Research (DZHK)—Partner Site Munich Heart Alliance, Biedersteiner Strasse 29, 80802 München, Germany; schunkert@dhm.mhn.de; 4 Institute for Cardiogenetics, University of Lübeck, 23562 Lübeck, Germany; zouhair.aherrahrou@uni-luebeck.de (Z.A.); jeanette.erdmann@uni-luebeck.de (J.E.); 5 Heart Center Lübeck, University of Lübeck, 23562 Lübeck, Germany; 6 German Center for Cardiovascular Research (DZHK)—Partner Site Hamburg/Lübeck/Kiel, 20246 Hamburg, Germany; 7 Department of Cardiology, German Heart Center Munich at the Technische Universität München, 80636 Munich, Germany; 8 Medical Department 1, Cardiology, Klinikum rechts der Isar, Technische Universität, 81675 Munich, Germany; santamariagianluca@gmail.com; 9 Department of Experimental and Clinical Medicine, Stem Cell Laboratory, Research Center of Advanced Biochemistry and Molecular Biology, University “Magna Graecia” of Catanzaro, Viale Europa, 88100 Catanzaro, Italy; cuda@unicz.it; 10 Institute of Experimental and Clinical Pharmacology and Toxicology, Faculty of Medicine, University of Freiburg, Albertstrasse 25, 79104 Freiburg, Germanylutz.hein@pharmakol.uni-freiburg.de (L.H.); 11 Institute for Cardiovascular Physiology, Goethe University, Theodor-Stern-Kai 7, 60596 Frankfurt am Main, Germany; 12 German Center for Cardiovascular Research (DZHK)—Partner Site RheinMain, 61231 Bad Nauheim, Germany; 13 BIOSS Centre for Biological Signaling Studies, University of Freiburg, Schänzlestrasse 1, 79104 Freiburg, Germany; 14 Department of Medicine III, Cardiology, Angiology, Pneumology, University Hospital Heidelberg, 69120 Heidelberg, Germany; d.hassel@me.com; 15 German Center for Cardiovascular Research (DZHK)—Partner Site Heidelberg/Mannheim, 69120 Heidelberg, Germany

**Keywords:** microRNA, miR-128, cardiac development, cardiac progenitor cells, *Nkx2.5* cardiac enhancer

## Abstract

MicroRNAs (miRs) appear to be major, yet poorly understood players in regulatory networks guiding cardiogenesis. We sought to identify miRs with unknown functions during cardiogenesis analyzing the miR-profile of multipotent *Nkx2.5* enhancer cardiac progenitor cells (NkxCE-CPCs). Besides well-known candidates such as miR-1, we found about 40 miRs that were highly enriched in NkxCE-CPCs, four of which were chosen for further analysis. Knockdown in zebrafish revealed that only miR-128a affected cardiac development and function robustly. For a detailed analysis, loss-of-function and gain-of-function experiments were performed during in vitro differentiations of transgenic murine pluripotent stem cells. MiR-128a knockdown (1) increased *Isl1*, *Sfrp5*, and *Hcn4* (cardiac transcription factors) but reduced *Irx4* at the onset of cardiogenesis, (2) upregulated *Isl1*-positive CPCs, whereas NkxCE-positive CPCs were downregulated, and (3) increased the expression of the ventricular cardiomyocyte marker *Myl2* accompanied by a reduced beating frequency of early cardiomyocytes. Overexpression of miR-128a (4) diminished the expression of *Isl1*, *Sfrp5*, *Nkx2.5*, and *Mef2c*, but increased *Irx4*, (5) enhanced NkxCE-positive CPCs, and (6) favored nodal-like cardiomyocytes (*Tnnt2*^+^, *Myh6*^+^, *Shox2*^+^) accompanied by increased beating frequencies. In summary, we demonstrated that miR-128a plays a so-far unknown role in early heart development by affecting the timing of CPC differentiation into various cardiomyocyte subtypes.

## 1. Introduction

Currently, more than 2500 microRNAs (miRs) have been detected in humans [1] and it is predicted that most mammalian mRNAs are regulated by at least one miR [2]. MiRs therefore seem to be involved in most physiological and pathophysiological processes in the body. Given this complexity, a complete picture of miR-dependent regulations in development, homeostasis, and disease is still emerging, and the specific roles of several miRs remain elusive.

The heart is the first and most important organ to develop during embryogenesis. Cardiogenesis relies on a tightly regulated and synchronized network of growth factors, cardiac transcription factors, as well as non-coding RNAs. Dozens of miRs have already been described as indispensable players in cardiac development [3,4]. Nearly all steps in the developing heart including cardiovascular lineage differentiation and cardiac cell proliferation involve regulatory actions of miRs [5,6]. MiRs exert their function by binding to specific target regions in the 3′-untranslated regions (UTRs) of mRNAs leading either to cleavage or translational repression [3]. Several studies have implicated that dysregulations of these approximately 22 nt long molecules impair cardiogenesis and might even contribute to congenital heart defects [7]. It is thus indispensable to precisely understand how miRs influence cardiac development.

To identify miRs that could play a role in early cardiogenesis, we first analyzed the miR profile of multipotent cardiac progenitor cells (CPCs) defined by an active *Nkx2.5* cardiac enhancer (NkxCE) element [8].

As one of these candidates, we describe miR-128a enriched in these CPCs. Usually highly expressed in the brain, miR-128 is primarily known for its function during neurogenesis [9], and has also been linked to the development of gliomas [10]. In the context of cardiac settings, miR-128 was differentially expressed in newt hearts after resection injuries when hyperplasia was on its peak, ascribing it a role during cardiac regeneration in lower vertebrates [11]. Additionally, a recent publication demonstrated that miR-128a was robustly upregulated in one-week old murine cardiac ventricles when compared to one day old ventricles, attributing miR-128a with a function in postnatal heart growth by modulating cardiomyocyte proliferation [12]. Furthermore, several well-known miR-128a targets, including *Isl1* and *Wnt3a* [11,13], are important regulators of early cardiac development. However, the role of miR-128a in early heart development has not been investigated in detail.

To contribute to a more complete image of miR-128a function in early cardiogenesis, we analyzed miR-128a knockdown (KO) in zebrafish (*Danio rerio*) and additionally performed miR-128a loss-of-function and gain-of-function experiments during murine pluripotent stem cell differentiation. Collectively, our data define a role of miR-128a during early cardiogenesis.

## 2. Results

### 2.1. Identification of microRNAs (miRs) Involved in Early Cardiac Development

**Figure 1:**
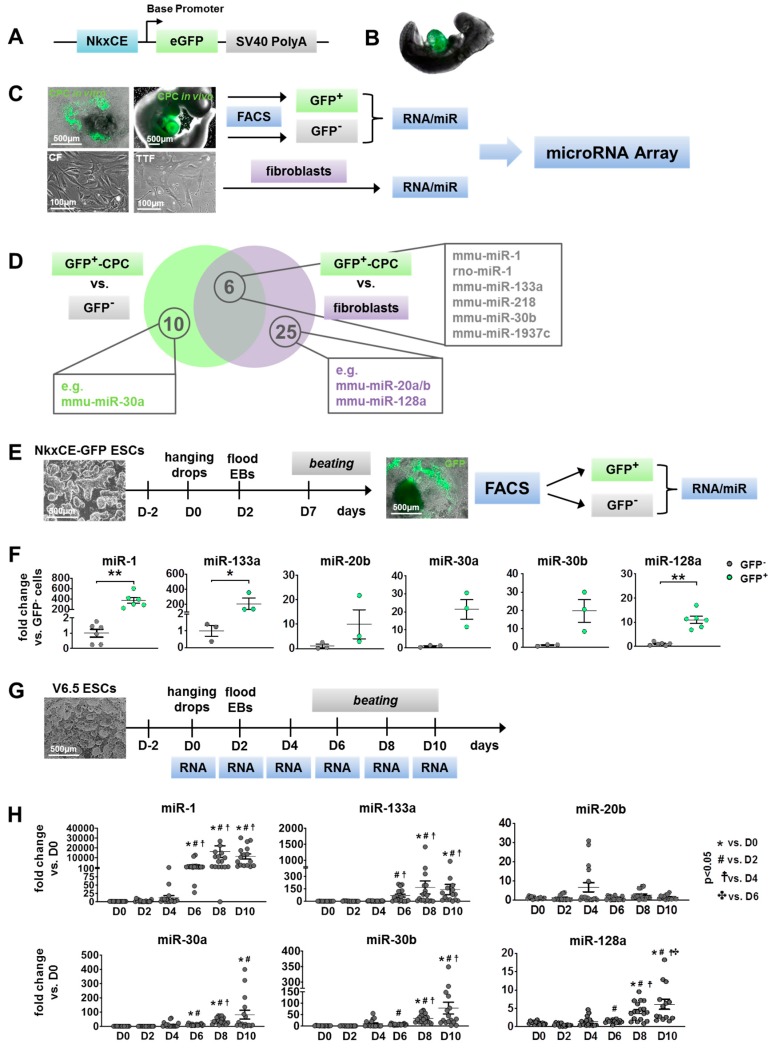
Identification of candidate microRNAs (miRs) during cardiac development. (**A**) Nkx2.5 cardiac enhancer eGFP (NkxCE-GFP) vector construct: (**B**) The cardiac specific NkxCE (~9.5kb upstream of Nkx2.5-ATG site) exclusively marks cardiac progenitor cells (CPCs) in embryonic hearts by GFP expression, as shown in an E9.5 mouse embryo. (**C**) Generation of cells for microRNA (miR) Array: GFP-positive cardiac progenitor cells (CPCs) and correspondent GFP negative cell fractions were sorted by flow cytometry (FACS) from E9.5 mouse embryos and in vitro differentiations on day 7 (D7) (upper images, scale bars: 500 µm). RNA/miR was isolated from murine tail tip fibroblasts (TTFs) and cardiac fibroblasts (CFs) (lower images, scale bars: 100 µm) for comparative analysis of miR expression profiles. (**D**) Venn diagram of upregulated miR candidates from miR Array analysis. 16 miRs were up-regulated (>1.5-fold, *p* < 0.05) in GFP-positive CPCs compared to their GFP-negative counterparts and 31 miRs were up-regulated in comparison to fibroblast populations (TTFs, CFs). Six of these miRs (e.g., miR-1, -133a, -30b, miR-218) were enriched in GFP-positive CPCs versus both GFP-negative cells and fibroblasts. (**E**) Experimental setup of in vitro differentiation (hanging drop method) of NkxCE-GFP ESCs for verification of miR candidates (scale bars: 500 µm). At differentiation day 7 (D7), GFP-positive CPCs and GFP-negative cell fractions were sorted by FACS, and total RNA was purified. (**F**) CPC marker miRs miR-1 (*n* = 6, Mann–Whitney test, *p* = 0.0022) and miR-133a (*n* = 3, *t*-test, *p* = 0.0465) were significantly upregulated at D7 in NkxCE-GFP CPCs in comparison to stage-matched negative cells, whereas only miR-128a (*n* = 6, Mann–Whitney-test, *p* = 0.0022) was significantly enriched of candidate miRs in NkxCE GFP-positive CPCs. No significance was found for miR-20b, -30a, and -30b (*n* = 3 each, *t*-test). Three assays were performed, whereas only for miR-1 and miR-128, samples were measured in duplicates. (**G**) To evaluate miR kinetics during in vitro differentiation, non-transgenic murine ESCs (V6.5, scale bar: 500 µm) were differentiated (hanging drop method) until day 10. RNA was isolated every other day for qRT-PCR. (**H**) Expression of miR-1 and -133a followed a typical course by rising at the beginning of cardiomyogenesis around D4 to D6. MiR-30a, -30b, and -128a also rose around D4 to D8. However, expression of miR-20b appears to be biphasic with a peak on D4 and D8. Three assays were performed in triplicate (*n* = 9 per timepoint; each sample was measured in duplicates *n* = 18 per timepoint; ANOVA followed by Dunn’s Method). All data are represented as means ±SEM. * *p* ≤ 0.05, ** *p* ≤ 0.01.

To identify miRs with unknown roles in early cardiac development, the miR profile of multipotent cardiac progenitor cells (CPCs) was evaluated in comparison to fibroblasts and stage-matched differentiated cells. The analyzed CPCs are characterized by an active 2.1 kb *Nkx2.5* cardiac enhancer (NkxCE) element [8,14] and are able to develop in all major cardiac cell types, namely cardiomyocytes (CMs), endothelial cells (ECs), smooth muscle cells (SMCs), and cardiac fibroblasts (CFs) [15]. In the transgenic *Nkx2.5* cardiac enhancer GFP (NkxCE-GFP) mouse, the activated NkxCE specifically marks the embryonic heart from embryonic day 8.5 (E8.5) until E15.5 [8] (Figure 1A,B).

For miR profiling, GFP-positive CPCs were isolated from transgenic NkxCE-GFP mouse embryos at E9.5 and from one week in vitro differentiated murine embryonic stem cells (ESCs) with the same transgenic background using flow cytometry (Figure 1C, Figure S1A,B). For comparison, the corresponding GFP-negative cells (stage-matched developed cells) were also collected. In addition, CFs and tail tip fibroblasts (TTFs) from adult mice were cultivated for few passages (Figure 1C, Figure S1C,D). For each cell type, expression levels of about 750 known miRs were evaluated by a Taqman Array Rodent miRNA Card Set. In comparison to fibroblasts (CFs and TTFs), 31 microRNAs were upregulated (> 1.5-fold, *p* < 0.05) in GFP-positive CPCs (from E9.5 and day seven differentiated ESCs) including miR-20a-5p, miR-20b-5p, and miR-128a-3p (Figure 1D, Figure S1E). Sixteen miRs were expressed significantly higher (> 1.5-fold, *p* < 0.05) in GFP-positive CPCs when compared to their corresponding GFP-negative cell fractions (including miR-30a-5p) (Figure 1D, Figure S1E). Six miRs were upregulated in GFP-positive CPCs compared to both groups (Figure 1D, Figure S1E). Among those were miR-1-3p, miR-133a-3p, and miR-218-5p, which are already well known for their roles in early cardiac development and thus were not further analyzed here [4,6,16], as well as miR-30b-5p and miR-1937c. The most interesting candidates from all groups were chosen after extensive literature research and a preservation analysis in different species. The sequences and seed regions of the sense (5p) or antisense strands (3p) of miR-20a, miR-20b, miR-30a, miR-30b, and miR-128a are highly conserved between species (Figure S1F), indicating that they might play important roles during developmental processes. In addition, these candidates had been associated with cardiac or at least skeletal muscle development [11,12,17,18]. MiR-20a was excluded since it is expressed from the miR-17~92 cluster, which has been previously studied extensively in the context of cardiac development [19]. Thus, for further analysis, miR-20b, miR-30a, miR-30b, and miR-128a were selected.

To verify microRNA Array results, we isolated GFP-positive CPCs and their GFP-negative counterparts from differentiated NkxCE-GFP ESCs on day seven (independent from the samples used in the microRNA Array) (Figure 1E). miR candidates’ expression as well as miR-1 and miR-133a, as controls for early cardiac development, were evaluated (Table S1). As expected, miR-1 and miR-133a were significantly enriched in GFP-positive CPCs (Figure 1F). The miR candidates (miR-20b, miR-30a, miR-30b, miR-128a) all showed higher expression in GFP-positive CPCs, however only miR-128a was significantly upregulated when compared to the GFP-negative fraction (*p* < 0.01) (Figure 1F). Next, kinetics of candidate miRs were analyzed during spontaneous in vitro differentiations. Non-transgenic murine ESCs (V6.5) were differentiated for 10 days using a standard hanging drop protocol (Figure 1G). First beating cell clusters, indicating the advent of CPCs or early cardiomyocytes were observed from day five of differentiation. Total RNA was isolated every other day and miR expression was evaluated by qRT-PCR (Figure 1G). Transcription factors with well-known roles during cardiac development such as *Gata4*, *Nkx2.5,* or *Hand1* became upregulated with the onset of cardiac specification beginning at differentiation day four to six (Figure S2A). The same holds true for miR-1 and miR-133a (Figure 1H). Three of the selected miRs (miR-30a, miR-30b, and miR-128a) followed this trend and became upregulated from day four (Figure 1H). The level of miR-20b, however, showed an expression peak on day four and a second smaller peak on day eight (Figure 1H).

Different pluripotent stem cell lines do not necessarily behave equally in vitro, e.g., due to epigenomic and transcriptomic differences or variable differentiation abilities [20]. Additionally, miR chromosomal locations and expression profiles can vary between species [21]. We therefore evaluated candidate miR kinetics in human induced pluripotent stem cells (hiPSCs). Established hiPSCs were differentiated using a protocol shifting the cells in cardiac direction by manipulating the Wnt-signaling pathway with small molecules [22] (Figure S2B). HiPSCs were differentiated until day 14 since differentiation in human cells usually proceeds slower than in murine cells and total RNA was isolated every other day. As in murine ESCs, miR-1 and miR-133a became upregulated with the onset of cardiac differentiation when first beating cells emerged (D8–D10) (Figure S2C). Our results further demonstrated that candidate miRs exhibited a similar trend of expression during human cardiac-directed differentiation compared to murine spontaneous ESC differentiation (Figure S2C and Figure 1H).

Here, we generated a miR profile of multipotent, NkxCE-GFP positive CPCs for the first time. The selected miR candidates (miR-20b, miR-30a, miR-30b, miR-128a) might play roles during cardiac development since their sequences and their expression pattern during in vitro differentiation are highly conserved across species.

### 2.2. miR-128a loss of Function-Induced Cardiac Phenotype in Zebrafish

Next, we investigated the cardiovascular impact of the selected miR candidates in zebrafish.

**Figure 2:**
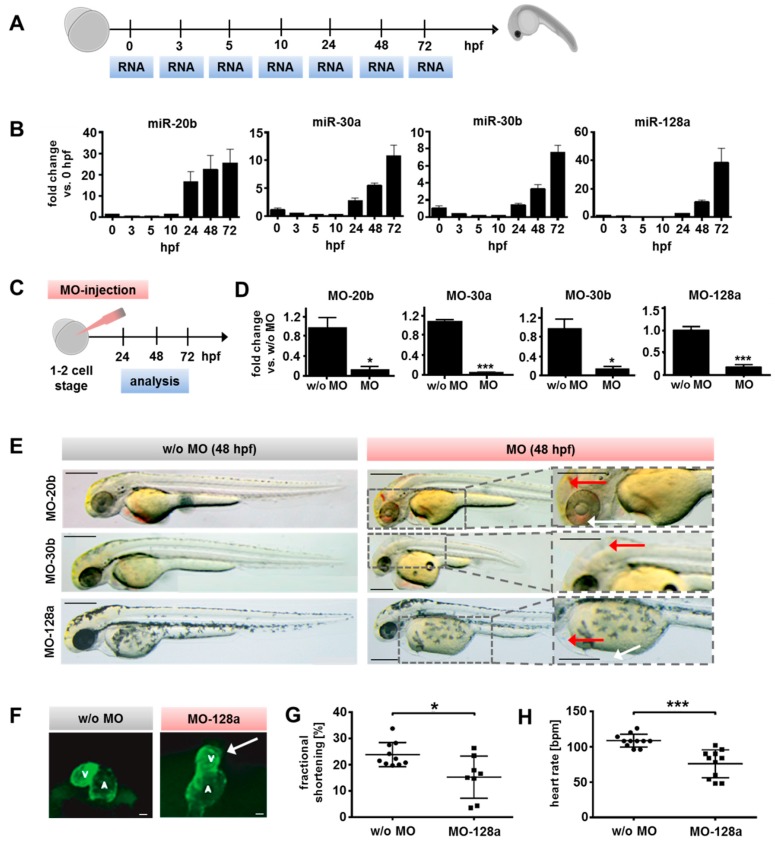
Evaluation of candidate miR function during zebrafish development. (**A**) MiR kinetics were evaluated during early zebrafish development. Total RNA was isolated on respective timepoints (hours post fertilization; hpf) for qRT-PCR. (**B**) MiR-20b, -30a, -30b, and -128a were upregulated in developing zebrafish larvae from 24 hpf increasing until 72 hpf. (**C**) Morpholino oligos (MOs) were injected in 1–2 cell-stage zebrafish embryos and miR knockdown, morphological changes, fractional shortening, and beating rate was analyzed (24, 48, 72 hpf). (**D**) MiR expression (evaluated by qRT-PCR) in zebrafish larvae was sufficiently reduced by MOs at 24 hpf by more than 85% for all candidate miRs in comparison to controls without MOs (*t*-test). (**E**) Morphological changes of MO-morphants at 48 hpf. MO-20b morphants showed cerebral hemorrhage (red arrow) and edema (edema in the eye region indicated by a white arrow). MO-30b morphants developed malformations and edemas (red arrow highlights a visible edema and enlarged hydrocephalus). MO-128a larvae exhibited a robust pericardial edema (red arrow) and blood congestion at the right outflow tract of the heart (white arrow). Scale bars: 250 µm. (**F**) Cardiac phenotype of MO-128a treated Tg(*myl7:ras*-GFP) zebrafish larvae at 48 hpf including smaller ventricles (white arrow) and abnormalities in heart looping. Scale bars: 5 µm. (**G**) MO-128a larvae (*n* = 8) showed a significantly reduced fractional shortening at 72 hpf compared to non-treated larvae (w/o MO, *n* = 10) (*t*-test) (**H**) MO-128a morphants (*n* = 11) appeared to have a significantly slower heart rate at 72 hpf compared to the controls (w/o MO, *n* = 11) (*t*-test). All data are represented as means ± SEM. * *p* ≤ 0.05, ** *p* ≤ 0.01 and *** *p* ≤ 0.001.

Kinetics of miR-20b, miR-30a, miR-30b, and miR-128a were evaluated in zebrafish larvae using qRT-PCR (Figure 2A). The expression of all miR candidates was induced with the onset of cardiac development around 24 h post fertilization (hpf) and then constantly increased until 72 hpf (Figure 2B). This matched our results obtained from in vitro differentiation of murine ESCs and hiPSCs (Figure 1H and Figure S2C) and reflects the high conservation of those miRs across species.

To further investigate specific roles of selected miRs, antisense morpholino-modified oligonucleotides (MO) complementary to the miR sequences were designed. To monitor cardiac development after miR knock-down, a transgenic zebrafish line (*Tg*(*myl7:ras*-*GFP*)) was utilized in which GFP expression is driven by the *myl7* promoter, an early cardiac marker [23]. MO injections were performed at the one or two cell stage of transgenic zebrafish embryos and larval phenotypes as well as cardiac functional parameters were analyzed (24 hpf, 48 hpf, and 72 hpf) (Figure 2C). The expression of all candidate miRs at 24 hpf was efficiently downregulated (more than 85%, *p* < 0.05) after MO injection (Figure 2D).

Subsequently, phenotypes of MO-treated larvae were investigated. At 48 hpf, MO-20b morphants showed severe brain hemorrhage (Figure 2E, red arrow, upper panel) and edema in the eye region (Figure 2E, white arrow, upper panel). Additionally, MO-20b morphants appeared to have decreased body size at 72 hpf when compared to controls, however hemorrhages and edema were diminished (Figure S3A, upper panel). Although miR-20b was involved in cardiomyocyte differentiation in vitro [18], cardiac form and function, assessed by fractional shortening measurement as an indicator for cardiac contractility, appeared normal in MO-20b treated zebrafish larvae.

MO-30a morphants did not exhibit any apparent changes in cardiac morphology from 24 hpf to 72 hpf and heart function also appeared unaffected (data not shown). MO-30b morphants developed visible edemas and enlarged hydrocephalus’s at 48 hpf (Figure 2E, red arrow). At 72 hpf, MO-30b morphants demonstrated extreme blood congestion in the inflow tract (Figure S3A; white arrow, lower panel), crimped tails (Figure S3A red arrow, lower panel), and severely shortened body length when compared to control injected larvae. The miR-30 family has previously been shown to be involved in the regulation of myogenic specification and differentiation, explaining the observed muscular malformations (crimped tails and shortened bodies) [17,24]. However, no stable functional cardiac phenotype was observed for the miR-30 family in morphant zebrafish larvae.

Morphants with knockdown of miR-128a reproducibly exhibited pericardial edemas (Figure 2E, red arrow, lower panel) and blood congestion in front of the right outflow tract indicative of impaired heart function (Figure 2E, white arrow, lower panel). Additionally, (*Tg*(*myl7:ras*-*GFP*)) larvae with reduced miR-128a consistently developed abnormalities in heart looping, which led to incorrect positioning of the heart (Figure 2F, right panel) and often smaller ventricles (Figure 2F, white arrow) at 48 hpf. However, the atrium seemed to be unaffected by miR-128a downregulation.

To monitor the impact of reduced miR-20b, miR-30b, and miR-128a levels on cardiac function, ventricular fractional shortening and heart rates of zebrafish larvae were evaluated at 72 hpf by video microscopy. For MO-20b and MO-30b morphants, no significant changes in ventricular function or heart rate were demonstrated in comparison to controls (Figure S3B,C). However, miR-128a morphants displayed a significant decrease of fractional shortening (*p* < 0.05) and heart rate (*p* < 0.001) (Figure 2G,H).

Summarizing, only the knockdown of miR-128a induced a stable and robust cardiac phenotype in zebrafish larvae accompanied by impaired cardiac function indicating an important role of miR-128a during cardiac development.

### 2.3. Knockdown of miR-128a Promoted Early Cardiogenesis and Favored the Differentiation of NkxCE-GFP CPC Populations In Vitro

To study the role of miR-128a in early cardiac development in more detail, we sought to knockdown miR-128a during in vitro differentiation of murine pluripotent stem cells. When ESCs differentiate, they closely resemble embryogenesis including cardiogenesis [8]. To monitor the frequency of early CPCs, we used previously mentioned NkxCE-GFP ESCs (Figure 1A,B).

**Figure 3:**
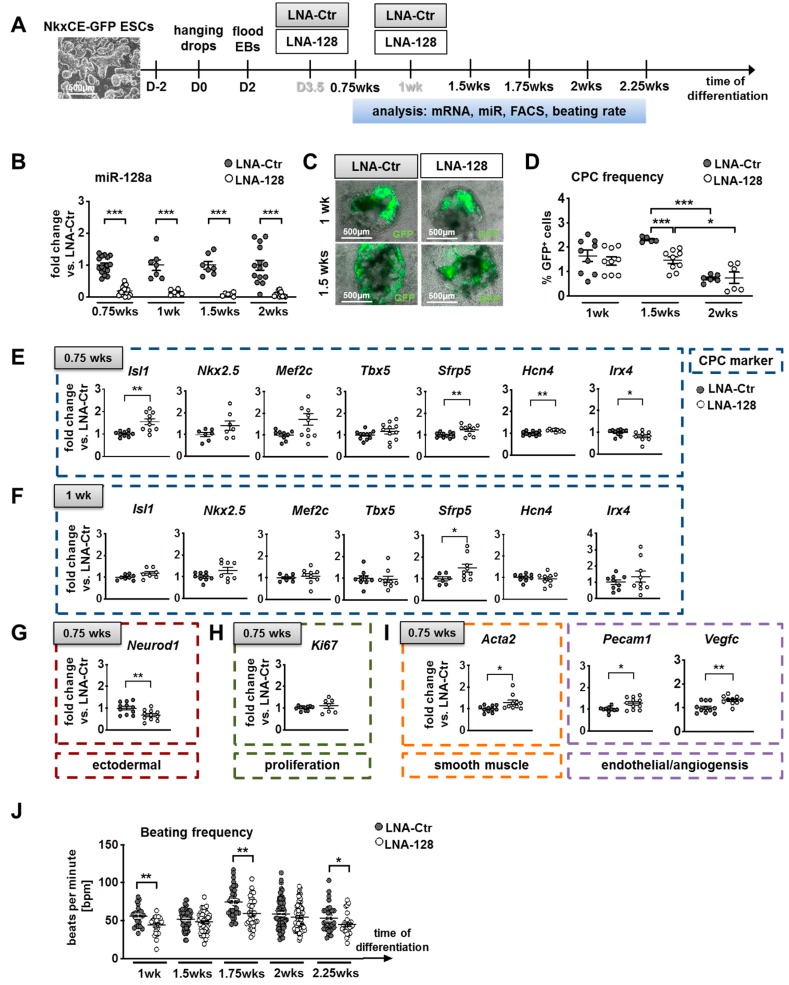
Knockdown of miR-128a during in vitro differentiation of murine NkxCE-GFP embryonic stem cells (ESCs). (**A**) NkxCE-GFP ESCs (scale bar: 500 µm) were differentiated using the hanging drop method. Transfections of locked nucleic acid (LNA) probes for knockdown of miR-128a (LNA-128) and corresponding non-targeting controls (LNA-Ctr) were performed on day 3.5 (D3.5) and after one week (wk) of differentiation. Analysis was performed on respective timepoints between 0.75 and 2.25 wks. Abbreviations: EB: Embryoid bodies; FACS: Flow cytometry; (**B**) MiR-128a was significantly downregulated (*p* < 0.001, Mann–Whitney test) by LNA-128 from 0.75 wks until 2 wks when compared to respective LNA-Ctrs. At least 3 assays in triplicates were performed per time-point. (**C**) NkxCE-GFP-positive CPCs after 1 wk (upper panel, scale bars: 500 µm) and 1.5 wks (lower panel, scale bars: 500 µm) after transfection with either LNA-Ctr or LNA-128. The images are an overlay between phase contrast and fluorescent pictures. (**D**) The frequency of NkxCE*-*GFP-positive CPCs with miR-128 knockdown (*n* = 9) was slightly reduced after 1 wk, becoming significantly downregulated after 1.5 wks (*n* = 5, 9; *p* = 0.001, Mann–Whitney test). The CPC frequency after 2 wks was not affected by LNA-128 compared to LNA-Ctr (*n* = 6). 3 assays in triplicates for 1 wks and 1.5 wks, 2 assays in triplicates for 2 wks. (**E**–**I**). Gene expression panels during NkxCE-GFP ESC differentiation after miR-128 knockdown. The panels show the expression of early CPC markers after 0.75 wks (*n* = 11, 4 assays in triplicates, *t*-test or Mann–Whitney test) (**E**) and 1 wk (*n* = 9, 3 assays in triplicates, *t*-test or Mann–Whitney test) (**F**) as well as neuroectodermal (*t*-test) (**G**), proliferation (**H**), smooth muscle (Mann–Whitney test), endothelial, and angiogenesis markers (*t*-test) (**I**) after 0.75 wks. (**J**) Beating frequency (beats per minute, bpm) of early cardiomyocytes was downregulated from 1 wk to 2.25 wks on mir-128a knockdown compared to LNA-Ctr (*n* > 23, *t*-tests) (at least 4 assays, for 2.25 wks 2 assays, 8 videos were evaluated per condition from 3 independent observers). Data are represented as means ± SEM. * *p* ≤ 0.05, ** *p* ≤ 0.01 and *** *p* ≤ 0.001.

NkxCE-GFP ESCs were differentiated by a standard hanging drop protocol (Figure 3A). Locked nucleic acid (LNA) probes were added during differentiation together with a transfection reagent to knockdown miR-128a (LNA-128) (Figure 3A). LNA probes as well as transfection reagents might have an impact on cell physiology (e.g., cell proliferation as assessed by MTT or CPC frequency detected by flow cytometry) (Figure S4A–D). Therefore, we used cell samples transfected with LNA-probes without homology to miRs of any species as a control (LNA-Ctr) (Figure 3A). All results derived from in vitro assays with LNA-128-probes were normalized to the respective LNA-Ctr transfected groups.

To ensure that miR-128a would be downregulated at its onset of expression at differentiation day four (Figure 1H), we added LNA-128 probes at day 3.5. To further ensure a stable knockdown of miR-128a, a second transfection with LNA-probes was performed after one week (1 wk). qRT-PCR for gene- and miR-expression analysis (Tables S1 and S2), flow cytometry (FACS) for evaluating CPC-frequency, and video microscopy for estimating the beating frequency of CPCs or early cardiomyocytes (Figure 3A) was performed between 0.75 wks and 2.25 wks.

MiR-128a expression was significantly reduced by LNA-128-probes by more than 80% (*p* < 0.001) when compared to the LNA-Ctr groups (Figure 3B).

Since miRs might play important roles in cell fate decisions and lineage commitment, including the specification of mesodermal cells to CPCs [4,25], we next monitored the impact of miR-128a knockdown on the frequency of NkxCE-GFP-positive CPCs during differentiation by flow cytometry. GFP-positive CPCs developed regularly in all LNA-treated (LNA-Ctr and LNA-128) embryoid bodies (EBs) from 0.85 weeks (day 6) and started beating spontaneously (Figure 3C; Videos V1–V3). Interestingly, NkxCE-GFP-positive CPCs decreased significantly in LNA-128 treated EBs compared to LNA-Ctr treated EBs after 1.5 weeks (37% fewer CPCs than in LNA-Ctr; *p* < 0.001; Figure 3D). Namely, the Nkx-enhancer and, by this also, the GFP expression become inactive on further maturation of CPCs as can be clearly seen by the diminished percentage of GFP-positive CPCs after two weeks of differentiation in the LNA-Ctr treated EBs (*p* < 0.0001 compared to 1.5 wks) (Figure 3D). After two to three weeks of differentiation, cardiomyocytes (CMs), endothelial cells (ECs), and smooth muscle cells (SMCs) were commonly observed during differentiation (Figure S4E).

To identify a mechanism of action of miR-128a in this cardiac context, we first sought to analyze predicted target genes of miR-128 by Target Scan version 7.2 (http://www.targetscan.org [26]) and miRDB (http://mirdb.org [27,28]). To detect conserved targets, we each analyzed target genes of mmu-miR-128-3p (mouse) and hsa-miR-128-3p (human) (miR-128a). Between 988 and 1254 target genes were identified (Table S2). We further performed a Venn diagram analysis to obtain only those targets that appeared in all four lists and ended up with 476 target genes (Figure S5A). For those genes, we further accomplished a gene set enrichment analysis (GSEA 4.0, http://software.broadinstitute.org/gsea [29,30]). The GO term “Cardiovascular System development” was enriched with a *p*-value of 7.97∙10^−17^ (FDR q-value of 1.5∙10^−14^) and included 45 potential target genes. We further performed a gene annotation enrichment analysis (David Bioinformatics Resources 6.8 (https://david.ncifcrf.gov/home.jsp [31,32])) and received the following GO-terms corresponding to heart development: Heart development, outflow tract morphogenesis, cardiac atrium morphogenesis, ventricular cardiac muscle differentiation, and positive regulation of cardiac muscle differentiation (Figure S5A). We picked out eight interesting genes and analyzed their gene expression at respective timepoints (*Isl1*, *Sfrp1*, *Dvl2*, *Tgfbr1*, *Acta2*, *Gsk3b*, *Vegfc, and Hcn4;*). In addition, several key transcription factors for heart development and cardiac lineage markers were analyzed.

During mouse embryonic development, CPCs start to express key transcription factors, i.e., *Nkx2.5*, *Isl1*, *Sfrp5,* or *Irx4* at approximately E7.5 (~ differentiation day five (0.75 wks) in vitro) thereby committing irreversibly to their cardiac fate [8,33,34]. Interestingly, *Isl1*, as a confirmed as well as predicted target of miR-128 [11], was significantly upregulated after 0.75 weeks (*p* < 0.01) (Figure 3E) with a continued trend of upregulation until week two (Figure 3F, Figure S5B,C). Other cardiac progenitor markers including *Nkx2.5*, *Mef2c,* and *Tbx5* only showed a trend to be upregulated after 0.75 weeks (Figure 3E) and no differences were observed during further differentiation (Figure 3F, Figure S5B,C). However, *Sfrp5* expressed by progenitor cells mainly contributing to the left ventricle, *sinus venosus,* and parts of the atria [33], and the early first heart field progenitor cell marker *Hcn4* [35]—also a predicted target of miR-128a—were significantly upregulated after 0.75 weeks (*p* < 0.01) (Figure 3E). Interestingly, *Irx4*, which specifically marks ventricular progenitors [36], was significantly decreased at this timepoint (*p* < 0.05) (Figure 3E). Most of the mentioned cardiac genes were not differentially expressed at later timepoints during differentiation (Figure 3F, Figure S5B,C), indicating that miR-128a only acts during early cardiogenesis.

However, further predicted targets of miR-128a, such as *Gsk3b*, *Dvl2*, and *Sfrp1* being involved in Wnt-signaling which is important for cardiovascular development [37,38], or *Tgfbr1* (also relevant for cardiovascular development [39]) were not influenced by a knockdown of miR-128 in murine ESCs (Figure S5D–G).

Other lineage markers, such as *Neurod1*, reflecting ectodermal progenitor cells, were significantly reduced after 0.75 weeks (*p* < 0.01) (Figure 3G), promoting the idea of an early stimulation of mesendodermal cells, for example certain CPC lineages. The expression of the proliferation marker *Ki67* did not alter between the miR-128a knockdown and LNA-Ctr groups after 0.75 weeks (Figure 3H).

To further investigate the idea that miR-128a loss-of-function might favor cardiac differentiation of certain CPC lineages, we evaluated the expression pattern of specific markers for ECs (*Pecam1*), SMCs (*Acta2*, a predicted target of miR-128a), CMs *(Tnnt2*, *Myh6*), atrial CMs (*Myl7*), and ventricular CMs (*Myl2*) during in vitro differentiation (Figure 3I, Figure S6A–C). Interestingly, we found *Acta2* and *Pecam1* expression significantly increased after 0.75 weeks (*p* < 0.05) (Figure 3I). In addition, *Vegfc*, another predicted target of miR-128a and a marker for angiogenesis, was also significantly increased after 0.75 weeks. Recently, RNA sequencing of NkxCE-GFP-positive CPCs from embryonic hearts was performed in comparison to cardiomyocytes from adult mouse hearts and revealed that early multipotent NkxCE-CPCs expressed *Acta2* as well as *Pecam1* and *Vegfc* at high levels [40] *(*Figure S6D). However, at later timepoints, no effects on *Acta2*, *Pecam1,* or *Vegfc* expression were found (Figure S6A–C, left panels).

Concerning general cardiomyocyte lineages, neither *Tnnt2* nor *Myh6* were regulated significantly on miR-128a knockdown (Figure S6A–C). The expression of the atrial cardiomyocyte marker *Myl7* also appeared similar in LNA-128 samples and controls (Figure S6A–C). However, levels of the ventricular marker *Myl2* were elevated throughout differentiation, becoming significant after 1.5 weeks (*p* < 0.05, Figure S6A–C).

Next, we wanted to see if functional parameters of early cardiomyocytes were also altered on miR-128a knockdown. We therefore analyzed the frequency of beating foci at several timepoints using video microscopy (Figure 3A). A general reduction of beating frequency of early cardiomyocytes was observed at all evaluated timepoints, which was significantly reduced after one week (*p* < 0.01), 1.75 (*p* < 0.01), and 2.25 weeks (*p* < 0.05) (Figure 3J). Neither *Hcn4*, nor *Shox2* expression (markers for fast-firing nodal-like cardiomyocytes), were significantly impacted by miR-128a knockdown (Figure S6E).

Gene expression results regarding differentiation of CPC populations into cardiac lineages indicated that miR-128a loss-of-function did not influence endothelial or smooth muscle cell differentiation. Rather, cardiomyocytes of the “working myocard”, especially marked by the ventricular marker *Myl2*, were affected. This was supported by the slower beating frequency observed in early cardiomyocytes.

### 2.4. Knockdown of miR-128a Favoured Isl1-Positive CPCs In Vitro

Given the previous results implicating that miR-128a knockdown influenced early cardiogenesis (0.75 wks of in vitro differentiation), including an upregulation of *Isl1* expression, we sought to analyze miR-128a knockdown in Isl1-reporter-iPSCs. We generated iPSCs from fibroblasts of the transgenic *Isl1*-Cre/Rosa26^mT/mG^ (ITG) mouse that ubiquitously expresses membrane-tagged tdTomato in all cells (mT) and labels *Isl1*-expressing cells by membrane-tagged GFP-expression (mG) upon Cre-mediated excision of tdTomato [41,42] (Figure S7A). Transgenic tail tip fibroblasts (TTFs) were reprogrammed by a polycistronic doxycycline-inducible (tet-on) lentiviral construct containing the four “Yamanaka” factors (*Sox2*, *c-Myc*, *Oct4*, *Klf4*) [43,44] (Figure S7B,C). IPSC colonies were picked and further expanded (Figure S7C,D). Pluripotency and the ability to differentiate in all three germ layers of iITG-iPSCs were verified (Figures S7E–G and S8A,B). Development of GFP-positive *Isl1*-CPCs occurred with the onset of cardiac gene expression between differentiation week 1 to 1.5, accompanied by the occurrence of beating clusters around week 1.75. Despite having tested several iPSC clones, this was approximately one week later than differentiating NkxCE-GFP-ESCs usually expressed cardiac and other lineage genes and started beating (Figure S8C,D). This might be due to a decreased expression of *Isl1* in iITG-iPSCs, since the Cre is knocked into the *Isl1* locus, replacing the endogenous *Isl1* ATG [42].

**Figure 4:**
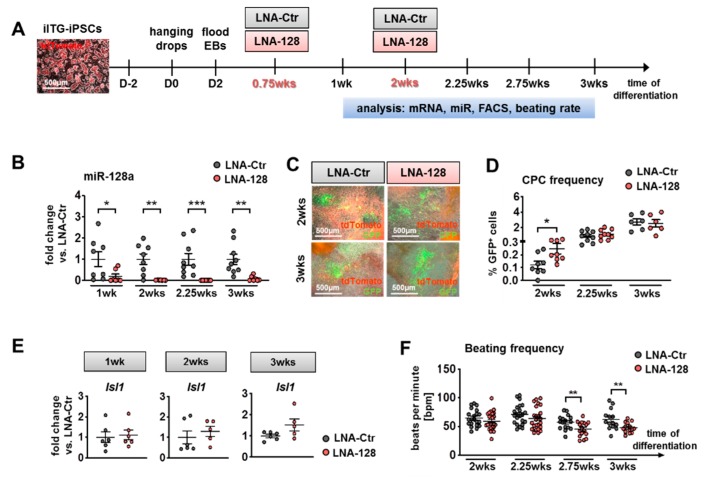
Knockdown of miR-128a during in vitro differentiation of murine Isl1-Cre/ROSA26^mTmG^ induced pluripotent stem cells (iPSCs) (iITG-iPSCs). (**A**) iITG-iPSCs (scale bar: 500 µm) were differentiated using the hanging drop method. Transfections of LNA probes for knockdown of miR-128a (LNA-128) and corresponding controls (LNA-Ctr) were performed after 0.75 weeks (wks) and after 2 wks of differentiation. Analysis was performed on respective timepoints between 1 and 3 wks. Abbreviations: EB: Embryoid bodies; FACS: Flow cytometry; (**B**) MiR-128a was significantly downregulated by LNA-128 from 1 wk until 3 wks compared to respective LNA-Ctr (*n* = 8,7, Mann–Whitney tests, 3 assays in triplicates). (**C**) *Isl1*-GFP-positive CPCs after 2 wks (upper panel, scale bars: 500 µm) and after 3 wks (lower panel, scale bars: 500 µm) of in vitro differentiation after transfection with either LNA-Ctr or LNA-128. Images are an overlay between phase contrast and fluorescent microscopic pictures. (**D**) The frequency of *Isl1*-GFP-positive CPCs after miR-128a knockdown was significantly increased to the double amount compared to LNA-Ctr (*n* = 9, *p* = 0.024, *t*-test; 3 assays in triplicates) after 2 wks. At later timepoints, the *Isl1*-CPC frequency was not significantly affected by miR-128 knockdown (3 assays in triplicates at 2.25 wks, 2 assays in triplicates at 3 wks). (**E**) *Isl1* expression was tendentially upregulated during iITG-iPSC differentiation after miR-128 knockdown (*n* = 6, 2 assays in triplicates). (**F**) Beating frequency (beats per minute, bpm) of differentiated iITG-iPSC EBs was reduced after miR-128 knockdown (2 and 2.25 wks: 3 assays in triplicates, 2.75 (*t*-test) and 3 wks (Mann–Whitney test): Two assays in triplicates, 4–9 videos per condition, 3 independent observers). Data are represented as means ± SEM. * *p* ≤ 0.05, ** *p* ≤ 0.01 and *** *p* ≤ 0.001.

Transfection of LNA-probes in the iITG-iPSCs was therefore conducted at 0.75 wks and after two weeks of differentiation (Figure 4A). Analysis of gene and miR expression, as well as CPC occurrence and beating frequency, was performed between one and three weeks (Figure 4A). Efficient knockdown of miR-128a by more than 80% was confirmed by qRT-PCR after transfection with LNA-128 probes when compared to the corresponding LNA-Ctrs (Figure 4B). *Isl1*-positive CPCs started to appear as expected after 1 to 1.5 weeks in LNA-treated EBs (LNA-Ctr and LNA-128) accompanied by spontaneous beating (Figure S8D, left panels, Figure 4C, Videos V4–V5).

GFP-positive *Isl1*-CPCs accumulated during differentiation, since the cut-out of tdTomato in front of GFP by the Cre-system led to a permanent labeling of *Isl1*-positive CPCs and their progeny (Figure 4D) (Figure S7A). Interestingly, the double amount of *Isl1*-GFP-positive CPCs (*p* < 0.05) was detected on miR-128a knockdown in comparison to the LNA-Ctr treated group during early cardiogenesis (after two weeks) (Figure 4D). In addition, *Isl1* gene expression tendentially increased throughout differentiation from week one to three after miR-128a knockdown (Figure 4E). Early cardiomyocytes of *Isl1*-reporter-iPSCs also showed a reduced beating frequency on miR-128a knockdown (*p* < 0.01, from 2.75 wks on) (Figure 4F).

The above described results strengthen our hypothesis that *Isl1*-positive CPCs are favored by miR-128a knockdown during early cardiogenesis.

### 2.5. Overexpression of miR-128a Suppressed Early Cardiogenesis and Retarded Differentiation of NkxCE-GFP-Positive CPC Populations In Vitro

Next, we sought to investigate the effect of miR-128a overexpression (OE) during in vitro differentiation of murine pluripotent stem cells. We therefore generated stable, doxycycline-inducible miR-OE ESCs by transduction of NkxCE-GFP ESCs with lentiviruses containing constructs that are either overexpressing miR-128a (OE-128) or a non-targeting miR-Ctr (OE-Ctr) (Figure S9A). Addition of doxycycline (dox) should switch on miR-Ctr or miR-128a expression accompanied by turbo red fluorescent protein (tRFP) expression (Figure S9A, tRFP is located in front of miR-128a or miR-Ctr). By using NkxCE-GFP ESCs for transductions we were able to detect CPC frequency upon miR-128-OE during differentiations.

After transduction of NkxCE-GFP ESCs, dox-induction as well as puromycin selection was performed (Figure S9B). MiR-OE ESC colonies visually expressing high levels of RFP were subsequently picked and expanded (Figure S9B,C). For further selection of suitable miR-OE ESC clones, maintenance of pluripotency as well as the induction of RFP and miR-128a expression were evaluated (Figure S9D–F). Immunostaining against Sox2 and Nanog showed that generated miR-OE ESCs expressed both proteins similar than the originating NkxCE-GFP ESCs (positive control) (Figure S9D). MiR-OE ESCs (OE-Ctr as well as OE-128) cultivated with doxycycline showed a significant increase (*p* < 0.001) of RFP-positive cells detected by flow cytometry compared to their correspondent controls without doxycycline (Figure S9E). When we validated miR-128a expression on doxycycline induction, we meaningfully did not find elevated levels in OE-Ctr ESCs without, nor with doxycycline (Figure S9F). However, miR-128a expression in OE-128 ESCs was already elevated without doxycycline (*p* < 0.05) compared to the OE-Ctr ESCs (we tested several clones) (Figure S9F). This kind of leakiness of tet-on systems (expression of miR-128 w/o dox) has been described extensively before and is most likely due to basal regulation of the tet-promoter [45,46]. Unfortunately, miR-128a expression was not significantly elevated in OE-128 ESCs on doxycycline addition (even if 2 µg/mL were used) when compared to OE-128 ESCs without doxycylin (Figure S9F), although the number of RFP-positive cells rose significantly (Figure S9E). This indicates that not all RFP-positive cells expressed miR-128a at a sufficient level after doxycycline induction (RFP is located in front of miR-128, Figure S9A). Reduced expression of the second gene from one promoter is unfortunately also observed commonly [47].

In general, the use of tetracyclines in cell culture might be critical since an impact on cell metabolism, proliferation, and gene expression has been discussed widely [48]. We therefore evaluated doxycycline influences on miR-OE ESC proliferation. We showed, by means of MTT assays, that addition of doxycycline did not significantly change proliferation of miR-OE ESCs (Figure S9G). However, when evaluating the effect of doxycycline during differentiation of miR-OE-Ctr ESCs, gene expression of important cardiac transcription factors as well as NkxCE-GFP CPC frequency was significantly changed by doxycycline, especially at later timepoints (>1.5 weeks) (Figure S10A–D).

**Figure 5:**
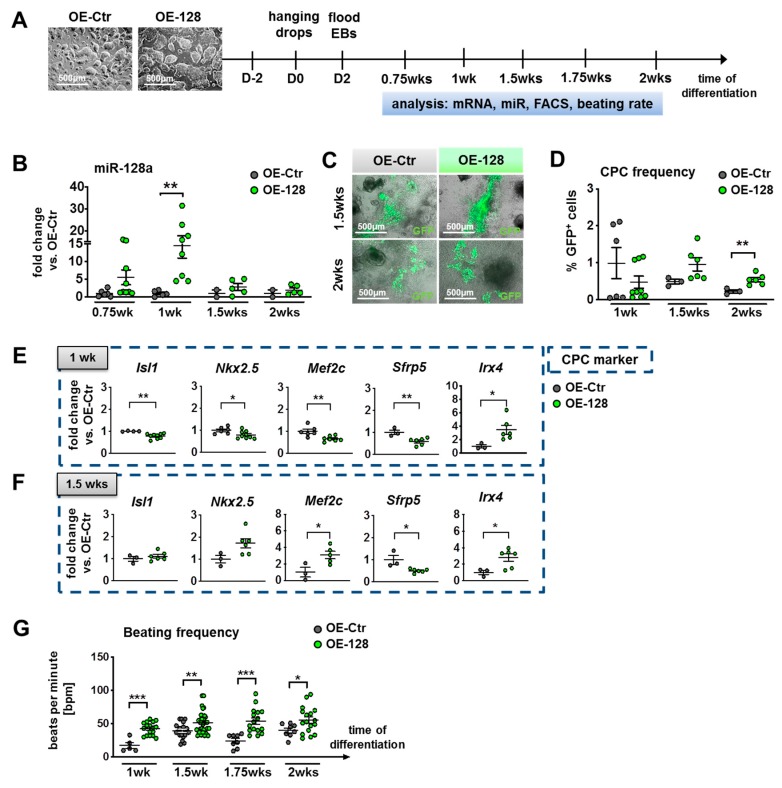
Overexpression (OE) of miR-128a during in vitro differentiation of murine NkxCE-GFP OE-128 and OE-Ctr ESCs. (**A**) OE-128 and OE-Ctr ESCs (scale bars: 500 µm) were differentiated for 2 weeks (wks) by the hanging drop method (without doxycycline). Analysis was performed on respective timepoints between 0.75 and 2 wks of differentiation. Abbreviations: EB: Embryoid bodies; FACS: Flow cytometry; (**B**) MiR-128a was overexpressed in OE-128 EBs compared to OE-Ctr EBs from early onset of cardiogenesis (0.75 wks), becoming significant after 1 wk (*p* < 0.01, Mann–Whitney test); 0.75 and 1 wk: 3 assays in triplicates, different clones; 1.5 and 2 wks: 2 assays in triplicates, different clones. (**C**) NkxCE-GFP-positive CPCs in OE-Ctr EBs or OE-128 EBs after 1.5 wks (upper panel, scale bars: 500 µm) and after 2 wks (lower panel, scale bars: 500 µm). Images are an overlay between phase contrast and fluorescent microscopic pictures. (**D**) NkxCE GFP-positive CPC frequency after miR-128 OE appeared to be reduced after 1 wk, whereas the frequency of NkxCE*-*GFP-positive CPCs slightly increased after 1.5 wks upon miR-128 OE becoming significant after 2 wks (*p* = 0.0088, *t*-test). 1 wk: 3 assays in triplicates (different clones), 1.5 and 2 wks: 2 assays in triplicates (different clones). (**E**,**F**) Gene expression panels during NkxCE-GFP ESC OE-128 and OE-Ctr differentiation. The panels show the expression of early CPC markers after 1 wk (3 assays in triplicates, different clones) (*t*-tests) (**E**) and 1.5 wks (2 assays in triplicates, different clones) (*t*-tests). (**F**,**G**) The beating frequency (beats per minute, bpm) of early cardiomyocytes was significantly upregulated from 1 wk to 2 wks on mir-128a OE compared to OE-Ctr EBs (2 assays in triplicates, different clones, 5–9 videos per timepoint) (*t*-tests or Mann–Whitney test). Data are represented as means ± SEM. * *p* ≤ 0.05, ** *p* ≤ 0.01 and *** *p* ≤ 0.001.

Due to the above-mentioned problems (leakiness of the promoter and doxycycline influences during differentiation), we finally decided to perform further experiments without addition of doxycycline (the experimental design is shown in Figure 5A). Sufficient overexpression of miR-128a could be demonstrated in differentiating OE-128 ESCs without doxycycline compared to OE-Ctr ESCs (Figure 5A,B). However, after 1.5 weeks of differentiation, robust miR-128a overexpression could not be maintained in OE-128 ESCs compared to OE-Ctr ESCs, which is possibly due to promoter methylation during differentiation [49] or other regulatory effects.

Nevertheless, miR-OE ESCs differentiated in a normal manner and developed GFP-positive CPCs starting from day six or seven accompanied by beating around day seven/eight (1 wk) (Figure 5C). In comparison to the originating NkxCE-GFP ESC line, however, their differentiation happened to be delayed about one or two days.

Interestingly, twice as many NkxCE-GFP-positive CPCs were observed in OE-128 EBs compared to OE-Ctr EBs in week 1.5, which became significant after two weeks (*p* < 0.01) (Figure 5D). Gene expression of evaluated CPC markers followed the opposite trend of regulation than in miR-128a-knockdown experiments (Figure 3E and Figure 5E,F). Overexpression of miR-128a led to a significant downregulation of *Isl1-*, *Nkx2.5-*, *Mef2c-*, and *Sfrp5* expression (*p* < 0.05) during early cardiogenesis after one week of differentiation, whereas *Irx4* was found to be significantly upregulated (*p* < 0.05) (Figure 5E). For *Sfrp5* as well as *Irx4*, this trend continued throughout differentiation (Figure 5F, Figure S11A).

Interestingly, cardiomyocyte markers *Tnnt2* and *Myh6* were significantly upregulated (*p* < 0.05) from 1.5 weeks of differentiation on miR-128-overexpression (Figure S11B,C), perhaps reflecting the higher number of NkxCE-GFP-positive CPCs (Figure 5D). As we know from aforementioned RNAseq of embryonic heart derived NkxCE-GFP-positive CPCs, these already express high levels of sarcomeric markers although not as high as mature cardiomyocytes (Figure S11D). By contrast, the ventricular cardiomyocyte marker *Myl2* was not regulated during differentiation of miR-OE EBs (Figure S11B,C). However, significant higher levels of the nodal cardiomyocyte marker *Shox2* (*p* < 0.05) were observed (Figure S11E) in OE-128 EBs compared to OE-Ctr EBs. The expression levels of smooth muscle cell marker *Acta2* and endothelial cell marker *Pecam1* did not alter upon miR-128a overexpression (Figure S11F). Interestingly, the beating frequency (bpm) of early cardiomyocytes from one to two weeks of differentiation of OE-128 EBs was significantly elevated when compared to OE-Ctr EBs (Figure 5G).

In summary, overexpression of miR-128a led to a suppression of CPC markers at the early onset of cardiogenesis (after one week of differentiation). NkxCE-GFP-positive CPCs were upregulated after 2 weeks of differentiation in OE-128 EBs compared to controls indicating a delayed differentiation of these CPCs. Finally, OE-128 EBs resulted in an increased expression of nodal-like cardiomyocyte markers (*Tnnt2-*, *Mhy6-,* and *Shox2*-positive), which was accompanied by a higher beating frequency.

## 3. Discussion

We analyzed the miR profile of a multipotent early cardiac progenitor cell (CPC) population marked by an activated cardiac-specific *Nkx2.5* enhancer (NkxCE). Several miRs have been implicated to regulate the formation of mesodermal cells, their specification into CPCs, and the differentiation of CPCs into mature cardiac lineages such as ECs, SMCs, and CMs. miR-1 and miR-133, for example, promote early mesoderm formation but suppress CPC specification during later stages of development [4]. Interestingly, we found miR-1 and miR-133 upregulated in NkxCE-positive CPCs compared to developmental stage-matched cells as well as fibroblasts. Due to this, we were highly optimistic that further identified miR candidates might also be important during cardiogenesis.

Since the sequences of promising candidates (miR-20b, miR-30a/b, and miR-128a) are highly conserved across species (zebrafish, mouse, chicken, humans), we first analyzed their expression kinetics during in vitro differentiations (mouse, human) as well as during zebrafish development. Our results showed that miR-30a/b and miR-128a each rose with the onset of early cardiogenesis, a typical kinetic that has previously been described for cardiac-specific miRs [4]. In contrast, miR-20b expression appeared to be biphasic. Similar kinetics have been shown for various cardiac transcription factors [50,51,52]. We therefore assumed that miR-20b, miR-30a/b, and miR-128a might be involved in regulatory processes of cardiogenesis since most vertebrates share a similar heart morphogenesis [53,54].

Morpholino-induced knockdown of the candidate miRs in zebrafish larvae revealed that only miR-128a caused robust cardiac malformations accompanied by functional impairment. Such phenotypes have been described in various gene-manipulating studies during zebrafish development and have been linked to congenital heart disease [7,55]. Recently however, when miR-128 was cardiac-specifically knocked out in *Nkx2.5*-Cre miR-128 floxed mice (*Nkx2.5*^Cre^;miR-128^fl/fl^ → miR-128^−/−^), neither heart size nor cardiac function was changed in adult mouse hearts [12]. However, postnatal cardiomyocytes were smaller and still highly proliferative compared to age-matched control mice [12]. These results were contrary to our findings in MO-128 injected zebrafish larvae at 48 hpf. This discrepancy might be due to different endpoints of the experimental analysis (larval vs. postnatal/adult analysis) [56]. In addition, even if cardiac development of zebrafish closely resembles that of mice [56], species-specific differences cannot be excluded. Since in vivo models remain highly complex, the nonexistent penetrant phenotype in adult mice [12] could also be attributed to compensatory mechanisms where correct cardiac development and heart function, contrary to zebrafish, is an essential requirement for overall survival [54,57]. In addition, it was shown that stable knockouts induce the activation of compensatory pathways, which was not observed after translational or transcriptional knockdown, resulting in dramatic discrepancies in phenotypes [58].

This phenotype discrepancy between miR-128 knockout mice and zebrafish was then resolved by a more detailed analysis of miR-128a function during in vitro differentiation of murine pluripotent stem cells (ESCs, iPSCs). Pluripotent stem cells are able to precisely mimic early stages of embryogenesis during in vitro differentiation and have often been used for detailed studies of transcription factor function or miR function during embryogenesis [4,51,59,60].

Thus, during murine in vitro differentiation, we demonstrated that LNA-mediated miR-128a knockdown in differentiating ES/iPSCs (1) increased cardiac transcription factors such as *Isl1*, *Sfrp5*, *and Hcn4*, but reduced *Irx4* at the onset of cardiogenesis; and (2) upregulated *Isl1*-positive CPCs whereas NkxCE-GFP-positive CPCs were downregulated. miR-128a has previously been shown to directly target the 3′UTR of *Isl1* [11], explaining the afore mentioned effects. The reduction of NkxCE-GFP-positive CPCs by LNA-128 knockdown could be a result of an enhancement of other progenitor cell populations (e.g., *Isl1*^+^ CPCs) or point to an earlier differentiation of NkxCE-GFP CPCs in cardiac lineages such as cardiomyocytes, endothelial cells, or smooth muscle cells. We did not find altered expression of the proliferation marker *Ki67* in samples with miR-128a-knockdown or controls implying that effects were not caused by a change of progenitor cell proliferation rates as indicated by Huang et al. for postnatal cardiomyocytes [12], but due to other regulatory influences of the miR-128a knockdown. Taken together, our results suggest that miR-128a might regulate CPCs at the very early onset of cardiac development in vitro (day five, week 0.75) by stimulating *Isl1/Sfrp5/Hcn4*-expressing CPCs (maybe also expressing *Nkx2.5*, *Mef2c,* and *Tbx5*) as well as inhibiting *Irx4*-expressing CPCs by this also leading to an earlier differentiation of those CPCs into cardiac lineages.

We found typical lineage marker expression for smooth muscle cells (*Acta2)*, and endothelial cells *(Pecam1*, *Vegfc)* significantly increased in miR128-knockout samples at the onset of cardiogenesis (0.75wks). This is in accordance with RNA sequencing data of NkxCE-GFP-positive CPCs from embryonic hearts that also expressed *Acta2* as well as *Pecam1* and *Vegfc* at high levels [40]. This mirrored the multipotent character of NkxCE-GFP-CPCs and emphasized that NkxCE-positive CPCs were favored by miR-128a knockdown after 0.75 weeks when they are still at their multipotent stage. However, at later timepoints, no effects on *Acta2*, *Pecam1,* or *Vegfc* expression was found indicating that endothelial as well as smooth-muscle cell lineages might not be affected by miR-128a.

Furthermore, LNA-mediated miR-128a knockdown in differentiating ES/iPSCs (3) did not affect the expression of the atrial cardiomyocyte marker *Myl7* in miR-128a knockdown samples throughout differentiation suggesting that miR-128a knockdown does not influence atrial cardiomyocytes. This is in accordance with results from zebrafish, where the atrium was unaffected by miR-128a knockdown. However, LNA-mediated miR128-KO increased the expression of the ventricular cardiomyocyte marker *Myl2* accompanied by a reduced beating frequency of early cardiomyocytes indicating an upregulation of ventricular cardiomyocytes. The reduced beating frequency of early cardiomyocytes again reflected the results from zebrafish in vivo (reduced heart rate on miR-128 KO). The heartbeat in general is controlled by fast-firing nodal cells at the sinoatrial node (SAN), expressing *Hcn4* or *Shox2*, by overriding electrical impulses of other, slower firing cells (e.g., cells of the working myocard including atrial and ventricular myocytes) [61,62]. The observed increase of *Myl2* expression could contribute to the slower beating frequency in LNA-128-treated EBs by expanding the amount of “working myocard” cardiomyocytes. Nodal-like cardiomyocytes, as demonstrated by unaffected *Hcn4-*, and *Shox2* expression, were not significantly impacted by miR-128a knockdown.

**Figure 6:**
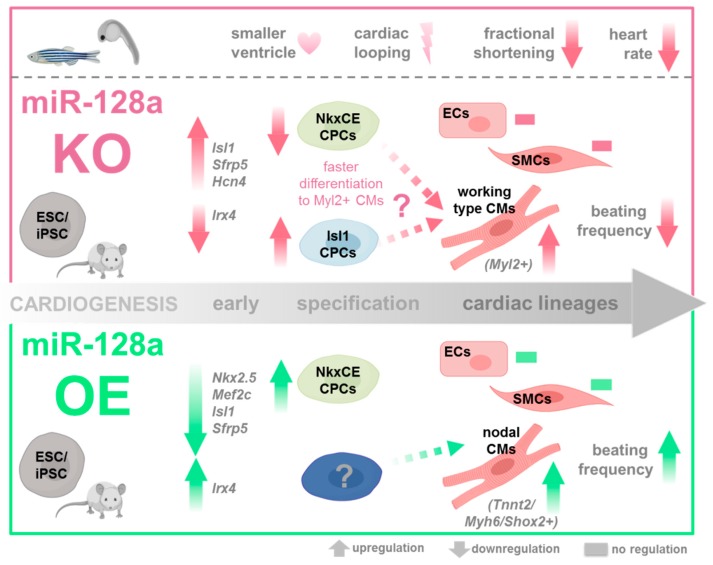
Role of miR-128a in cardiac development. During murine in vitro differentiation, LNA-mediated miR-128a knockdown in differentiating ES/iPSCs (1) increased cardiac transcription factors such as *Isl1*, *Sfrp5*, *and Hcn4* but reduced *Irx4* at the onset of cardiogenesis; (2) upregulated *Isl1*-positive CPCs, whereas NkxCE-GFP-positive CPCs were downregulated; and (3) increased the expression of the ventricular cardiomyocyte marker *Myl2* accompanied by a reduced beating frequency of early cardiomyocytes. Overexpression of miR-128a (4) diminished the expression of *Isl1*, *Sfrp5*, *Nkx2.5*, and *Mef2c*, but increased *Irx4*, (5) enhanced the NkxCE-GFP-positive CPC population, and (6) favored nodal-type-like cardiomyocytes marked by *Tnnt2*, *Myh6,* and *Shox2* accompanied by increased beating rates. Parts of the figure from BioRender.com

Overexpression of miR-128a (4) diminished the expression of *Isl1*, *Sfrp5*, *Nkx2.5*, *Mef2c*, but increased *Irx4*; (5) enhanced NkxCE-GFP-positive CPC abundance, which implicated a retarded differentiation of NkxCE-GFP CPCs referring to the results from miR-128-loss-of-function experiments; and (6) favored nodal-type-like cardiomyocytes marked by *Tnnt2*, *Myh6, and Shox2* accompanied by increased beating frequencies (Figure 6). As already seen in miR-128-knockdown samples, miR-128 overexpression did not alter the expression levels of *Acta2* or *Pecam1,* again indicating that neither smooth muscle cells nor endothelial cells were influenced by miR-128.

The knockdown of miR-128a induced a robust cardiac phenotype in zebrafish larvae accompanied by smaller ventricles, cardiac looping defects, decreased ventricular fractional shortening, and diminished heart rate.

Our studies therefore showed that miR-128a is involved in the regulation of early cardiac progenitor cell populations ((1),(2),(4),(5)) and their differentiation in cardiac lineages, and in the timing of differentiation in various cardiomyocyte subtypes ((3),(6)). It is well known that miR-128 plays a significant role in postnatal cardiomyocytes including a significantly higher expression level in adult mouse cardiomyocytes than in cardiac mouse fibroblasts [12]. This might support our hypothesis that miR-128 also plays a role during differentiation of cardiac progenitor cells in cardiomyocytes. Just recently, it was further demonstrated that *Isl1*, which is targeted by miR-128 [11], directly controlled CPC differentiation, cardiomyocyte identity, and sarcomeric maturation by shaping the chromatin landscape of cardiac progenitor cells [63]. This perfectly fits with our results from miR-128 loss-of-function experiments, leading to more *Isl1*-positive CPCs, increased *Myl2*-positive CMs, and a reduced beating rate (“working-type myocard”).

A recent manuscript postulated an important role for miR-128 during postnatal cardiomyocyte proliferation [12]. However, when we checked for proliferation differences in LNA-128-treated NkxCE-GFP EBs and in LNA-Ctr treated samples, we did not find significant differences for *Ki67* expression, indicating that proliferation is not the mechanism of action for miR-128a during cardiac development. MiR-128 seems to be directly involved in the differentiation process of cardiomyocytes. Our in vitro results proposed a model in which enhanced populations of ventricular, *Myl2*-positive CMs (part of the so-called “working myocard”) were responsible for reduced beating rates of differentiating ESCs upon miR-128 loss-of function, while nodal-like immature cardiomyocytes (e.g., *Tnnt2-*, *Myh6-*, *Shox2*-postitive) exhibit increased firing rates [62] upon miR-128 gain-of-function.

Overall, our in vitro (murine pluripotent stem cells) as well as in vivo (zebrafish) results coincided very well. Atrial myocytes were not affected by miR-128 loss-of-function. Neither were endothelial and smooth muscle cell lineages affected by miR-128 loss- or gain-of function (only in vitro evidence). However, interestingly, zebrafish miR-128 KO larvae showed abnormal cardiac looping and smaller ventricles. This misdirected cardiogenesis was mirrored in vitro by differentially regulated expression levels of important cardiac transcription factors (*Isl1*, *Sfrp5*, *Nkx2.5*, *Mef2c*, *Irx4*, *Hcn4*) and changes in the frequency of *Isl1*-positive as well as NkxCE-GFP-positive CPCs. Functional cardiac parameters including reduced heart rate in miR-128 knock-down zebrafish larvae also closely resembled our results obtained from in vitro studies where we observed a reduced beating frequency in early differentiating cardiomyocytes upon miR-128 KO and an increased beating frequency after miR-128 overexpression.

## 4. Materials and Methods

### 4.1. Transgenic Animals

The *Nkx2.5* cardiac enhancer eGFP (NkxCE GFP) reporter mouse exclusively marks cardiac progenitor cells (CPCs) in the embryonic heart at E8.5 - E15.5 as described previously [8] (Figure 1A,B). Transgenic mice were housed in an accredited facility in compliance with the European Community Directive related to laboratory animal protection (Directive 2010/63/EU). For extraction of embryos, tissue or organs mice were anesthetized at the respective timepoint with isoflurane (2-chloro-2-(difluoromethoxy)-1,1,1-trifluoro-ethane; Baxter, Deerfield, IL, USA) and then euthanized by cervical dislocation.

Transgenic zebrafish *Tg(my/7:ras-GFP)* and wild-type *AB* and *TL* were used for miR knockdown *in vivo* studies [23]. Zebrafish were raised and maintained in E3 medium (5 mM NaCl, 0.17 mM KCl, 0.33 mM CaCl_2_, 0.33 mM MgSO_4_, (Carl Roth, Karlsruhe, Germany) 10^−5^% Methylene Blue (Sigma Aldrich, St Louis, MO, USA) at 28 °C in zebrafish housing systems with a light/dark hour cycle of 14/10. Embryos for microinjections were obtained by mating the lines mentioned above. All zebrafish work followed Institutional Animal Care and Use Committee-approved protocols.

All experiments performed with mice or zebrafish (i) were approved by the local legislation on protection of animals (Commission on Animal Protection, Regierung von Oberbayern [Regional Government of Upper Bavaria; based on Section 4, paragraph 3, Animal Protection Act] or Regierungspräsidium Karlsruhe [Karlsruhe Regional Council, based on Section 4, paragraph 3, Animal Protection Act]). (ii) All animal experiments (organ or embryo extractions) were performed in accordance with the European guidelines and regulations for animal care and handling (Directive 2010/63/EU).

### 4.2. Human Induced Pluripotent Stem Cells

Human induced pluripotent stem cells (hiPSCs) were generated from peripheral blood mononuclear cells (PBMCs) of a healthy individuum (34 years, male, informed consent was obtained) by reprogramming with the four Yamanaka factors (OCT4, c-MYC, SOX2, KLF4) delivered by non-integrating sendai viruses (Invitrogen, Carlsbad, CA, USA). HiPSCs were characterized at the German Heart Center Munich, Department of Cardiovascular Surgery, Institute Insure (unpublished data). All experiments (i) were performed under the approval of the local ethical committee of the Technische Universität München [Munich Technical University] (KaBi/DHM 5943/13). (ii) All experimental procedures were performed in accordance with the principles outlined in the Declaration of Helsinki.

Please find the complete methods section in the Supplementary material.

## 5. Conclusions

In summary, our study demonstrated that miR-128a plays a so-far unknown role in early heart development. MiR-128a seemed to be involved in CPC differentiation, and in the timing of differentiation into different cardiomyocyte subtypes. Knockout in zebrafish caused cardiac malformations including reduced heart rates and smaller ventricles. While important progress was made in identifying the roles of miR-128a in physiological and pathophysiological processes of the postnatal heart, our results shed light on the regulatory functions of miR-128a during early cardiac development. Therefore, our results contribute to a more complete picture of miR-mediated regulations during cardiogenesis.

## Supplementary Materials

Supplementary Materials can be found at https://www.mdpi.com/1422-0067/21/3/1158/s1.

## Abbreviations

bpm	beats per minute
CFs	cardiac fibroblasts
CMs	cardiomyocytes
CPC	cardiac progenitor cells
Ctr	control
D	day(s)
dox	doxycycline
E8.5	embryonic day 8.5
EB	embryoid bodies
ECs	endothelial cells
ESCs	embryonic stem cells
FACS	Fluorescence activated cell sorting (flow cytometry)
GFP	green fluorescent protein
GO	gene ontology
GSEA	gene set enrichment analysis
hiPSCs	human induced pluripotent stem cells
hpf	hours post fertilization
iITG-iPSCs	Isl1-Cre/ROSA26^mTmG^ induced pluripotent stem cells
ITG	Isl1-Cre/Rosa26^mT/mG^
KO	knockdown
LNA	Locked nucleic acid
mG	membrane-tagged GFP
miRs	microRNAs
MO	morpholino-modified oligonucleotides
mRNA	messenger RNA
mT	membrane-tagged tdTomato
MTT	3-[4.5-Dimethylthiazol-2-yl]-2.5-di phenyltetrazolium bromide
NkxCE	Nkx2.5 cardiac enhancer
NkxCE-CPCs	Nkx2.5 cardiac enhancer cardiac progenitor cells
NkxCE-GFP	Nkx2.5 cardiac enhancer GFP
OE	overexpression
P	postnatal day
PBMCs	peripheral blood mononuclear cells
qRT-PCR	Semiquantitative real time polymerase chain reaction
RNA	ribonucleic acid
RNAseq	RNA sequencing
SAN	sinoatrial node
SEM	standard error mean
SMCs	smooth muscle cells
tet-on	doxycycline-inducible
tRFP	Turbo red fluorescent protein
TTFs	tail tip fibroblasts
UTR	untranslated regions
w/o	without
wk(s)	week(s)
