# Supplementary material for: miR-128a Acts as a Regulator in Cardiac Development by Modulating Differentiation of Cardiac Progenitor Cell Populations
Source: Int J Mol Sci. 2020 Feb 10;21(3):1158. doi: 10.3390/ijms21031158 (PMC7038042; doi:10.3390/ijms21031158)
Supplement: Supplementary file 1 [file ijms-21-01158-s001.zip › Hoelscher et al_Suppl_2020_01_11SD_incl.Figures,Tables_FINAL.pdf]

# Supplementary Information

## miR-128a acts as a regulator in early cardiac development by modulating differentiation of cardiac progenitor cell populations

Sarah C. Hoelscher[1,2,3], Theresia Stich[1,2], Anne Diehm[1,2], Harald Lahm[1,2], Martina Dreßen[1,2], Zhong Zhang[1,2], Irina Neb[1,2], Zouhair Aherrahrou[4,5,6], Jeanette Erdmann[4,5,6], Heribert Schunkert[3,7], Gianluca Santamaria[8], Giovanni Cuda[9], Ralf Gilsbach[10], Lutz Hein[10,11], Rüdiger Lange[1,2,3], David Hassel[12,13], Markus Krane*[1,2,3] Stefanie A. Doppler*[1,2]

1. Supplementary Figures and Supplementary Figure Legends
2. Supplementary Video Legends
3. Supplementary Tables
4. Supplementary Methods
5. Supplementary References

# 1. Supplementary Figures and Figure Legends

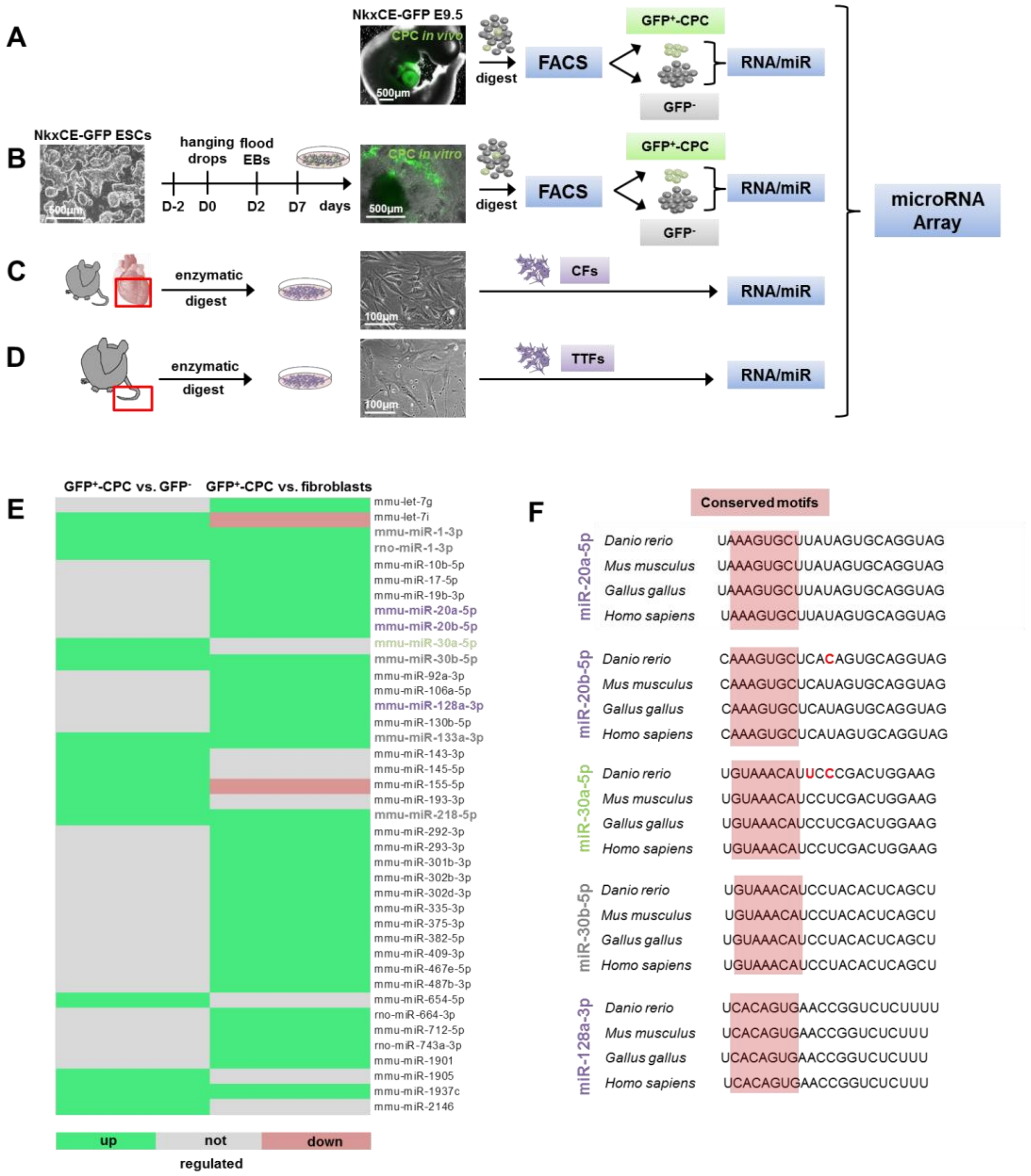


**Suppl. Figure S1. Identification of candidate miRs during cardiac development.**

**A.-D.** Generation of cell samples for microRNA Array. E9.5 Nkx2.5 cardiac enhancer eGFP (NkxCE-GFP) embryos were collected and enzymatically digested. GFP-positive CPCs and the correspondent GFP-negative fractions were subsequently sorted using flow cytometry (FACS) (A). Differentiated (hanging drop method) NkxCE-GFP ESCs were harvested and enzymatically digested after 7 days (D7), FACS sorted and RNA/miR was isolated of GFP$^{+}$ and GFP$^{-}$ cells. (B). Cardiac fibroblasts (CFs) (C) and tail tip fibroblasts (TTFs) (D) were *in vitro* cultivated after enzymatic digestion from hearts or tailtips of adult NkxCE-GFP mice. RNA/miR was isolated from low passage cells (C). Isolated miR was then used for microRNA Arrays. **E.** Heat map of 41 upregulated miRs ($p < 0.05$) of the microRNA Array. All together

about 800 miRs were evaluated. **F**. Sequences of miR-20a-5p, miR-20b-5p, miR-30a-5p, miR-30b-5p and miR-128a-3p are highly conserved between species, including zebrafish (*danio rerio*), mouse (*mus musculus*), chicken (*gallus gallus*) and human (*homo sapiens*). Red bases represent non-homologous bases compared to human miR-sequences.

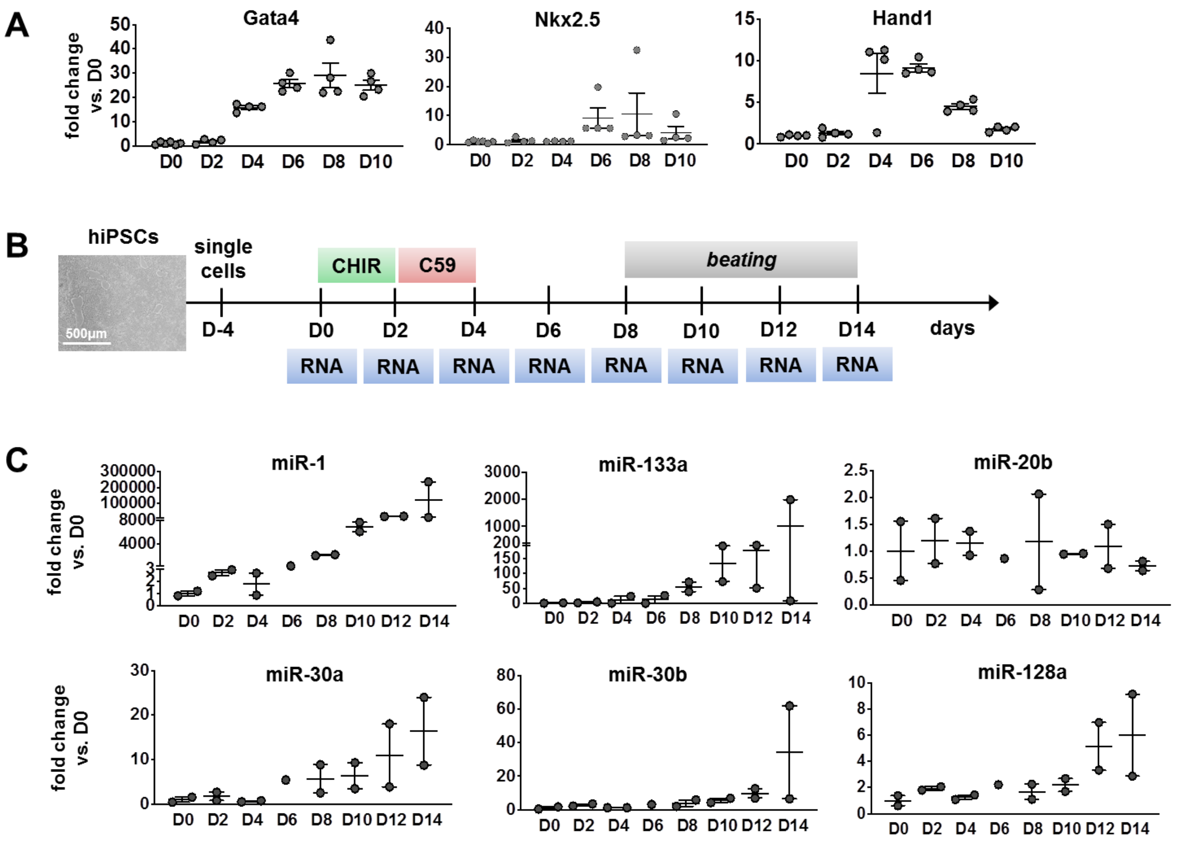


## Suppl. Figure S2: Identification of candidate miRs during cardiac development.

**A.** Expression kinetics of *Gata4*, *Nkx2.5* and *Hand1* (early cardiac transcription factors) during *in vitro* differentiation of NkxCE-GFP ESCs. Gene expression starts to elevate on day 4 (D4) to 6 (D6). **B.** huiPSCs were *in vitro* differentiated for two weeks. Wnt-signaling was activated from day 0 (D0) until day two (D2) using CHIR99021 (GSK3 inhibitor) followed by a treatment with Wnt-C59 (Wnt-signaling inhibitor) for two days. Total RNA was isolated every other day to evaluate microRNA kinetics. **C.** Kinetics of miR candidates during *in vitro* differentiation of huiPS. Expressions of miR-1 and -133a followed a typical course by rising upon the beginning of cardiomyogenesis (around day 8-10). MiR-30a, -30b and -128a also elevated around day 8 (D8) to 10 (D10). However, miR-20b showed no recognizable trend of upregulation during differentiation.

Data are represented as means ± SEM.

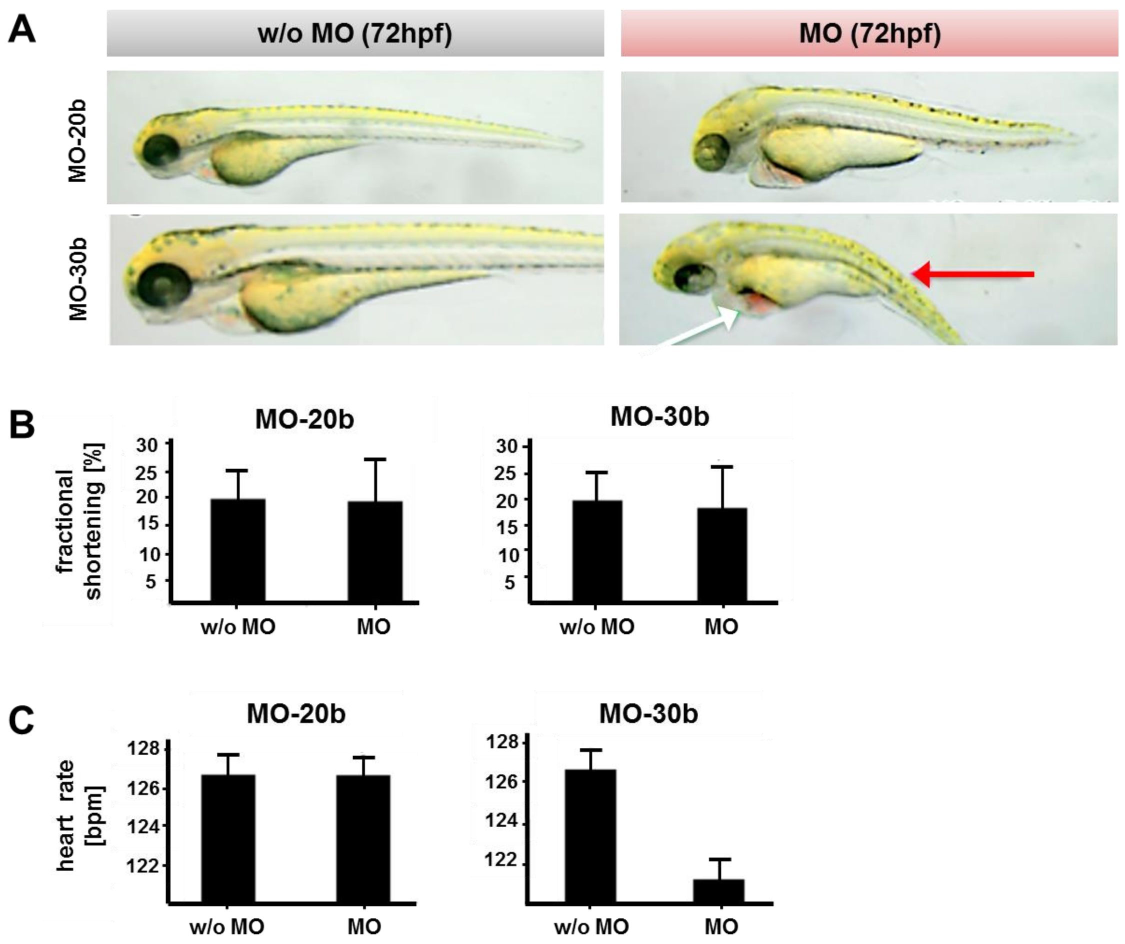


**Suppl. Figure S3: Evaluation of candidate miR function during zebrafish development.**

**A.** Morphological changes of morpholino oligo (MO)-treated zebrafish larvae at 72 hpf. MO-20b morphants showed decreased body size compared to controls (w/o MO) (upper panels). MO-30b morphants exhibited extreme blood stasis (white arrow) and crimped tails (red arrow) (lower panels). **B.** MO-20b and MO-30b treated larvae showed no difference in fractional shortening compared to control larvae (w/o MO) at 72hpf. **C.** Knockdown of miR-20b or -30b had no significant effects on heart rate in zebrafish morphants at 72 hpf compared to controls (w/o MO).

Data are represented as means ± SEM.

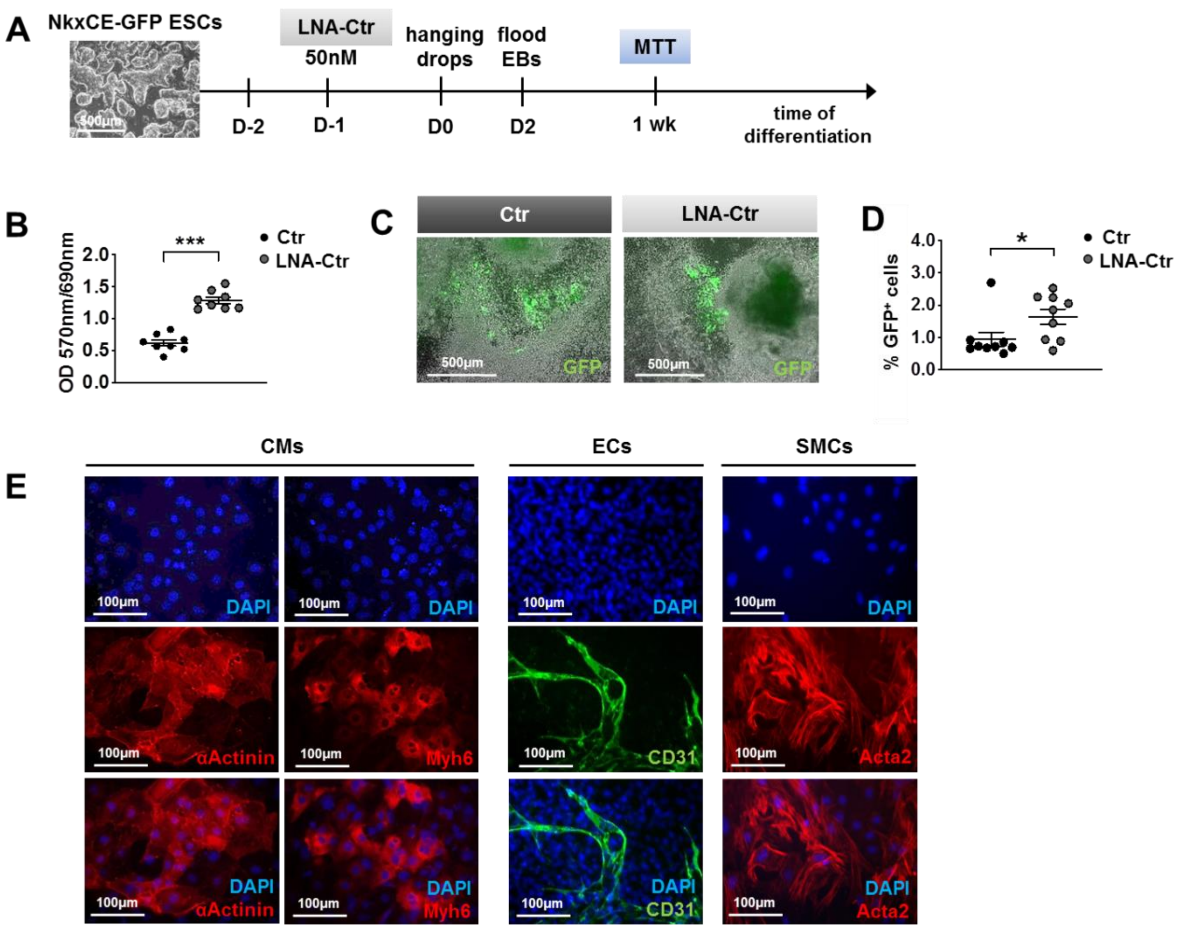


**Suppl. Figure S4: LNA probes during *in vitro* differentiation of murine NkxCE-GFP ES cells.**

**A.** NkxCE-GFP ESCs were differentiated using the hanging drop method and transfected with 50nM LNA-Ctr probes at day-1 (D-1). After one week, (wk) MTT assay was performed and compared to non-transfected, equally differentiated NkCE-GFP ESCs (Ctr). **B.** MTT Assay: After one week of differentiation NkxCE-GFP ESCs transfected with LNA-Ctr probes showed a significant upregulation of proliferation capacity ($p < 0.001$) when compared to non-tranfected NkxCE-GFP ESCs. **C.** GFP-positive NkxCE CPCs after one week of differentiation in non-transfected NkxCE-GFP embryoid bodies (EBs) and LNA-Ctr-transfected NkxCE-GFP EBs. Images are an overlay between phase contrast and fluorescent microscopic pictures. **D.** Frequency of GFP-positive NkxCE CPCs after one week of differentiation was significantly enhanced in LNA-Ctr-transfected NkxCE-GFP EBs compared to non-transfected NkxCE-GFP EBs. **E.** Immunofluorescent stainings showed that after two to three weeks of differentiation NkxCE-GFP ESCs differentiated into the three major cardiac cell types including cardiomyocytes (α-Actinin, α-Myosin Heavy Chain (Myh6)), endothelial cells (CD31), and smooth muscle cells (Smooth Muscle Actin (Acta2)).

Data are represented as means ± SEM. * $p \leq 0.05$, *** $p \leq 0.001$.

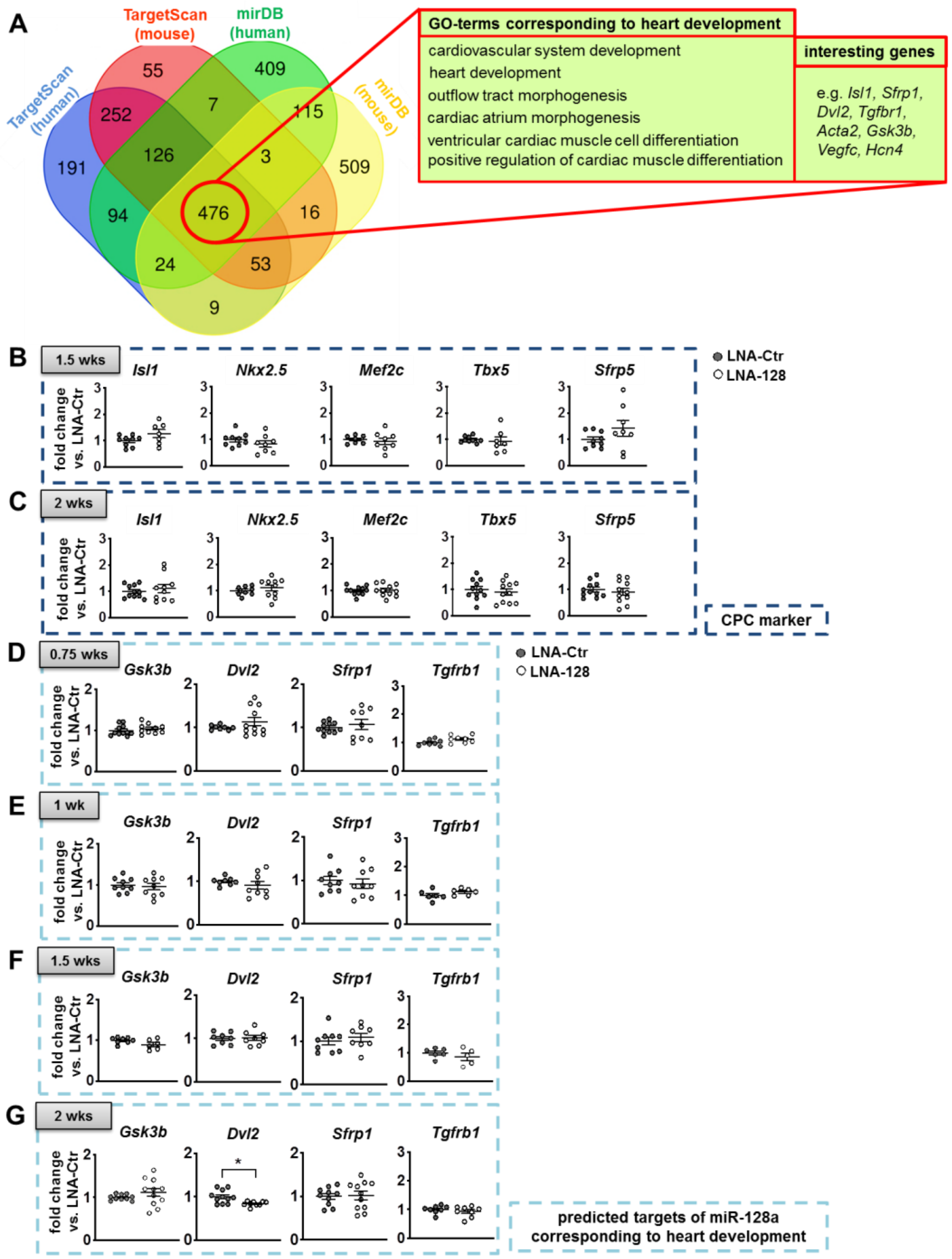


**Suppl. Figure S5: Predicted target genes of miR-128a and gene expression data after knockdown of miR-128a during *in vitro* differentiation of murine NkxCE-GFP ESCs.**

**A.** Venn diagram analysis of predicted miR-128a target genes (human and murine) from Target Scan version 7.2 (http://www.targetscan.org) and miRDB (http://mirdb.org). 476 genes were found to be included in all four lists. **B-C.** Panels show the expression of early CPC markers after 1.5 weeks (wks) (B.) and after 2 wks (C.). **D-G.** The expression of *Gsk3b*, *Dvl2* and *Sfrp1* (Wnt signaling) as well as *Tgfbr1* was not influenced (0.75-2 wks) by a knockdown of miR128-a during the differentiation of NkxCE-GFP-ESCs.

Data are represented as means ± SEM. * $p \leq 0.05$.

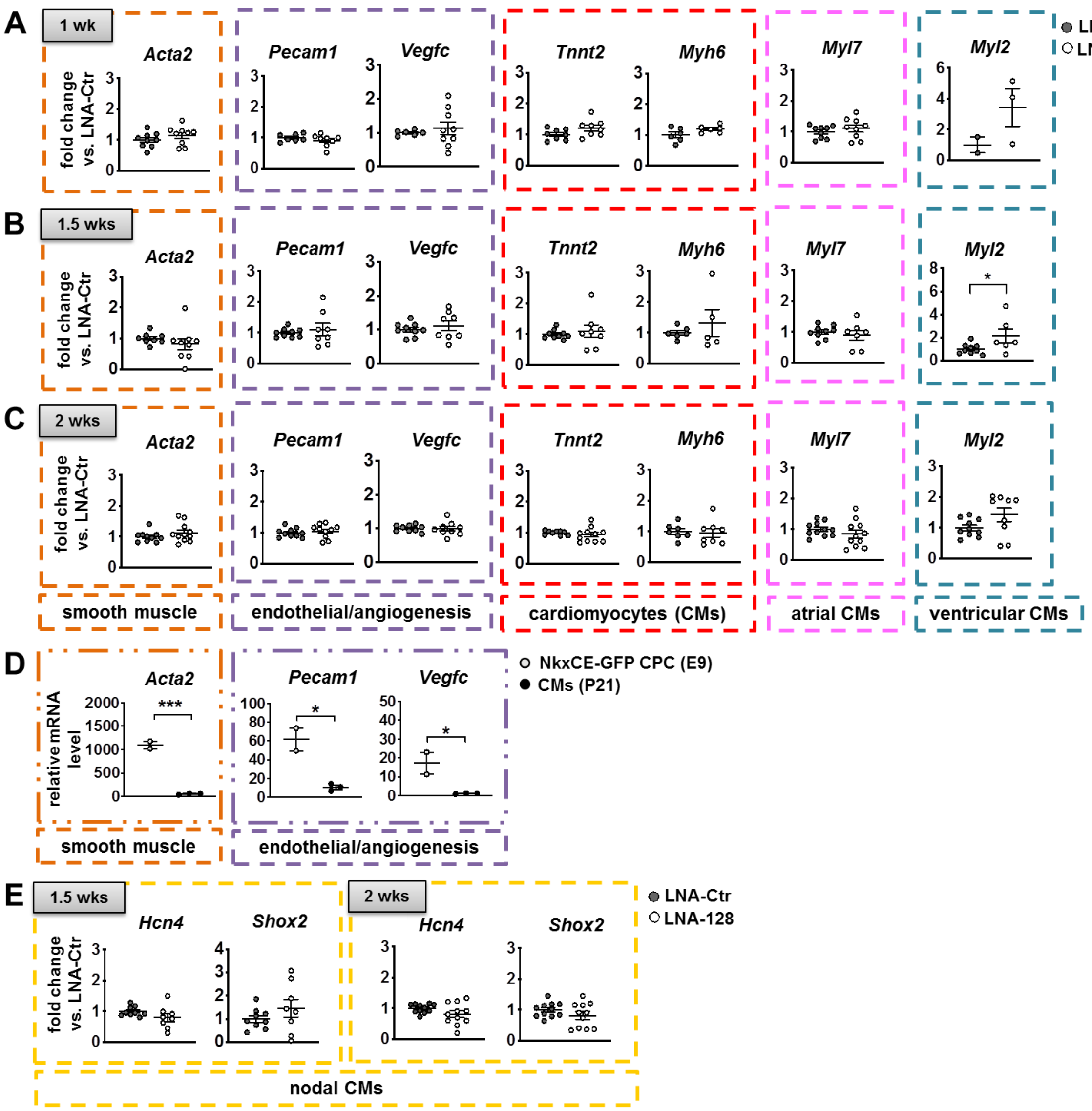


**Suppl. Figure S6: Gene expression data after knockdown of miR-128a during *in vitro* differentiation of murine NkxCE-GFP ESCs.**

**A-C.** The expression of *Acta2* (smooth muscle cell marker), *Pecam 1* (endothelial cell marker), *Vegfc* (angiogenesis marker) as well as general cardiomyocyte (CM) marker (*Tnnt2, Myh6*), atrial CM (*Myl7*) and ventricular CM marker (*Myl2*) are shown from 1 wk to 2 wks. **D.** RNA sequencing data depict that NkxCE-GFP-positive cells from embryonic hearts (embryonic day 9, E9) expressed high levels of *Acta2, Pecam1* and *Vegfc* compared to cardiomyocytes from adult mouse hearts (postnatal day 21, P21). **G.** Nodal CM markers (*Hcn4, Shox2*) were not differentially regulated during differentiation of NkxCE-GFP ESCs transfected with LNA-128 or LNA-Ctr probes (panels after 1.5 and 2 wks are shown).

Data are represented as means ± SEM. * $p \leq 0.05$, *** $p \leq 0.001$.

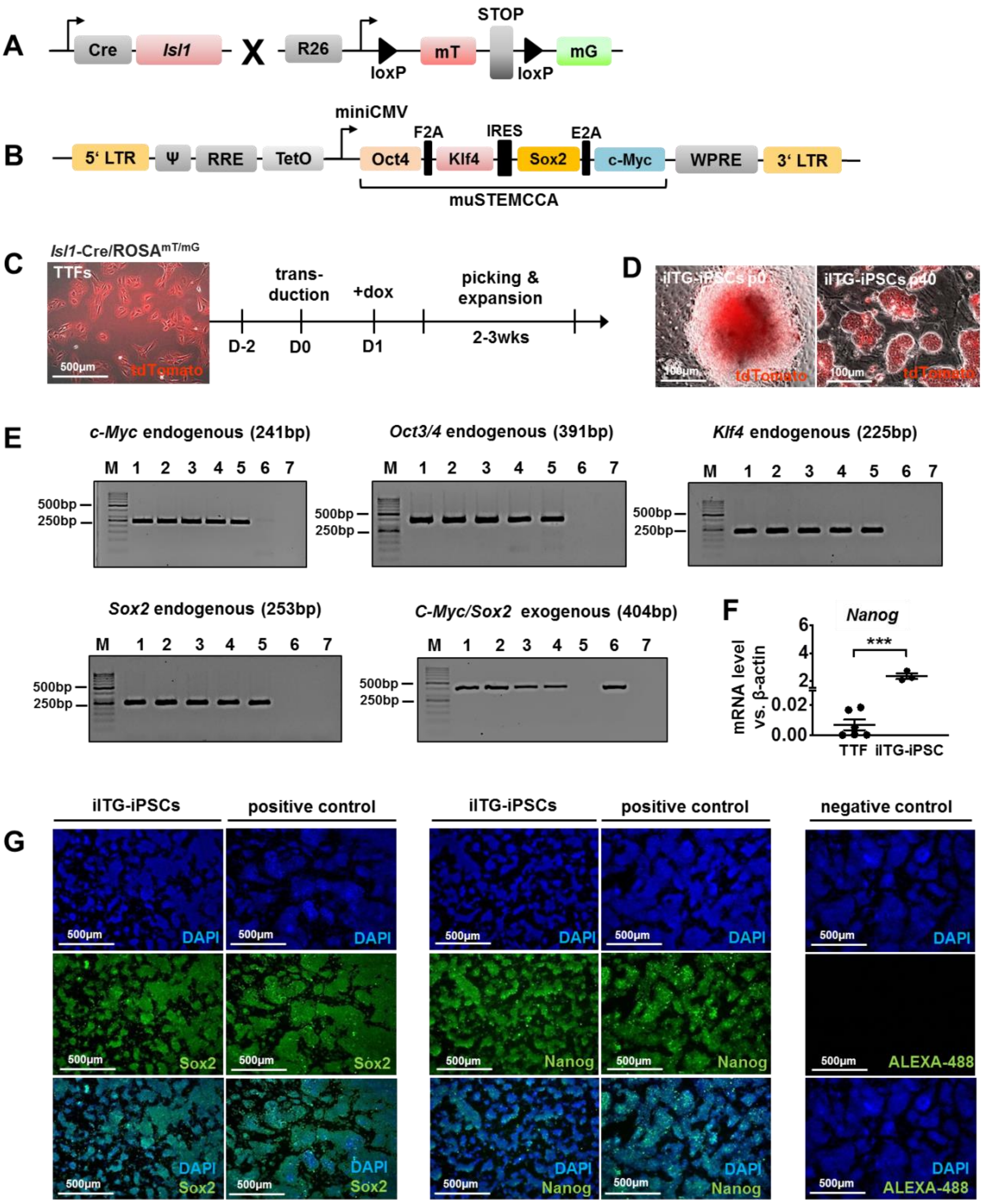


**Suppl. Figure S7: Generation and verification of murine Isl1-Cre/Rosa26$^{mTmG}$ iPSCs (iITG-iPSCs).**

**A.** Isl1-Cre/Rosa26$^{mTmG}$ (ITG) transgenic construct: ubiquitous expression of dtTomato and labeling of Isl1 expressing cells by GFP after Cre-mediated excision of dtTomato. **B.** Doxycycline(dox)-inducible polycistronic lentiviral vector for reprogramming of transgenic ITG fibroblasts into iPSCs (iITG-iPSCs). It contains the four “Yamanaka factors” Oct4, Klf4, Sox2 and c-Myc (OKSM, “stem cell cassette”; murine STEMCCA) separated by self-cleaving 2A peptides and IRES (internal ribosomal entry site) sequences. *Abbreviations: LTR, long terminal repeats; RRE, Reverse Response Element; TetO, tetracycline operator, CMV,*

*Cytomegalovirus Promoter; WPRE, Woodchuck Hepatitis Posttranscriptional Regulatory Element*. **C.** Isl1-Cre/Rosa26$^{mTmG}$ tail tip fibroblasts (ITG-TTFs) were transduced with lentiviral dox-inducible muSTEMCCA particles and lentiviral particles containing a reverse tetracyclin transactivator (rtTA). Expression of the "Yamanaka factors" was induced one day (D1) after transduction. IPSC colonies were picked two to three weeks after transduction and further expanded. **D.** Microscopic images of an iPSC colony before picking and expanded iPSCs after 40 passages. Images are an overlay between phase contrast and fluorescent microscopic pictures. **E.** Different passages of generated iITG-iPSCs endogenously expressed all four "Yamanaka factors" (c-Myc, Oct3/4, Klf4 and Sox2) at similar levels than ESCs (NkxCE-GFP ESCs) (Block Cycler PCRs). With increasing passages, generated iPSCs showed declining expression of exogenous c-Myc/Sox2 (polycistronic vector construct). *(M) marker; (1) iITG-iPSC p1; (2) iITG-iPSC p3; (3) iITG-iPSC p13*; *(4) iITG-iPSC p18; (5) NkxCE-GFP ESCs p28 (control 1); (6) muSTEMCCA (control 2); (7) $H_2O$ (control 3).* **F.** Generated iPSCs expressed significant higher levels of the pluripotency marker *Nanog* when compared to tail tip fibroblasts (qRT-PCR). **G.** iITG-iPSCs expressed the pluripotency markers Sox2 (left panel) and Nanog (right panel) like ESCs (NkxCE-GFP ESCs) (immunofluorescent stainings with anti-Sox2 and anti-Nanog antibodies).

Data are represented as means ± SEM. *** $p \leq 0.001$.

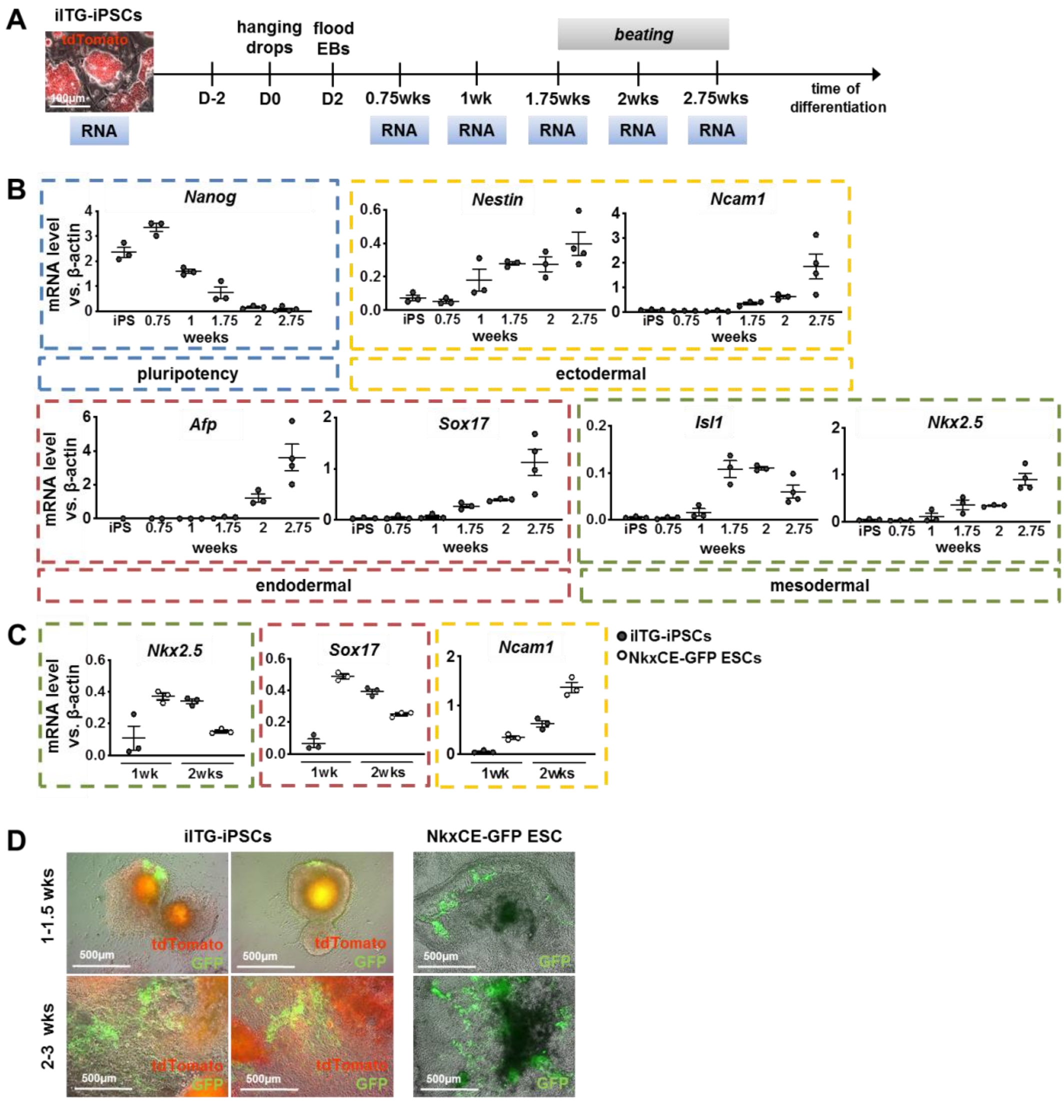


**Suppl. Figure S8: *In vitro* differentiation of murine Isl1-Cre/Rosa26$^{mTmG}$ iPSCs (iITG-iPSCs) for verification of differentiation capability into all three germ layers.**

**A.** iITG iPSCs were differentiated using the hanging drop method. RNA for qRT-PCR analysis was isolated at respective timepoints. Differentiated iPSCs started beating after 1.75 to 2 weeks (wks) of differentiation. **B.** Kinetics of pluripotency and germ layer markers during iITG iPSC *in vitro* differentiation. The expression of pluripotency marker *Nanog* decreased throughout differentiation whereas the expression of all three germ layer markers rose (ectodermal: *Nestin, Ncam1*, endodermal: *Afp, Sox17*, mesodermal: *Isl1, Nkx2.5*). **C.** Throughout *in vitro* differentiation, the generated iITG-iPSCs started to express meso-, endo- and ectodermal markers approximately one week later than ESCs (NkxCE-GFP ESCs). **D.** Microscopic images of Isl1-GFP-positive CPCs of iITG-iPSCs differentiated for 1-1.5 wks (upper left panels) or 2-3 wks (lower left panels). Timepoint-matched NkxCE-GFP-positive CPCs as comparison (right panels). Images are an overlay between phase contrast and fluorescent microscopic pictures. Images show that iITG-iPSCs differentiated slower than corresponding NkxCE ESCs.

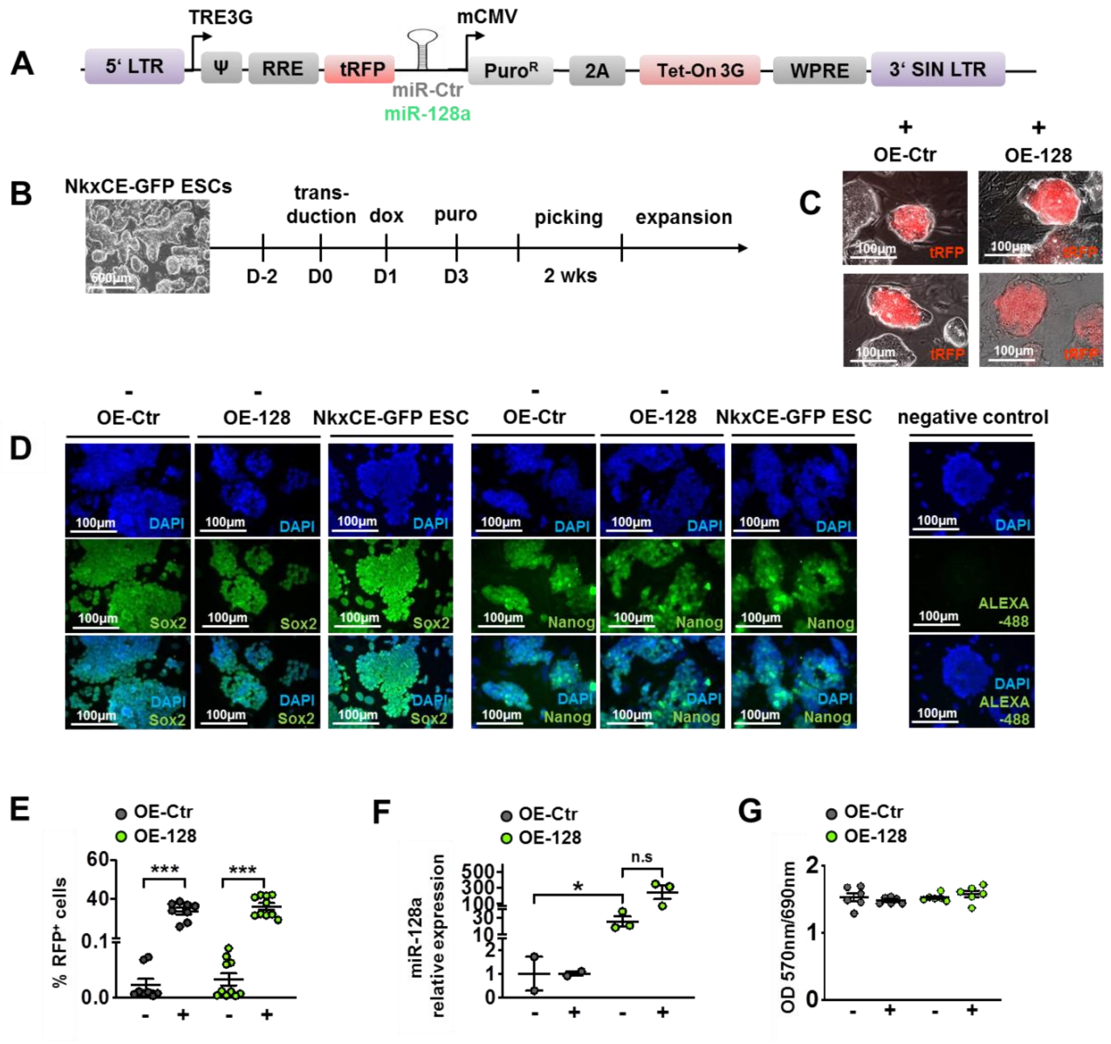


**Suppl. Figure S9: Generation and verification of miR overexpressing murine NkxCE-GFP ESCs (OE-128, OE-Ctr).**

**A.** Lentiviral miR overexpression (OE) vector for transduction of NkxCE-GFP ESCs: the vector is doxycycline inducible (tet-on 3G), carries an ubiquitous puromycine resistence (Puro$^R$) and turbo Red Fluorescent Protein (tRFP) is expressed in front of the respective miR to be overexpressed, either miR-128a (OE-128) or a non-targeting control miR (miR-Ctr, OE-Ctr). *Abbreviations: LTR, long terminal repeats; TRE3G, Tetracycline Response Element 3G; RRE, Reverse Response Element; mCMV, murine Cytomegalovirus Promoter; 2A, 2A peptide; WPRE, Woodchuck Hepatitis Posttranscriptional Regulatory Element; SIN LTR, Self-inactivating Long Terminal Repeats*. **B.** NkxCE ESCs were transduced with either OE-Ctr or OE-128 lentiviral particles (day 0, D0). tRFP and miR expression was induced at D1 by adding doxycycline (dox) and transduced ESCs were selected by Puro from day D3. RFP-positive clones were picked after one to two weeks of selection and further expanded**. C.** Microscopic images of RFP-positive OE-Ctr and OE-128 colonies before picking. Images are an overlay between phase contrast and fluorescent microscopic pictures. **D.** OE-Ctr as well as OE-128 ESCs expressed the pluripotency markers Sox2 (left panel) and Nanog (right

panel) like the originating ESCs (NkxCE-GFP ESCs) (immunofluorescent stainings with anti-Sox2 and anti-Nanog antibodies). Experiments were performed without dox-induction of miRs. **E.** Doxycycline (+) significantly induced tRFP in OE-Ctr and OE-128 ESCs (p<0.001) compared to their counterparts without doxycycline (-) (% RFP+ cells was evaluated by flow cytometry). **F.** No miR-128a expression was induced upon dox-addition in OE-Ctr ESCs. However, OE-128 ESCs showed a significantly elevated miR128a-expression level even without dox compared to OE-Ctr ESCs. Unfortunately, by adding dox (2µg/ml) to OE-128 ESCs no further upregulation of miR128a could be achieved. **G.** MTT Assay: No significant differences concerning proliferation capacity were observed in OE-Ctr and OE-128 ESCs cultivated with or without dox.

Data are represented as means ± SEM. * p ≤ 0.05, ** p ≤ 0.01 and *** p ≤ 0.001.

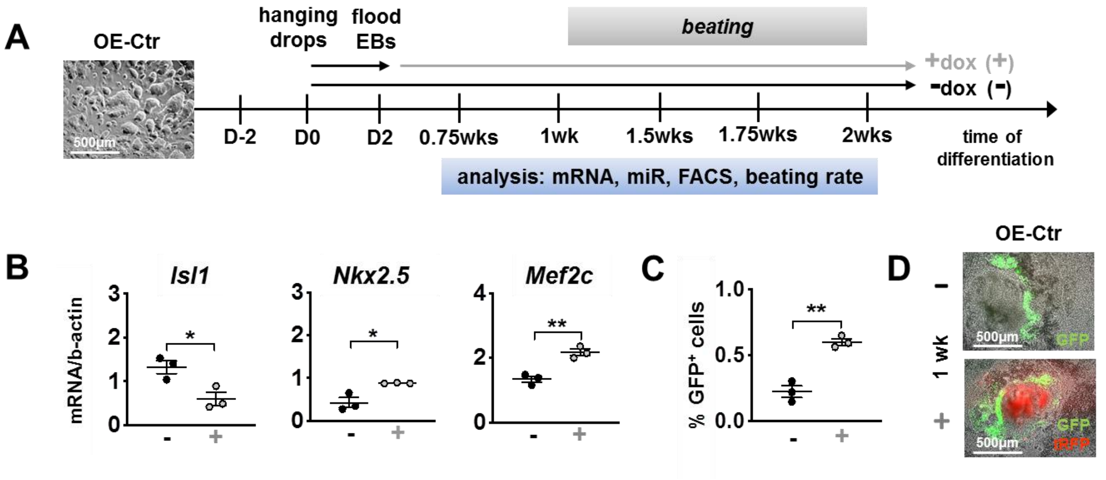


**Suppl. Figure S10:** ***In vitro*** **differentiation of murine OE-Ctr NkxCE-GFP ESCs (OE-Ctr) to evaluate doxycycline related effects.**

**A.** OE-Ctr ESCs were differentiated using the hanging drop method for two weeks (wks) either with (+dox,+) or without (-dox,-) doxycycline. Analysis was performed on respective timepoints between 0.75 and 2 wks of differentiation. *Abbreviations: EB: embryoid bodies; FACS: flow cytometry*; **B.** Gene expression of early CPC markers *Isl1*, *Nkx2.5* and *Mef2c* was significantly different between OE-Ctr EBs with (+) or without (-) dox after 1.5 to 2 wks of differentiation. **C.** Frequency of NkxCE-GFP-positive CPCs was significantly upregulated upon dox-addition (+) in OE-Ctr EBs compared to OE-Ctr EBs without dox (-) after 2 wks of differentiation. **D.** Upon dox-addition (+) (lower panel) more NkxCE-GFP-positive CPCs developped in OE-Ctr EBs compared to OE-Ctr EBs without dox (-) (upper panel) after 1 wk of differentiation. Images are an overlay between phase contrast and fluorescent microscopic pictures.

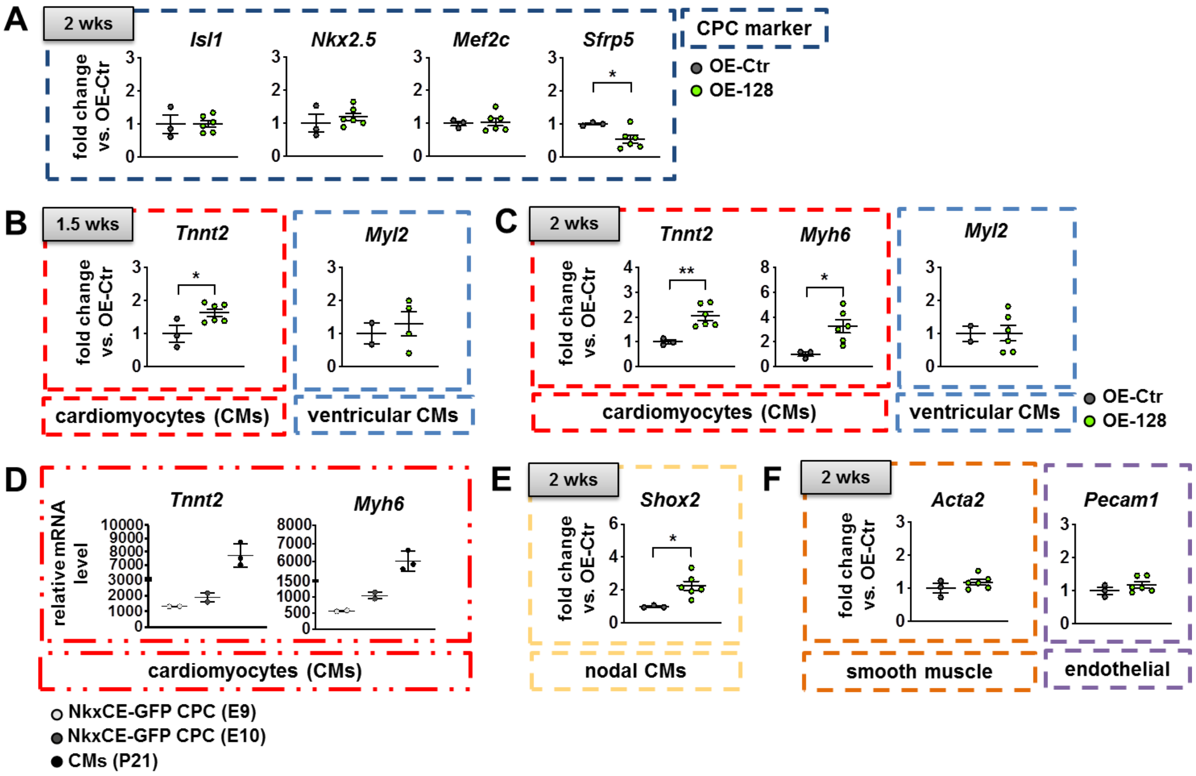


**Suppl. Figure S11: Gene expression data after overexpression of miR-128a during *in vitro* differentiation of murine OE-128 and OE-Ctr NkxCE-GFP ESCs.**

**A.** Panels show the expression of early CPC markers (*Isl1, Nkx2.5, Mef2c, Sfrp5*) after 2 weeks (wks) of differentiation. **B.-C.** General cardiomyocyte (CM) markers (*Tnnt2, Myh6*), and ventricular CM marker *Myl2* are shown from 1.5 to 2 wks. **D.** RNA sequencing data depict that NkxCE-GFP-positive cells from embryonic hearts (embryonic day 9 and 11, E9 and E11) already expressed high levels of *Tnnt2* and *Myh6* whereas not as high as cardiomyocytes from adult mouse hearts (postnatal day 21, P21). **E.** Nodal CM marker *Shox2* was upregulated upon miR-128a OE after 2 wks. **F.** *Acta2* (smooth muscle cell marker) and *Pecam 1* (endothelial cell marker) were not differentially regulated after 2 wks.

Data are represented as means ± SEM. * $p \leq 0.05$.

## 2. Supplementary Video Legends

Videos are not real-time but assembled from single pictures photographed in a time series. Therefore beating frequency is an artifact of exposure time. Each video displays 70-80 images. Magnification is 100x.

**Video V1-V3. *In vitro* differentiation of *Nkx*CE-GFP ESCs treated with LNA-probes (LNACtr or LNA128) from day 3.5 led to GFP-positive beating areas similar to *Nkx*CE-GFP ESCs not treated with LNA-probes (w/o LNA). V1.** Time-lapse imaging of *Nkx*CE-GFP EBs not treated with LNA probes after one week of *in vitro* differentiation. **V2.** Time-lapse imaging of *Nkx*CE-GFP EBs treated with LNACtr probes after one week of *in vitro* differentiation. **V3.** Time-lapse imaging of *Nkx*CE-GFP EBs treated with LNA128 probes after one week of *in vitro* differentiation.

**Video V4-V5. *In vitro* differentiation of iITG-iPSCs treated with LNA-probes (LNACtr or LNA128) from day 5 led to GFP-positive beating areas. V4.** Time-lapse imaging of iITG-iPSC EBs treated with LNACtr probes after two weeks of *in vitro* differentiation. **V5.** Time-lapse imaging of iITG-iPSC EBs treated with LNA128 probes after two weeks of *in vitro* differentiation.

# 3. Supplementary Tables

**Supplementary Table S1**: Sequences of microRNA Primers for qRT-PCR

| microRNA Primer | Target sequence (5'- 3') | Accession (Exiqon) |
|---|---|---|
| ***hsa-miR-128-3p LNA™ PCR primer set, UniRT*** | UCACAGUGAACCGGUCUCUUU | MIMAT0000424 |
| ***hsa-miR-1 LNA™ PCR primer set, UniRT*** | UGGAAUGUAAAGAAGUAUGUAU | MIMAT0000416 |
| ***hsa-miR-133a-5p LNA™ PCR primer set, UniRT*** | AGCUGGUAAAAUGGAACCAAAU | MIMAT0026478 |
| **hsa-miR-20b-5p *LNA™ PCR primer set, UniRT*** | CAAAGUGCUCAUAGUGCAGGUAG | MIMAT0001413 |
| **hsa-miR-30b-5p *LNA™ PCR primer set, UniRT*** | UGUAAACAUCCUACACUCAGCU | MIMAT0000420 |
| **hsa-miR-30a-5p *LNA™ PCR primer set, UniRT*** | UGUAAACAUCCUCGACUGGAAG | MIMAT0000087 |
| ***U6 snRNA LNA™ PCR primer set, UniRT*** **(reference RNA)** | GUGCUCGCUUCGGCAGCACAUAU ACUAAAAUUGGAACGAUACAGAG AAGAUUAGCAUGGCCCCUGCGCA AGGAUGACACGCAAAUUCGUGAA GCGUUCCAUAUUUUU | without MIMAT # |

**Supplementary Table S2**: Predicted target genes of miR-128a (hsa-128-3p, mmu-128-3p) by TargetScan (http://www.targetscan.org) or miRDB (http://mirdb.org).

| TargetScan | **TargetScan** | **miRDB** | **miRDB** |
|---|---|---|---|
| *human* | *mouse* | *human* | *mouse* |
| **1225 genes** | **988 genes** | **1254 genes** | **1205 genes** |
| SZRD1 | SZRD1 | AFF4 | Szrd1 |
| PAIP2 | NOVA1 | SZRD1 | Anks1 |
| NOVA1 | LBH | KDM7A | Plk2 |
| PRKX | PRKD1 | SEC22A | Erlec1 |
| PRKD1 | HIPK2 | GPAM | Ret |
| CLPP | AGRN | FRMPD3 | Mapk14 |
| DUSP18 | PDIA5 | RPS6KA5 | Dcun1d4 |
| PDIA5 | PLK2 | PAIP2 | Ube2e2 |
| PLK2 | PLAGL1 | PHF6 | Zfp26 |
| STAB2 | APEX1 | DCUN1D4 | Ugt8a |
| SEC22A | TMSB10 | SMAP1 | Reln |
| CASC3 | ANK2 | IGLON5 | Diras1 |
| GPATCH2L | RAB20 | UBE2E2 | Poglut1 |
| UBE2E2 | PPP1CC | KCNK2 | Dot1l |
| TMSB10 | MAPK14 | POGLUT1 | Arhgap12 |
| YWHAB | YWHAB | ARRDC4 | Nfx1 |
| TMEM167A | MIPOL1 | MOSMO | Kctd11 |
| VWC2L | POGLUT1 | KCNK10 | Aff4 |

| | | | |
|---|---|---|---|
| DCUN1D4 | TRIM23 | CDS1 | Ece2 |
| AK2 | DCUN1D4 | PLK2 | Matn3 |
| APEX1 | GOLM1 | TNPO1 | Eef1akmt4-ece2 |
| LBH | RET | TRPV3 | Mosmo |
| ST6GALNAC3 | SH3BGRL2 | TMTC2 | Zfp652 |
| H3F3C | FAM184A | GRIA3 | Ubr5 |
| BAG2 | YPEL3 | SRGAP2 | B3gnt7 |
| PITHD1 | GATAD2A | OTULIN | Cds1 |
| MIPOL1 | C16orf80 | MSI2 | Arid1b |
| VEGFC | GLTP | DTX4 | Phf6 |
| SFXN2 | CSDC2 | CSF1 | Ttc9 |
| SNX12 | POGZ | BEND4 | Tmem64 |
| MEGF11 | ORC5 | NGFR | Kcnk10 |
| POGLUT1 | BEND4 | SS18 | Pogz |
| BAZ2B | HOXA13 | POGZ | Rab11fip1 |
| TBC1D9B | CASC10 | SOCS5 | Cbx5 |
| KCNJ6 | H3F3B | ECE2 | Slc8a1 |
| TTC39A | ONECUT2 | CACNG2 | Dnajc13 |
| FAM184A | PHB | RGL2 | Pdia5 |
| YPEL3 | MMD | UBR5 | Bend4 |
| RND3 | CASC3 | PRKX | Slc35f3 |
| UBE2NL | SNX12 | MPP6 | Epb41l1 |
| C16orf52 | NXT2 | FAM184A | Dtx4 |
| ZBTB37 | SORL1 | PCNX1 | Srek1 |
| WEE1 | PPIF | EEF1AKMT4-ECE2 | Rcor3 |
| ONECUT2 | MOSPD3 | SLC22A23 | Rnf144a |
| TCEANC2 | AGFG1 | NRK | Unc13c |
| CDIP1 | MSANTD3-TMEFF1 | GAB1 | Neo1 |
| SPG21 | UBE2N | TUB | Sec24a |
| SH3BGRL2 | HLX | TEAD1 | Ptpn9 |
| H3F3B | TMEFF1 | DOT1L | Nemp1 |
| GRIN2D | GRIN2D | NFIL3 | Kdm7a |
| PHB | KCNJ6 | PDIA5 | Fam184a |
| GMPS | PPP4C | SLC6A1 | Mief1 |
| RAB20 | ADORA2B | GCC1 | Nrk |
| ZNF385D | SEC61A1 | AKIRIN1 | Rgl2 |
| LAPTM4B | CCDC71 | UNC13C | Unc80 |
| MOB1B | SP1 | CCDC88A | Tgfbr1 |
| BEND4 | SLC6A1 | RO60 | Sdha |
| ORC5 | GTF2A2 | MED13L | Msi2 |
| CA10 | TBC1D9B | RETREG3 | Ttc39a |
| SEC61A1 | AK2 | SOS1 | Neurod6 |
| NEK2 | ZNF462 | FAM126A | Frmpd3 |

| | | | |
|---|---|---|---|
| ECE1 | RNF38 | IRS1 | Ak2 |
| SNAP25 | RYBP | TTC39A | Abl2 |
| VANGL1 | UNC13C | CEP76 | Iglon5 |
| GALNT7 | SAMD10 | PHB | Ccdc88a |
| MIER2 | C16orf52 | RNF182 | Fam126b |
| GTF2A2 | ACVR1C | GATA6 | Tril |
| MOSPD3 | RAD51L3-RFFL | AK2 | Stk32a |
| MINPP1 | FBXO33 | PTPRB | Slc22a23 |
| CASP8 | SGPP1 | AK4 | Sgcb |
| PHGDH | LIN28A | GXYLT1 | Pdgfra |
| EML1 | CDIP1 | UGT8 | Ptprt |
| NXT2 | CYP39A1 | NEO1 | Pde3a |
| GLTP | CCDC92 | PDE3A | Gabra6 |
| UBE2N | NREP | GRIN2D | Ywhab |
| NHS | CA10 | ADCY2 | Gria3 |
| CABLES2 | NGFR | H3F3B | Nf1 |
| RNF38 | ARRDC4 | ZNF704 | Ak4 |
| AKIRIN1 | ST6GALNAC3 | TGFBR1 | Itsn2 |
| CCDC71 | NEK2 | XPR1 | Luc7l |
| PHF6 | TTC39A | CNOT6 | Kcnk2 |
| IGLON5 | DIRAS2 | NF1 | Eif2s2 |
| EFR3A | NRBF2 | VEGFC | Plagl2 |
| G6PC3 | VANGL1 | CXADR | Grik3 |
| BLOC1S5 | RAP1B | TRIL | Ube2n |
| PPP1CC | CNR1 | ARMC8 | Fbxw7 |
| CXADR | UBE2E2 | CABLES2 | Dpys |
| PPP4C | RFFL | SETD7 | Vangl2 |
| NREP | EML1 | SPTY2D1 | Ppm1e |
| SH2D3C | CABP1 | TTC9 | Acvr1c |
| VANGL2 | MIEF1 | RCOR3 | Setd7 |
| POGZ | PHF6 | PTPN9 | Rfxap |
| TMX1 | FAM134C | USP42 | Slc39a12 |
| RNF182 | SEC24A | EIF2S2 | Phb |
| PLAGL1 | TCEANC2 | ABL2 | Cts8 |
| RYBP | IGLON5 | GALNT7 | Atp8a1 |
| MDN1 | JAG1 | ROR1 | Retreg3 |
| ISL1 | KCNK2 | MIEF1 | Cdip1 |
| FNDC4 | ISL1 | USH2A | Ice1 |
| CCDC92 | NEUROD6 | YWHAB | Ephb2 |
| RGS17 | SLC35F1 | ERC2 | Dclre1c |
| GATA6 | PAIP2 | ARHGEF38 | Kmt2a |
| KCNK10 | CA7 | PDHX | Gigyf2 |
| DIRAS1 | FAM78A | NEK2 | Met |
| XCR1 | STK32B | ATP8A1 | Mapk8ip3 |

| | | | |
|---|---|---|---|
| CABP1 | BGN | MAPK14 | Car10 |
| THAP11 | BMI1 | NXT2 | Slc6a1 |
| RGL2 | AFF4 | NABP1 | Fut9 |
| ALDH4A1 | SSSCA1 | VANGL2 | Akirin1 |
| SEC24A | UBR5 | STOX2 | Tmem170b |
| TFEB | ZKSCAN2 | GRIK3 | Pgap1 |
| RAP1B | MOB1B | RNGTT | Nxt2 |
| F3 | RNF144A | NFX1 | Ing5 |
| SLC35F3 | EN1 | RYBP | Sim1 |
| ARRDC4 | VAV3 | COMMD3-BMI1 | Dcaf7 |
| FAM84B | MCF2L | FBLN5 | Cyp39a1 |
| CYP39A1 | SGMS1 | SLC39A7 | Strn4 |
| SP1 | RELN | FOXA3 | Adgrg6 |
| BMI1 | SMAD9 | BAG2 | Sema6d |
| PDE7B | PAX9 | FRYL | Stox2 |
| NEUROD6 | ABHD17B | STK32A | Smad9 |
| PNRC2 | SLC35F3 | PPM1E | Gltp |
| RCAN2 | ZFP36L1 | STRN4 | Kcnn3 |
| SLC6A1 | RP1-170O19.20 | PLCH1 | Rngtt |
| PDLIM2 | TMX1 | ARHGAP12 | Limk1 |
| RPS6KA5 | NHS | HIC1 | Fryl |
| SREK1 | KIAA1045 | SNAP25 | Mmd |
| WNK1 | WNK1 | CYP39A1 | Ticam1 |
| NGFR | ARHGAP12 | EPHB2 | Ppfia2 |
| BTG2 | GALNT3 | COL27A1 | Gcc1 |
| RP1-170O19.20 | COMMD3-BMI1 | KDM3A | Tmem25 |
| MSANTD3-TMEFF1 | MIER2 | AMER2 | Mdn1 |
| SRPK2 | KCND3 | PHF24 | Arhgef40 |
| NRBF2 | STK32A | FAM126B | Isl1 |
| ABHD12B | ARHGEF11 | MDN1 | Galnt3 |
| MVB12B | GPAM | RAB20 | Rsbn1l |
| FBXO33 | HIC1 | EML1 | Trove2 |
| TMEFF1 | NGLY1 | ISL1 | Kdm3a |
| KBTBD11 | GABRA6 | RNF38 | Snx12 |
| SOCS6 | UGT8 | USP49 | Armc8 |
| PKIA | DLGAP3 | TMEM64 | Mnt |
| ZFP36L1 | ELOVL6 | PPFIA2 | Erich5 |
| CASC10 | VANGL2 | SCAI | Ulk1 |
| SS18 | CHRM2 | SFXN2 | Vegfc |
| COMMD3-BMI1 | FAM222B | SORL1 | Pdhx |
| AFF4 | FBXW7 | TMEM167A | Gpr149 |
| OSBPL10 | RNF125 | ZNF652 | Trim12c |
| COL21A1 | UBE2W | UGCG | Sh3bgrl2 |
| FAM133B | FAM84B | MET | Zfp36l1 |

| | | | |
|---|---|---|---|
| SSH1 | PPAP2B | ARHGAP32 | Nemp2 |
| HOXA13 | CDH24 | RET | Krit1 |
| JAG1 | FAM155A | CASC3 | Spty2d1 |
| RELN | PDE1C | SLC7A11 | Cables2 |
| IGJ | SAMD12 | BAZ2B | Cox11 |
| PDHX | NAA15 | PLAGL2 | Samd10 |
| AK4 | TMEM25 | RECK | Rapgef2 |
| TRIM23 | CATSPERG | NAA50 | Colca2 |
| LIN7C | PPARG | MED12L | Nsd2 |
| UBE2E3 | ZBTB37 | SH2D3C | Ccdc92 |
| UBR5 | KIAA1199 | FAM155A | Mtss1l |
| RNF144A | KBTBD8 | GRIA4 | Casc3 |
| ABHD17B | GRIK3 | C20orf194 | Ppp1cc |
| SGMS1 | DLL4 | WNK1 | Csf1 |
| FBLN2 | TRABD | PLCL2 | Scai |
| HOXA5 | HOXA5 | MAPK8IP3 | 2310007B03Rik |
| HIC1 | SH2D3C | CECR2 | Tmtc2 |
| FAM155A | COL11A2 | PDPK1 | Pxmp2 |
| RASGEF1B | STIM2 | ZFP36L1 | Rps6ka5 |
| CA7 | YPEL2 | IRF4 | Kcne4 |
| ZNF488 | ALDH4A1 | LTBP1 | Cxadr |
| GREM1 | TMEM132E | CLPP | Nrep |
| TMTC2 | TRMT2A | ZFHX3 | Ubr1 |
| PAX9 | MTMR12 | VPS4B | Ikbip |
| UBE2W | ZNF385A | SNX12 | Syn3 |
| ATP8A1 | MTSS1L | GREM1 | Tmem240 |
| NGFRAP1 | TMEM64 | NRBF2 | Agfg1 |
| NRP2 | DCAF7 | NUS1 | Esr1 |
| SH3RF1 | NR2F6 | PDE10A | Wnk1 |
| HAND2 | DCX | RASGEF1B | Ltbp1 |
| GPAM | CTDSP2 | RHOT2 | Samd12 |
| GCNT2 | CRKL | CDIP1 | Pom121 |
| TNR | ARHGAP21 | SH3BGRL2 | Usp49 |
| FBXW7 | ZNF618 | FEM1B | Klhdc8a |
| CCDC50 | WEE1 | UNC80 | Ech1 |
| APLNR | CORO1C | RELN | Snap25 |
| MDM4 | SLC39A13 | ABCA12 | Pira1 |
| FAM134C | C10orf54 | IGSF3 | Sall4 |
| MSL1 | IGJ | DIRAS1 | Ncam1 |
| SCAMP3 | MYEF2 | CDH24 | Sirt1 |
| KCND3 | LETMD1 | ZNF800 | Colgalt2 |
| SOCS5 | GALNT7 | PDE7B | Arhgap21 |
| RNGTT | SOCS6 | PTPRT | Ttc30a1 |
| CTDSP2 | TLX1 | SP1 | Hoxa5 |

| | | | |
|---|---|---|---|
| ZNF385A | C19orf81 | RNF144A | Dcp2 |
| MAPK14 | HOXA9 | SAMD10 | Pira2 |
| UGT8 | TFEB | MAPKAPK3 | Nrbf2 |
| SMURF2 | FUBP3 | ARHGAP21 | Camta1 |
| SAMD10 | ABCB9 | SMARCA2 | Usp46 |
| GPR135 | BTG2 | FAM155B | Sp1 |
| CLCN6 | MDFI | SEC24A | Map2k7 |
| SLC39A11 | VEGFC | CREB1 | Ube2w |
| SP2 | SALL4 | NSD1 | Riok2 |
| CSRP2 | WNT3A | RSBN1L | Xirp2 |
| KITLG | XPR1 | CCNC | Slc10a7 |
| CLP1 | NIPAL4 | IL13RA1 | Itga5 |
| YPEL2 | NDST1 | ABCB9 | Lrguk |
| NR2F2 | PDHX | PGAP1 | Capza1 |
| SOX7 | HMGB3 | KBTBD11 | Mknk2 |
| LRAT | NGFRAP1 | UBR1 | Tmem167 |
| FAM222B | FOXQ1 | LITAF | Bcorl1 |
| SAYSD1 | CACNA2D3 | H3F3C | Tgds |
| CDS1 | PRICKLE1 | UBE2W | Osbpl2 |
| FAM193B | PDE8B | PIH1D3 | Crkl |
| HAO1 | FAM126B | COL21A1 | Phf20 |
| LDLR | HAO1 | MEGF11 | Eml1 |
| SSSCA1 | MATN3 | C20orf202 | Fn3krp |
| KPNA5 | KCNK10 | NREP | Plppr1 |
| HOXA9 | AK4 | MSL1 | Abcb9 |
| ZNF618 | MAP2K7 | CA10 | Nav2 |
| SYT1 | FAM155B | DCC | 1700025G04Rik |
| MATN3 | TMEM91 | PPP4C | Gys1 |
| IRS1 | PALM2 | MTMR4 | Sec61a1 |
| EPB41L4A | LRRC55 | UBE2N | Fam78a |
| STK24 | MEGF11 | GLTP | Car7 |
| PPARG | CSF1 | PPME1 | Med12l |
| FAM78A | EPB41L4A | SYT1 | Elmo1 |
| RAB33B | TTC9 | TMEM30A | Klrb1f |
| FBLN5 | MINPP1 | WDR7 | Tbc1d8 |
| UBE2D1 | CCDC28B | STK24 | Jag1 |
| SMAD9 | AKIRIN1 | ATXN10 | Chd2 |
| STIM2 | PKIA | GCC2 | Zfp664 |
| POU2F3 | RASGEF1B | FBXW7 | Ngfr |
| FAM172A | SP2 | CCNT2 | Ppig |
| CACNA2D3 | ZNF652 | DCP1A | Naa15 |
| PKIB | PELI2 | FAM177A1 | Ankrd40 |
| MARCH8 | PTPN5 | PNKD | Dcx |
| THOC1 | DVL2 | AMD1 | Zfp704 |

| | | | |
|---|---|---|---|
| GRIK3 | TRAPPC8 | CTDSP2 | Soga1 |
| ELMO1 | ARHGAP1 | MBTD1 | Peli2 |
| SLC26A11 | VWC2L | OSBPL10 | Dcc |
| XPR1 | GCC1 | BCORL1 | Fam120c |
| RAB24 | FAM102A | VANGL1 | Cecr2 |
| CBX5 | THRB | TXNIP | Pank1 |
| MMD | DOT1L | SREK1 | Slc7a14 |
| ELP6 | C7orf41 | FNDC4 | Stk40 |
| ADORA2B | SH3RF1 | SOX7 | Atxn10 |
| FOXQ1 | NR2F2 | C1QTNF7 | Zhx1 |
| DCAF7 | DCP2 | KCNN3 | Fam91a1 |
| PLEKHH2 | ECE1 | RPS6KB1 | Usp42 |
| NAA50 | FAM49B | TRIM23 | Hcn4 |
| ATP5G3 | NDUFS4 | FBLN2 | Nusap1 |
| GALNT3 | JHDM1D | DISC1 | Col27a1 |
| LIN28A | CITED2 | SEC61A1 | Tmem170 |
| NDST1 | FAM193B | SGMS1 | Rps6kb1 |
| CAPN7 | CD34 | MDH1B | Gab1 |
| FBXL20 | SAMD9L | SLC35F3 | Palld |
| MIEF1 | CABLES2 | ANKRD40 | Ankrd6 |
| DIRAS2 | ARPP21 | ABHD17C | Msl1 |
| NRIP2 | ZC3H12D | SIRT1 | Usf3 |
| AMD1 | SV2A | GSPT1 | 9130008F23Rik |
| CDH11 | TGFBR1 | NAV2 | Tmem178b |
| CORO1C | STK24 | NEMP2 | Acvr2a |
| SAMD12 | ULK1 | CKAP4 | Naa50 |
| ABCB9 | GRIA1 | ITGA5 | Mtdh |
| COX7B | SSH1 | AKR7A2 | Chtf8 |
| GSPT1 | STRN4 | ADAMTS5 | Car12 |
| CDH24 | TNPO1 | GALNT3 | Opn4 |
| EGR3 | NABP1 | PROSER2 | Ppp1r3d |
| ZNF462 | NFX1 | SP2 | Edn3 |
| MTSS1L | SYT1 | GRM5 | Bag2 |
| RAB27A | KIAA1033 | SPG21 | Kcnd3 |
| PDE3A | GREB1L | ZNF618 | Lbh |
| GPR61 | STK35 | HECTD1 | Glipr2 |
| ARPP21 | GPR135 | SSH1 | Plekhm3 |
| UNC13C | HGF | RAP1B | Csmd2 |
| CSDC2 | RPS6KA5 | SAMD12 | Dlk1 |
| ROR1 | KDM3A | UBA6 | Pds5b |
| SALL4 | GPR126 | HOXA5 | Kmt2c |
| OSER1 | MPPED2 | JAG1 | Nsd1 |
| TMEM25 | LRAT | HAPLN1 | Sertad2 |
| MTDH | C10orf137 | RND3 | Adamts18 |

| | | | |
|---|---|---|---|
| TNPO1 | MPP6 | EFR3A | Magi3 |
| SRSF9 | GRB2 | MATN3 | Vps4b |
| MDFI | ZNF24 | N4BP1 | 4930564D02Rik |
| KCNK2 | CACYBP | MLLT10 | Iffo2 |
| STAT1 | CDH11 | SH3RF1 | Gcc2 |
| SHISA6 | MTA3 | ZKSCAN2 | Hapln1 |
| C20orf194 | INSM1 | CEMIP | Melk |
| ZBTB34 | PDE7B | RERE | H3f3b |
| KCNA4 | ELMO1 | ERLEC1 | Gpd2 |
| RBPMS2 | MME | GABBR2 | Ncapg2 |
| HMGB3 | TPD52L2 | ZFP82 | Slc5a3 |
| TPD52L2 | E2F7 | AFF3 | Abhd17c |
| NABP1 | RAPGEF2 | GSK3B | Mdfi |
| ZNF652 | SNX18 | MYT1 | St6galnac3 |
| ZNF784 | AGFG2 | BMI1 | Pikfyve |
| CSF1 | KLF4 | LYPD3 | Vangl1 |
| NUS1 | PTPRB | TMC7 | Paip2 |
| TMEM229A | NUS1 | TMEM25 | Mmp1a |
| SMAP1 | FLRT3 | INO80D | Slc39a13 |
| TDP1 | NT5C1A | CACNA2D3 | Dpy19l3 |
| CACYBP | PAPPA | ITPKC | Tor1aip2 |
| ARHGAP12 | DCUN1D1 | MMD | Slu7 |
| GRIA1 | DROSHA | ST6GALNAC3 | Cep162 |
| FAM133A | PDS5B | MAP2K7 | Wbp1l |
| CBFB | MSL1 | SLC35F1 | Gnpnat1 |
| AGFG2 | ACTA2 | WEE1 | Kcnj3 |
| WDR7 | FBLN2 | LBH | Rgs8 |
| C19orf81 | EFR3A | PAX9 | Myt1 |
| DAZAP2 | NLN | FBXO30 | Irs1 |
| EN1 | OTX2 | PDS5B | Cpne8 |
| DDAH1 | ATP8A1 | NEUROD6 | Pparg |
| ACTA2 | PDE3A | APOLD1 | Pcdh20 |
| GALNT5 | IRS1 | EPB41L1 | Plekhh1 |
| DLGAP3 | MGAT1 | COPB2 | Rere |
| NDUFS4 | TMEM229A | STX7 | Wsb1 |
| UBR1 | SP4 | MINPP1 | Pde8b |
| FUBP3 | MAPK8IP3 | PLPPR1 | Hic1 |
| MAPK6 | AXIN1 | MNT | Aph1a |
| VPS4B | MDM4 | ALDH4A1 | Sgms1 |
| CITED2 | FAM104A | CA7 | Prr18 |
| PPME1 | CCNG1 | FAM78A | Adcy2 |
| LRRFIP1 | UGCG | HCN4 | Itpkc |
| KIAA1033 | CBX5 | DCX | Rbpms2 |
| EYA4 | HSPD1 | EYA4 | Nab1 |

| | | | |
|---|---|---|---|
| CRKL | HAPLN1 | GNS | Dnajc3 |
| PDE8B | ADAM10 | NPTXR | Zfhx3 |
| UNKL | LYPD3 | HMBOX1 | Bahd1 |
| JHDM1D | UPF1 | IFITM10 | Aff3 |
| TMEM132E | SLC39A11 | GPATCH2L | Syt4 |
| GPD2 | ARID1B | MOSPD3 | Cacna2d3 |
| LDLRAD3 | PCM1 | LHFPL3 | Ngdn |
| GOLM1 | MKNK2 | ING5 | Ypel2 |
| REPS1 | SPRY2 | RETREG1 | Selenon |
| C1orf52 | CDS1 | MTSS1L | Slc7a11 |
| GNS | TADA1 | C1orf21 | Tbc1d22b |
| ELOVL6 | PDE10A | ST14 | Rab36 |
| DCP1A | HBEGF | ARF3 | Tab3 |
| SLC39A13 | RLIM | SOCS6 | Tet1 |
| DCX | ZCCHC24 | OPA1 | Tub |
| WBP1L | MAFG | ZC3H12D | Clcn3 |
| UAP1 | PCP4L1 | DPY19L3 | Nipal4 |
| LIN28B | FRMPD3 | CHTF8 | Grm5 |
| FRMPD3 | HOXB8 | CCDC92 | Enah |
| MLLT10 | IFFO2 | MIPOL1 | Gng12 |
| STRN4 | DUSP5 | COLGALT2 | Slc35f1 |
| TMEM64 | TMCC1 | CRKL | Sh3rf1 |
| C8orf4 | ERMP1 | MED14 | Hlx |
| MME | RSBN1L | WBP1L | Cpeb3 |
| SNX18 | RPGRIP1L | GIGYF2 | Ugcg |
| PTPN5 | HSP90AA1 | SOGA1 | Swsap1 |
| KLF4 | NPEPPS | KIRREL1 | Plagl1 |
| ZEB1 | SET | KLHDC8A | Rnf139 |
| SRGAP2 | EFNA2 | KAT7 | Scaf11 |
| KLHDC8A | CDH5 | ARID1B | Nrp2 |
| UBE2F | DAZAP2 | CLDN18 | E2f7 |
| TRMT2A | FBRSL1 | TCEA1 | Mcmbp |
| UGCG | FAM222A | ZBTB20 | Btg2 |
| FBXO30 | SMAP1 | SLC39A11 | Ccdc71 |
| TAF4 | APLNR | STIM2 | Pik3r1 |
| GCC2 | BDP1 | SCAF11 | Xpr1 |
| DNAJC3 | CCDC88C | LPAR6 | Mybpc2 |
| HIPK2 | UNKL | SMAD9 | 1700066M21Rik |
| TRABD | MARCKS | NDST1 | Fbxo33 |
| BCORL1 | NCOA7 | DHTKD1 | Hip1 |
| FLRT3 | CSRP2 | B3GNT7 | Arrdc4 |
| ARHGEF11 | SMARCA2 | SLIT2 | Mtfr1 |
| FAM155B | CBFB | TMEM87A | Med13l |
| NLN | NFE2L2 | REPS1 | Rnf38 |

| | | | |
|---|---|---|---|
| CNR1 | STK39 | F3 | Tmem121b |
| RUNX1 | DDAH1 | EPB41L4A | Mier2 |
| OTX2 | VWC2 | HIP1 | Lrat |
| MPPED2 | GIMAP6 | USP46 | Rspo4 |
| TMCC1 | PIK3R1 | SHTN1 | Plxnc1 |
| KCNN3 | MYT1 | GTF2A2 | Cipc |
| E2F7 | ITPKC | ASPH | Steap3 |
| RRAS | PHLPP2 | GPD2 | Pard6b |
| TMEM91 | SUCO | MARK4 | Arhgap32 |
| PPFIA2 | NFIA | SASH1 | Med14 |
| ARL8B | ABL2 | SLC26A11 | Suz12 |
| STK35 | RP11-122A3.2 | ITCH | Ino80d |
| C2CD2 | GATA2 | KIAA1109 | Bub1b |
| TMEM151B | MLLT10 | SLC5A3 | Amer2 |
| AMMECR1L | MSN | NKX3-1 | Onecut2 |
| SV2A | CAMK1D | DPY19L4 | Sgpp1 |
| FAM102A | COL27A1 | SNX18 | Phactr4 |
| NAA15 | OAF | DPP10 | Stmn2 |
| CCDC149 | EDAR | RCAN2 | Syt1 |
| GOLGA6C | ITGA5 | G6PC3 | Adamts16 |
| ABL2 | SLC6A6 | TPPP | Nptx2 |
| RSBN1L | UBE2E3 | LIMK1 | Lpp |
| TEAD1 | ING5 | CBLB | Cnot6 |
| KIAA1045 | GNS | ENAH | Fbln2 |
| NGLY1 | GCC2 | KMT2A | Lonrf1 |
| POM121C | ADAMTS10 | SKA3 | Nabp1 |
| AGFG1 | SIRT1 | COL5A1 | Ildr2 |
| ING5 | SLCO3A1 | ONECUT2 | Ndufs4 |
| GNG12 | FAM133B | MME | Mcf2l |
| PPIF | BRSK1 | FXR2 | Fbrsl1 |
| ULK1 | ZHX1 | SLC24A4 | Ssbp2 |
| TSTD2 | NBEAL1 | SPATA17 | Itga4 |
| C17orf85 | SHOC2 | FSD2 | Tmem206 |
| SMPD3 | VPS4B | CSRP2 | Rybp |
| DUSP5 | TFAM | APBA2 | Plpp3 |
| TYW1 | KCNJ3 | CNTLN | Sfrp1 |
| ZKSCAN2 | AMMECR1L | ELMO1 | Opa1 |
| GAB1 | TMEM151B | NRXN1 | Trim23 |
| GOLGA6A | RND3 | MCF2L | Socs6 |
| MCF2L | GNG12 | RAB11FIP1 | Elfn2 |
| MPP6 | C8orf4 | UTP15 | Vip |
| RET | DYNLL2 | GAPT | Pitpnm2 |
| TGFBR1 | STOX2 | RAB39B | Rhbdf2 |
| WNK3 | CCT3 | STK35 | Mapkapk3 |

| | | | |
|---|---|---|---|
| PLXNC1 | RUNX1 | ECE1 | Nus1 |
| MAP2K7 | SNAP25 | MSTO1 | Snx18 |
| TMEM170A | WNK3 | TNFRSF10D | Foxa3 |
| SPRY2 | DTNA | BICC1 | Bmi1 |
| MYOCD | CLVS2 | NEURL4 | Rap1b |
| SP4 | SYT2 | RARA | Ifih1 |
| NPEPPS | SKOR1 | WNT3A | Abhd17b |
| ERMP1 | RRAS | MBOAT2 | Cdk18 |
| PPP4R2 | EYA4 | PPP1CC | Stk39 |
| RP11-122A3.2 | AMER2 | NRP2 | Zfp143 |
| GCC1 | FOXB1 | CA12 | Zfp385a |
| C7orf76 | KSR1 | TTC39B | Traf3 |
| GATAD2A | CD2AP | SERTAD2 | Bche |
| NAV3 | CAPZA1 | MIER2 | Sh2d3c |
| BMP2K | ITSN2 | SAMD9L | Ntrk3 |
| HBEGF | BCORL1 | CCNK | Cabp1 |
| KAT7 | EBF3 | E2F7 | Mageb4 |
| PCM1 | MSTN | MTDH | Mospd3 |
| C10orf137 | MNT | OPHN1 | Apaf1 |
| PXN | WBP1L | GLRA2 | Stag1 |
| UST | TAF4 | STAG1 | Stk35 |
| COLQ | PRR3 | ZDHHC17 | Insm1 |
| CCDC88C | ARHGEF37 | SLC39A13 | Fubp3 |
| DVL2 | NRK | UBN2 | Fam69a |
| DTX4 | PRDM12 | PITPNM2 | Rasgef1b |
| HOXB8 | MYOCD | GEM | Ammecr1l |
| ID3 | UBA2 | SMAD2 | Nr2f6 |
| NR2F6 | C7orf60 | TSR1 | Scml4 |
| ERC2 | CCDC50 | PANK1 | Ikzf1 |
| SLC6A6 | NPTX1 | ZNF705E | Myocd |
| CCNG1 | NETO1 | KCNA1 | Npbwr1 |
| MED14 | GSPT1 | TET1 | Lhfpl3 |
| TLK2 | STAG1 | UBE2V1 | Igsf6 |
| HGF | RP11-192H23.4 | YAF2 | Vav3 |
| THAP1 | MEPCE | DYNLL2 | Ikbke |
| SMARCA2 | MSI2 | PLD6 | Pde3b |
| IGSF3 | RNGTT | DBF4B | Shisa6 |
| CYB561D1 | MFSD2A | AARD | Adora2b |
| NEUROD4 | RBBP6 | NCOA7 | Kirrel |
| SAMD9L | RCAN2 | FAM102A | Ubn2 |
| ARF3 | PEAK1 | RIC3 | Ss18l1 |
| C15orf27 | MESDC1 | MTA3 | 2410004B18Rik |
| RPGRIP1L | ZBTB42 | PTPRQ | Ypel3 |
| H2AFY | AFF3 | PTAR1 | Fam102a |

| | | | |
|---|---|---|---|
| LASP1 | PELI3 | GCNT2 | Wnt3a |
| GPR125 | KLHL31 | DZIP1 | Hbegf |
| NIPAL4 | SOWAHA | ZNF84 | Dlgap3 |
| GRB2 | PLEKHM1 | DENND1B | Skap2 |
| GNB5 | CLCN6 | ATP6V1C1 | Ccnk |
| CCDC28B | ELL2 | KCTD4 | Pde10a |
| DDX6 | GFPT2 | CCN4 | Adamts20 |
| CCNT2 | LPPR1 | SBF2 | Zbtb39 |
| DDHD1 | PDPK1 | SETD5 | Lypd3 |
| FOXB1 | ZMAT3 | TMEM132E | Diras2 |
| LMNB1 | GPD2 | FBRSL1 | Eya1 |
| AMER2 | PSEN1 | CLCN3 | Ankrd10 |
| KSR1 | CNOT6 | SLITRK1 | Lpin1 |
| GOLGA8A | PDE3B | ZNF737 | Ppp4c |
| CNOT7 | TUB | CIPC | Znrf2 |
| WNT3A | CYB561D1 | SRP72 | Slco4a1 |
| C19orf40 | B3GNT7 | TBC1D22B | Greb1l |
| FAM126B | ST14 | YPEL3 | Gosr2 |
| RPN1 | TMEM170B | MED13 | Nr5a2 |
| SHOC2 | LCOR | C17orf102 | Elovl6 |
| RGPD6 | GLRA2 | IFFO2 | Tmem132e |
| TGOLN2 | POU2F3 | ADGRG6 | Creb1 |
| MSI2 | ERC2 | PITHD1 | Hoxa9 |
| NCS1 | DKK2 | SLC6A17 | Smap1 |
| DROSHA | NEURL4 | RGS6 | Clpp |
| STOX2 | C15orf27 | PCM1 | Sv2a |
| UBE2V1 | IPO7 | WNK3 | Zc3h12c |
| ENAH | FAM83F | PFKL | Ntrk2 |
| AGPAT3 | COLQ | MDFI | Ppme1 |
| HSPD1 | MPP2 | RBPMS2 | Necab1 |
| SASH1 | CSRNP1 | EBI3 | Prx |
| ADAMTS5 | INSR | NXF1 | Mtx3 |
| DCUN1D1 | LIMK1 | WASHC4 | Tmx1 |
| PPM1E | TMTC2 | SEMA6A | Grk6 |
| BRSK1 | SRL | PHF20 | Galnt7 |
| CNN3 | NEDD4 | ZHX1 | Cdh11 |
| RLIM | TNRC18 | PLXNC1 | Hectd1 |
| ITSN2 | MFHAS1 | ABHD17B | Clcn1 |
| NETO1 | LPPR5 | VAV3 | Tmem266 |
| BRWD3 | AGPAT3 | EDRF1 | Ctu1 |
| RSPO3 | NEURL1B | TCEANC2 | Stim2 |
| ZHX1 | ZNF800 | ZCCHC24 | Tnrc18 |
| MTA3 | CEP128 | STARD4 | Mphosph9 |
| PLCL2 | PPME1 | EYA1 | Sash1 |

| | | | |
|---|---|---|---|
| EIF5 | TLK2 | PDE12 | 1110004F10Rik |
| CNOT6 | NR5A2 | NR2F6 | Eml5 |
| ZNF608 | UBE2V1 | SEMA6D | Cited2 |
| KCNA1 | HOXC6 | ARHGAP28 | Neurl4 |
| SLC10A7 | TMEM110 | MPL | Ctdsp2 |
| BCL3 | CBFA2T3 | CDCP1 | Srgap2 |
| NFX1 | FAM65B | PIK3R1 | Csdc2 |
| PELI3 | RPS6KB1 | TMEM266 | Pdxk |
| PTGER3 | RORA | FOXO1 | Psmb1 |
| C11orf87 | DCP1A | LRAT | Zic4 |
| PTPRB | HECTD1 | OGFRL1 | Prkag1 |
| SYNDIG1 | ARX | LMTK2 | Ebf4 |
| INSM1 | BRPF3 | FAM172A | Cfap300 |
| NT5C1A | RAB11FIP1 | NCBP3 | Ppif |
| GLRA2 | FOXP2 | ANK1 | Msh4 |
| TUB | CCM2 | CPEB3 | Zfp827 |
| SYT2 | RCOR3 | TMBIM6 | Syndig1 |
| HECTD1 | DTX4 | RASL12 | Hoxa10 |
| EFNA2 | E2F3 | CYLD | Xrcc2 |
| RNPEPL1 | POM121C | MOB1B | Prom2 |
| LHX1 | KCNAB1 | SYNDIG1 | Stk24 |
| KDM3A | DDX6 | RGS8 | Traf6 |
| SLC16A10 | GMPS | AMMECR1L | Hdgfl3 |
| CHRM2 | CREB1 | ATL3 | Sep 02 |
| ADAM10 | ZBTB34 | CCR9 | Dcp1a |
| GXYLT1 | GLIPR2 | EHF | Ptprb |
| NPTXR | MED13 | LSM1 | 1600012H06Rik |
| NPAT | HOXA10 | NPTX1 | Plxnb1 |
| MBLAC2 | LRRC10B | YPEL2 | Rgs6 |
| PDK1 | NCAN | IBSP | Grm1 |
| SPTBN1 | LONRF1 | NTRK3 | Notch2 |
| BCL2L13 | ARHGAP19-SLIT1 | TNRC18 | Nrl |
| TLX1 | PPM1E | CAPZA1 | Cntln |
| HAPLN1 | STEAP3 | CBFB | Gfpt2 |
| SMIM14 | ARID2 | DLGAP3 | Psmd6 |
| FOXO4 | SHISA6 | AGO3 | Spindoc |
| DLL4 | BAHD1 | UBE2E3 | Agrn |
| CAPZA1 | SRP72 | PLEKHJ1 | Cbwd1 |
| GREB1L | FOXP4 | DUSP18 | B4galt1 |
| SET | TBC1D22B | CCT3 | Prex2 |
| MARCKS | CDK18 | PFKFB4 | Rnd3 |
| SMAD5 | MAP4K5 | SMURF2 | Mme |
| RAPGEF2 | HIP1 | DCAF7 | Nhsl2 |
| NCAN | DNAJC13 | SPTBN1 | Washc4 |

| | | | |
|---|---|---|---|
| GOLGA6D | LMBR1L | ACVR2A | Nin |
| ATXN10 | SREK1 | LIX1 | Zfp740 |
| CECR2 | APPBP2 | GNG12 | Itch |
| PANK1 | LHX1 | LY6G5B | Cdca7l |
| STAC | ACOT11 | IKZF2 | Rnft1 |
| EEPD1 | ELFN2 | SLU7 | Plcl2 |
| MET | SEMA6D | CNR1 | Utp15 |
| SERTAD2 | USP46 | CPD | Tia1 |
| NEURL1B | C17orf85 | PDGFRA | Gns |
| KBTBD8 | DPY19L3 | TAB3 | Slc26a2 |
| MIER3 | SYDE1 | TRAF3 | Stk32b |
| PALM2 | KIAA1109 | TFEB | Edrf1 |
| IFFO2 | DTX1 | HEG1 | Fam155a |
| VAV3 | RAI14 | ADCY3 | Sos1 |
| GABBR2 | ZADH2 | GATAD2A | Slc6a6 |
| USP46 | SFMBT2 | RBM4 | Dnajb1 |
| FGD6 | EIF5 | ELOVL6 | 2010111I01Rik |
| FAM105B | CPEB3 | FUBP3 | Mpped2 |
| KIRREL | HEG1 | MIER3 | Cemip |
| SHE | CTDSPL | PCTP | Gatad2a |
| LIMK1 | SRSF1 | MAGI3 | Tlk2 |
| LPPR1 | MAPKAPK3 | PTPN5 | Ttc28 |
| STAG1 | SIPA1L3 | GOT2 | Nxf1 |
| FOXN4 | SERTAD2 | HIPK2 | Pax9 |
| DEPDC1B | MED14 | UNC45B | Arid2 |
| MAPK8IP3 | PPFIA2 | TMEM91 | Cacng2 |
| NFE2L2 | CCAR2 | INSR | Ccdc28b |
| TRPV3 | PCDH9 | SFRP1 | Kpna3 |
| NEDD4 | CTDSPL2 | ZNF510 | Plekhg1 |
| PRMT8 | RNF141 | HOXA9 | Srp72 |
| AGO3 | TSTD2 | DCP2 | Prmt2 |
| EDEM3 | ELOVL4 | SCAMP3 | Lpar6 |
| PDPN | RASA2 | C11orf87 | Slk |
| CCM2 | CLSTN2 | PLPP3 | Nptx1 |
| ARID2 | FOXO4 | IPMK | Akap10 |
| METTL21A | SYNDIG1 | TM9SF2 | Ddx6 |
| SLC16A1 | GAD1 | LGALS3 | Aldh5a1 |
| ELL2 | CCNJ | BAHD1 | Ncoa7 |
| CKAP4 | TBC1D4 | RIPOR2 | Dcdc2a |
| EBF4 | TRPV3 | NPAS3 | Fbxw11 |
| GFPT2 | IRF4 | HOXA10 | Fam126a |
| ADCY2 | BCL3 | TCAIM | Fut2 |
| NCOA7 | ADCY2 | PTPRJ | Ncoa5 |
| TNRC18 | FOSB | BEX3 | Coq7 |

| | | | |
|---|---|---|---|
| SYDE1 | C11orf87 | KMT2C | Ccn4 |
| TGIF2 | IKZF2 | TLK2 | Chrnb3 |
| SUCO | NAV2 | RESF1 | Chst12 |
| PDPK1 | PLEKHH2 | SEMA7A | Sox7 |
| RNF141 | MBTD1 | H2AFY | Arhgef26 |
| AFAP1 | FGF14 | UNC5D | Cacnb2 |
| ADAMTS10 | EYA1 | ZBTB39 | Igsf3 |
| FGF14 | PLXNC1 | GRIP1 | Olfm3 |
| FOSB | NRP2 | ZNF696 | Lsm12 |
| VASH2 | FAM13A | NUDT6 | Acad12 |
| HOXA10 | TSC1 | GFPT2 | Slc39a11 |
| MED13 | MDGA1 | CCDC50 | Dnajc27 |
| TSC1 | ZNF608 | PPIL4 | Erc2 |
| PDGFRA | PTPRT | PTPN4 | Cep70 |
| ZBTB39 | ZC3H12B | COL3A1 | Fgf1 |
| ARX | GRM5 | DCK | Lasp1 |
| SETD7 | EDEM3 | DDX6 | Cmtm3 |
| LPCAT1 | LDLR | BMPR2 | Cfap97 |
| UPF1 | ERG | CCDC71 | Pgm2 |
| RERE | ADCY3 | UNKL | Arhgef11 |
| EHD3 | GDF11 | CASC10 | Abca1 |
| ZNF800 | PDLIM4 | FBXO33 | Iqsec1 |
| E2F6 | BRWD3 | PRKAG2 | Nfe2l2 |
| FOXP2 | PURA | COX6C | AI593442 |
| GAD1 | ADAM19 | ULK1 | Heg1 |
| SERBP1 | EPB41 | ABCB10 | Ptpn4 |
| PLEKHM1 | GABBR2 | TRIM32 | Cbfa2t3 |
| FAM222A | ADAMTSL1 | TPH2 | Mob3a |
| SKOR1 | HCN4 | MAGEA10 | Fads1 |
| CALN1 | TMEM189-UBE2V1 | USP15 | Oxgr1 |
| C6orf211 | TMEM189 | CDKN2A | L2hgdh |
| ELOVL4 | SOX11 | PTGER3 | Mcm6 |
| ITGA5 | RBM12B | MEGF6 | Lsm1 |
| TMEM189-UBE2V1 | STK38L | ADGRA3 | Sfmbt2 |
| TMEM189 | TET1 | ACTA2 | Traf1 |
| CCT3 | MAN2A1 | SLC25A25 | Trim66 |
| CAND1 | DAB2IP | ID2 | Kcna1 |
| TMEM123 | CLCN5 | DEF8 | Zfp677 |
| PPAP2B | KAT7 | FGD6 | Hsf2 |
| MESDC1 | SIK1 | LRRN4CL | Ythdf1 |
| RPS6KB1 | LSM12 | ACTR8 | Csrp2 |
| NEURL4 | PDGFRA | ARHGAP19 | Vsx1 |
| LONRF1 | TCF4 | RAB3B | Abl1 |

| | | | |
|---|---|---|---|
| PDE10A | SATB2 | NAA15 | Foxo1 |
| LMBR1L | ZEB1 | RAP2A | Brsk2 |
| PEAK1 | TIAL1 | STK40 | 4932438A13Rik |
| DYNLL2 | AFAP1 | TMCC1 | Zdhhc17 |
| PNISR | VGLL3 | RALGAPA2 | Clec14a |
| LCOR | KLHL18 | ST13 | Dkk2 |
| RREB1 | ZBTB39 | ZBTB34 | Dll4 |
| PDE3B | MAPK10 | NHS | Ehf |
| TRAPPC8 | USP42 | NETO2 | Arl4c |
| SPOPL | RAD54L2 | TRMT2A | Zfp800 |
| C17orf70 | STK40 | PELI2 | Baz2b |
| MAPKAPK3 | SS18L1 | UBE2K | Gpr156 |
| HOXC6 | FIGN | CDYL2 | Nyap2 |
| KCNAB1 | KCNN3 | VSIG10 | Gata2 |
| MAP4K5 | C1orf21 | FAM122B | Sfxn2 |
| CTDSPL2 | DFFA | PARK7 | Paqr9 |
| PARD6B | RUFY3 | KPNA3 | Dcun1d1 |
| APPBP2 | FXR2 | CPA4 | Dennd1b |
| ABCA1 | ABCA1 | SBNO1 | Adam22 |
| SORL1 | NXF1 | SDF2 | Fga |
| DMBX1 | MTMR4 | NOVA1 | Nrf1 |
| ZMYM4 | JMJD1C | NIPAL4 | Rxra |
| PAIP2B | SLC38A4 | CAB39 | Arhgef37 |
| TRIM67 | NPTX2 | CHKA | Twistnb |
| GDF6 | COLGALT2 | PLAGL1 | Fuca2 |
| PRPF19 | KPNA6 | PSPC1 | Smarca2 |
| TXNIP | FZD3 | PROZ | Ank1 |
| MAP3K4 | MAN1A1 | GOLGA6C | Zfp275 |
| ARHGAP21 | SH3PXD2A | RUNDC3A | Ngp |
| LANCL3 | NKX3-2 | MDM4 | Brpf3 |
| RUFY3 | AMD1 | NRARP | Appbp2 |
| PCDH9 | PTPN4 | ORMDL3 | Depdc1b |
| CSRNP1 | TBC1D25 | TM4SF20 | Kif21a |
| EYA1 | TGFBR3 | ANKS1A | Rara |
| GRAMD2 | NTN1 | TMX1 | Med13 |
| PIK3R1 | NRXN1 | TYW1 | Ccdc50 |
| CCNY | FURIN | USP25 | Sowaha |
| PAPSS2 | MYH15 | TMEM170A | Cd34 |
| TNIK | CCNY | IKZF1 | Ccdc141 |
| GSPT2 | ZC3H12C | DOCK11 | Kcnk1 |
| FBXO21 | GRIN2B | DNAJC13 | Pkia |
| CPEB3 | KIRREL | LPGAT1 | Lancl3 |
| SATB2 | UBE2F | ZEB1 | Ppil4 |
| TMEM110 | RERE | ZNF569 | Msrb3 |

| | | | |
|---|---|---|---|
| MGAT4B | NAV3 | NKX3-2 | Sep 12 |
| PRICKLE1 | CCDC85C | DVL2 | Hadha |
| WSB1 | MYBL1 | NDUFS4 | Aldh1l2 |
| LYSMD3 | ZZZ3 | KCNA4 | Mroh2a |
| SRL | ACER2 | CBFA2T3 | Litaf |
| CCNJ | SMG1 | NTNG1 | Hif3a |
| HLX | TRERF1 | TTC28 | Optn |
| SLC35F1 | MET | GATA2 | Rnf145 |
| ZNF24 | FGD6 | BRSK2 | Ston2 |
| CHST1 | WDTC1 | DMTF1 | Frem1 |
| MPP2 | NEO1 | FN1 | Epb41l4a |
| ARHGAP19-SLIT1 | SLC10A7 | CLP1 | Vcan |
| ELFN2 | NAIF1 | KMT5A | Taf4 |
| NF1 | SOS1 | EIF5 | Gm5615 |
| ARHGEF37 | MGAT4B | INPP5J | Cd8a |
| LPPR5 | SLK | PIAS2 | Scn3a |
| MNT | ARFGAP2 | INPP1 | Cptp |
| GPR75 | BBX | POU2F1 | Calm1 |
| PHF20 | EN2 | NEK6 | N4bp1 |
| PRELID2 | LMTK2 | TBC1D9B | Plscr1 |
| PURA | SCYL3 | JCHAIN | P2ry4 |
| SURF4 | EIF2S2 | ARFGEF1 | Mob1b |
| EPB41L2 | KIF5B | FAM102B | Orc5 |
| B3GNT7 | ADCY6 | CDH6 | Fam120b |
| NPTX1 | TMEM178B | SUZ12 | Pde7b |
| GHR | TOM1L2 | KCNJ3 | Thsd4 |
| TCF4 | TXNIP | SHISA6 | Pdpk1 |
| MGAT1 | WSB1 | ALG9 | Egln1 |
| SRSF1 | HIVEP3 | TENT4A | Mlycd |
| PLAG1 | INO80D | PRDM16 | Lynx1 |
| RALGAPA2 | CLCN3 | ETV3L | Itga9 |
| FBRSL1 | ZFP36L2 | HAND2 | Foxq1 |
| KIAA1109 | FRYL | LRRFIP1 | Crocc2 |
| MBTD1 | WIPF2 | UNC5C | Txnip |
| CHIC1 | SEMA6A | DMKN | Utp14b |
| REEP2 | NRARP | ADH7 | Fry |
| TAF5L | PGAP1 | SPATA2 | E330034G19Rik |
| IPO7 | ITCH | ATOH8 | Gxylt1 |
| CCNK | ELAVL2 | ARL8B | Zadh2 |
| PRR12 | DNAJB1 | SHE | Arnt |
| VAT1L | PHF20 | YTHDC1 | Atoh8 |
| TBC1D22B | RNF146 | OGA | Nup50 |
| MFHAS1 | MBOAT2 | ORC5 | Timm21 |
| NRK | CDYL2 | ELFN2 | Il17b |

| | | | |
|---|---|---|---|
| ITGA2 | USP49 | PKIA | Inka2 |
| NFIA | SLIT1 | LIN7C | Ano6 |
| HIVEP3 | MDN1 | KIAA1549L | E2f3 |
| RSBN1 | SLC5A3 | ILDR2 | Styk1 |
| NETO2 | QKI | SESN2 | Ccnb1 |
| KCNJ3 | ZFHX4 | TDRP | Fam84b |
| PDS5B | FAM129A | NECTIN3 | Zfp462 |
| PMF1-BGLAP | RREB1 | S100A7A | Zc3h12d |
| CDYL2 | ACVR2A | TCIM | Dnajb11 |
| DOCK11 | CREBRF | DCAF17 | Gtf2a2 |
| SIRT1 | UNC80 | UHRF2 | Dvl2 |
| CDK18 | UBR1 | FOXN3 | Focad |
| CLCN3 | SRGAP2 | TAF4 | Slc12a2 |
| CREBRF | BICC1 | SSX2B | Lhpp |
| LYPD3 | KMT2A | NEMP1 | Trio |
| FKBP14 | CEP135 | DDHD1 | Fbxo30 |
| EN2 | TNIK | PDE3B | Ptgfr |
| NPTX2 | ZNF516 | RMND5A | Homez |
| ZCCHC24 | NOL4 | CRB2 | 1700015F17Rik |
| BICC1 | DGKI | TMEM229A | Tspyl3 |
| ABCA12 | KCNA6 | TRIM6-TRIM34 | Gm14440 |
| SOX11 | TNR | PNISR | Unkl |
| SEMA6A | IKZF5 | MAST4 | Smim20 |
| ZZZ3 | GAB1 | TRIM34 | Tmcc1 |
| PPIG | FAM126A | SREBF2 | Ccnj |
| TOX4 | LOX | NLRP2B | Plcxd2 |
| GORASP1 | IKZF1 | RXRA | Inpp5j |
| ARID3A | LSAMP | LIN28B | Pdia6 |
| FRYL | SHANK3 | TPM1 | Zfp618 |
| PLEKHH1 | MARCH8 | ERG | Luc7l2 |
| NKX3-2 | ZNF827 | CPLX3 | Rab35 |
| CLCN5 | POU2F1 | DKK2 | Tmem91 |
| C6orf223 | SLC7A11 | SSX2 | Bcas1 |
| MEPCE | KMT2C | FAM206A | Rffl |
| DNAJC13 | GREM1 | ZNF792 | Eepd1 |
| GPR133 | NF1 | PLAG1 | Kif5b |
| TFDP2 | ALDH1L2 | C1orf52 | Unc5d |
| FIGN | MSI1 | TBC1D1 | Orc3 |
| PELI2 | DOCK11 | KIAA1211L | Atg2a |
| E2F3 | C11orf84 | ADAMTS10 | Smg1 |
| OAF | ZNF638 | TMUB1 | Pde1c |
| SGPP1 | IGSF3 | ARHGEF26 | Zeb1 |
| CREB1 | ARL8B | HBEGF | Myef2 |
| RAD54L2 | LARP4 | RASAL2 | Jmjd1c |

| | | | |
|---|---|---|---|
| EPB41 | ATL3 | IL12RB2 | Tsc1 |
| SNAI1 | COL3A1 | CRTC1 | Ripor2 |
| DTNA | ATP2B1 | RFX3 | Tbc1d9b |
| PEG10 | APBA2 | ELAVL2 | Cdc42 |
| C7orf60 | RSBN1 | AIF1L | Ptpre |
| MAPK1 | XIRP2 | BET1L | Arl8b |
| GNA13 | E2F6 | ALDH5A1 | Herc3 |
| STK32B | ITGA2 | WDFY1 | Lin7c |
| FOXP4 | EFNB2 | SAR1A | Dixdc1 |
| CPEB4 | LPCAT1 | TRIO | Dym |
| HNRNPF | SASH1 | TBX3 | Mybl1 |
| IKZF2 | BMP2K | WIPF2 | E130308A19Rik |
| MAN2A2 | EHF | MS4A2 | Cxcl13 |
| RAB39B | CCDC120 | CCDC85C | 6430571L13Rik |
| BHLHE41 | NFASC | RBM33 | Larp4 |
| LMTK2 | CRTC1 | MTMR10 | Mbtd1 |
| CCDC120 | ENAH | POM121 | Wdr60 |
| NPAS3 | TSPAN9 | PDK1 | Letmd1 |
| RASD2 | ZNF275 | GALNT13 | Cyp2b23 |
| FXR2 | ZNF664 | ZFP36L2 | Pigk |
| IKZF5 | CNNM2 | KIAA1210 | Rfpl4 |
| TRAF3 | MANEAL | RREB1 | Rad51c |
| PGM2L1 | PLAG1 | ELL2 | Mal2 |
| GRM5 | UBXN7 | TMCC3 | Atad3a |
| MYT1 | NFIB | ZNF385A | Ccnc |
| LZTS3 | NPAS3 | YY1AP1 | Actl6b |
| ANK2 | RIMS3 | KCNAB2 | Nyx |
| RAB11FIP1 | RORB | FAM13B | Arfgef1 |
| FEM1B | ST8SIA4 | GNG2 | Ints6 |
| INSR | CTH | PBXIP1 | Cab39 |
| DTX1 | JOSD1 | SPOUT1 | Zbtb33 |
| ADD2 | LZTS3 | NR5A2 | Furin |
| MAPK10 | SETD7 | TBC1D8B | Fen1 |
| MTMR4 | PGM2L1 | PLXND1 | Synpr |
| FUT9 | PDE1B | RAB3IP | Timm29 |
| BAK1 | MED1 | PPIF | Reps1 |
| CD2AP | NPR1 | CITED2 | Aff1 |
| PLA2G15 | KIF5C | JMJD1C | Dnase2b |
| LSAMP | MIER3 | E2F6 | Tmprss11f |
| FAM65B | N4BP1 | PDE8B | Fhod3 |
| SOWAHA | FRMD4A | FRS3 | Zfp516 |
| TBC1D4 | NCOA1 | GRK6 | Gm14295 |
| MYBL1 | MEIS2 | COL11A2 | Ell2 |
| CEP135 | TANC2 | CLCN5 | Asap1 |

| | | | |
|---|---|---|---|
| PAPPA | GUCY1A2 | TMED5 | Mier3 |
| TIAL1 | BRSK2 | MYBL1 | Tmem9b |
| DAB2IP | RPL36A-HNRNPH2 | KPNB1 | Serpinb6b |
| KIAA1551 | PTPN9 | FANCA | Ncbp3 |
| ATL3 | BMP3 | LRRC57 | Fam19a3 |
| DCP2 | NR1D2 | DPH6 | Nipsnap3b |
| HCN4 | PAK2 | USP51 | Rreb1 |
| RAB15 | ZFHX3 | LDLRAD3 | Coro1c |
| BMPR2 | KCNK12 | NOD1 | Ppp2r2d |
| CLSTN2 | KCNA1 | CRB1 | Tial1 |
| PPP4R1L | DST | SS18L1 | Mrpl35 |
| ZBTB42 | NEK6 | GOLGA8A | Lifr |
| DGKE | ASAP1 | FURIN | Megf11 |
| SYNRG | SPTY2D1 | ZNF140 | Map3k20 |
| CBFA2T3 | NETO2 | ZNF329 | Slitrk1 |
| MKNK2 | RARA | STK39 | Naa11 |
| WIPF2 | DLG2 | NOL4 | Usp25 |
| LSM12 | ANO6 | ZNF577 | C2cd2 |
| GJA3 | NCAM1 | ZNF426 | Ttc39b |
| CTDSPL | ARSG | POM121C | Rundc3a |
| TBC1D25 | FAM177A1 | TMEM87B | Nek4 |
| CDR2L | RXRA | STAB2 | Gsk3b |
| AGRN | ATP9A | MCTS1 | Man2a1 |
| RNF146 | KIAA1549 | KITLG | Mpzl2 |
| SLC29A4 | OPA1 | EBF4 | Pnisr |
| DPY19L3 | PRDM16 | MVB12B | Sh3d19 |
| EBF3 | LIFR | DLL4 | Tm9sf3 |
| ERG | EEPD1 | SLC5A9 | Ndst3 |
| ABHD5 | CCDC88A | EPB41 | Dsc2 |
| WDTC1 | RAB14 | FAM222B | Jchain |
| TAL1 | EPHB2 | ADCY6 | Stx16 |
| NR5A2 | KPNA3 | USF3 | Mrps36 |
| MUC19 | PPP1R9A | CDK18 | Crebrf |
| BRPF3 | NFAT5 | CREBRF | Flrt2 |
| KIAA1147 | HIPK3 | BLOC1S5 | Golt1a |
| GPR126 | ADCYAP1R1 | TIAL1 | AB124611 |
| RFX3 | MAPK1 | FOXO4 | Neto2 |
| RORB | TPPP | IGF2BP3 | Sp2 |
| AFF3 | PLAGL2 | PRKCB | Cyp2j5 |
| SLC7A11 | PTBP2 | PGM2L1 | Efnb3 |
| GSK3B | MED12L | TSC1 | Nap1l2 |
| CCDC85C | LIN7A | ZNF286A | Rhoj |
| PTPRT | C16orf70 | ITSN2 | Thrb |
| LMX1B | PITPNM2 | FOXP4 | Mtmr10 |

| | | | |
|---|---|---|---|
| OTUD7A | NFATC1 | PSMA1 | 4930519G04Rik |
| FAM129A | SUZ12 | GOLGA6A | Fbxo32 |
| KLHL31 | KLF3 | CADM1 | Lair1 |
| PIK3C2A | PCNX | COX18 | Pnkd |
| HNF4G | SLC9A7 | ZNF500 | Rsad2 |
| FAM13A | OTUD7A | COX8A | Rgs1 |
| CRTC1 | CXADR | GOLGA6B | Fam124a |
| TTC9 | BEND3 | ZNF860 | Lmtk2 |
| NXF1 | ZNF704 | GOLGA6D | Elavl2 |
| LEP | UBN2 | BAX | Sec22a |
| TAOK1 | PLEKHH1 | NUP210 | Col18a1 |
| HIP1 | RBP2 | PROCR | Siglech |
| NOL4 | PCDH1 | CISD2 | Efemp1 |
| ATP10B | PIK3C2A | NAB1 | Lmln |
| AXIN1 | FOXN3 | ANKDD1A | Kcna6 |
| QKI | SCAI | LSM12 | Rsl24d1 |
| SRP72 | TTC28 | GPATCH11 | Wnt1 |
| PCNX | SIX1 | ADGRF2 | Efr3a |
| KIAA1199 | CDH6 | GRAMD1B | 1700071K01Rik |
| DKK2 | BAZ2B | INSM1 | Tfeb |
| ITPKC | EPB41L1 | SGPP1 | Trappc8 |
| EDAR | C1orf52 | GOLM1 | Krtap26-1 |
| KMT2C | SLC8A1 | SHANK3 | Xrn1 |
| SLC38A4 | PPP2R1B | ZBED6CL | Syt3 |
| GATA2 | LGI2 | NCOA1 | Trim29 |
| FAM126A | TXLNG | ARMC3 | B3gntl1 |
| NEO1 | SFPQ | IGFBPL1 | Alg11 |
| ATP2B1 | NAA50 | SATB2 | Adamts19 |
| SIK1 | TMEM248 | PLEKHM1 | Otx2 |
| KPNB1 | AGO3 | E2F3 | Wnk2 |
| FZD3 | NSD1 | RAB3C | Myl12b |
| SLC25A25 | CHKA | CABP1 | Golm1 |
| ALDH1L2 | GFPT1 | HYOU1 | Dicer1 |
| MBOAT2 | SLC25A29 | BMP3 | Upk1b |
| HEG1 | AGO2 | FUT9 | Tmem229a |
| NRARP | MED13L | FAM120C | Mthfd1l |
| SLC39A10 | SFRP1 | RUVBL2 | Ipo11 |
| EIF2S2 | BAG2 | EN1 | Gtf2a1 |
| SCN3B | SEMA7A | DNAJC3 | Zfr |
| MICU3 | ATXN10 | PLPPR5 | Ipo5 |
| GRIN2B | FAM105B | SLC49A4 | Cntrl |
| EFNB2 | ILDR2 | ZNF37A | Pcm1 |
| NFIB | MAPK6 | OSER1 | Stxbp3 |
| COLGALT2 | EHD3 | FBLN1 | Wnk3 |

| | | | |
|---|---|---|---|
| JMJD1C | HNRNPF | MAP3K19 | Zfp606 |
| GGA2 | IL6ST | RNF139 | Zbtb42 |
| CAMK1D | SLITRK3 | IQSEC1 | Arpp21 |
| SAR1A | KIAA1549L | MYOCD | Rmnd5a |
| ELAVL2 | GSK3B | DAP3 | Lin28a |
| PAPD7 | BMPR2 | PPP1R3B | Hook3 |
| ZC3H12B | ARID4B | BEAN1 | Rpgr |
| ZNF280C | LMX1B | PDXK | Gria4 |
| GDF11 | DRP2 | ZNF34 | Slc1a2 |
| KIAA0232 | SPRY3 | ALDH1L2 | Ndst1 |
| ZNF704 | SMCR8 | LPCAT1 | Tor1b |
| KLF3 | C17orf70 | KIAA1191 | Mta3 |
| USP42 | PAPD7 | NKAIN1 | Mpp2 |
| PCGF3 | ARFGEF1 | SYT4 | Erv3 |
| COL11A2 | FUBP1 | TTC3 | Eif1ad |
| RORA | TMED5 | MICAL3 | Ccl11 |
| C1orf21 | SLC22A23 | C14orf93 | Gm13889 |
| SIPA1L3 | VCAN | GNA13 | Pabpc1l |
| GPRIN2 | COL26A1 | C1orf229 | Klhl4 |
| LARP4 | RAD51D | OTX2 | Clec2h |
| ZC3H12D | PLXND1 | SMAD5 | Agtrap |
| NTN1 | SPATA2 | LAPTM4B | Tmem159 |
| DPYSL5 | SYT7 | GPATCH2 | Mylk4 |
| RAB4A | MTDH | G6PC2 | Cep72 |
| ZFP36L2 | PNISR | FAM84B | Nova1 |
| IKZF1 | SS18 | G0S2 | Ncoa4 |
| TET1 | GAREM | CCNJ | Adam19 |
| SCN1A | REPS2 | JADE2 | Rora |
| CDH5 | CA12 | NOL9 | Chst11 |
| MSI1 | NEBL | ZIC5 | Appl2 |
| VCL | RGL2 | GATC | Gspt1 |
| MYH10 | WDFY2 | NEURL1B | Dglucy |
| SS18L1 | PTCHD1 | TMEM9B | Tgm3 |
| STK39 | KIAA0232 | CREB3L2 | Ctnnd2 |
| BEND3 | PLEKHA3 | HMGB3 | Hs1bp3 |
| ITGA8 | TMBIM6 | LONRF1 | Tox4 |
| NFASC | POU3F3 | GPR161 | Clock |
| PVRL4 | PPARGC1A | BTBD3 | Map4k5 |
| PLAA | SMAD2 | RELT | Cul4b |
| THRB | ZNF236 | SNIP1 | Camk1d |
| ACVR2A | HSD17B8 | NMB | Mrps12 |
| FURIN | TACC1 | ASAH1 | Syne3 |
| ZADH2 | MBD5 | PRICKLE2 | Hspd1 |
| CABLES1 | CNOT1 | FAM133B | Inpp5e |

| | | | |
|---|---|---|---|
| KMT2A | SOX6 | APPBP2 | Rbm33 |
| SLIT2 | ALG9 | ZNF470 | Wdtc1 |
| MAN2A1 | IRS4 | TM2D3 | Zfp764 |
| ADAM19 | MXI1 | UBE2F | Rad54l2 |
| GOLGA6L10 | PIK3CA | SLFN13 | Igsf9b |
| RP11-192H23.4 | CPD | RGPD6 | Jcad |
| PTPN4 | DLK1 | RNASEH2C | Pde1a |
| FAM53C | PRR12 | GOLGA6L4 | Tmcc3 |
| COL3A1 | FADS1 | ARID2 | Tbck |
| PTGFRN | KCNMB4 | FADD | Rbm43 |
| STK40 | MAPRE2 | GOLGA6L10 | Zfp24 |
| DGKI | CKAP4 | SUSD1 | Xrcc6 |
| VGLL3 | ERP44 | TRAPPC8 | Oprl1 |
| UBN2 | URGCP | FOXQ1 | Adamts5 |
| ADCY6 | RPN1 | ZKSCAN4 | Ostf1 |
| ZMAT3 | NIPBL | RGPD5 | Cmtm6 |
| PDP2 | DGCR14 | LDLRAP1 | Rab27a |
| ZC3H12C | CDON | MAP4K5 | Slc34a1 |
| TMEM170B | CCNK | ELOVL4 | Bloc1s3 |
| TMEM194B | TSPAN14 | SLC7A1 | Nepro |
| INO80D | PPIG | FAM13A | Zfp52 |
| SCYL3 | FCHSD1 | RCOR1 | Zfp324 |
| SLC7A14 | CADM1 | CDR2L | Deptor |
| NRXN1 | SLC24A4 | FAAP100 | Mtmr12 |
| DENND5B | ZBTB20 | PPP2R2D | Trp53bp1 |
| TBC1D13 | ATXN3 | NME4 | Ralbp1 |
| SLCO3A1 | ROBO1 | SPOPL | Chst7 |
| HUNK | KCMF1 | CAPN7 | Vsig10 |
| DIDO1 | CPEB4 | ID4 | Lrrc75b |
| KPNA6 | MAP3K2 | LRP2BP | Ptpn14 |
| USP49 | CSE1L | SLC10A7 | Klhl12 |
| SMG1 | CACNG2 | CTPS2 | Vezt |
| PDE1B | CLPP | SLC6A6 | Krtap4-7 |
| STEAP3 | WISP1 | IKZF5 | Tmeff1 |
| MANEAL | PFKM | TMOD2 | Chd6 |
| RARA | CDC6 | CASP1 | Slco5a1 |
| ACVR1C | DDI2 | NEBL | Slc36a4 |
| CNNM2 | PAX6 | INAVA | Chek1 |
| DCBLD2 | CCDC6 | EFR3B | Dynlt1a |
| SOS1 | GPR174 | VIP | D430041D05Rik |
| PSEN1 | DCC | GYS1 | Atl3 |
| BBX | KLHDC8A | PPP2R3A | Fam222a |
| KCNA6 | CHST1 | SLC9A4 | Msn |
| RIMS3 | CACNA1A | TRABD2B | Npas3 |

| | | | |
|---|---|---|---|
| RASA2 | TRIP4 | CTU1 | Ehd3 |
| RP13-996F3.5 | SOCS5 | SMPD4 | Ckap4 |
| KPNA3 | LRRFIP1 | ZNF783 | Fbxl18 |
| GAREM | CNOT7 | SNRPE | Pnrc1 |
| NBEAL1 | | FCHSD2 | Tmem268 |
| ZFHX4 | | KCNMB4 | Gldc |
| SPTY2D1 | | OR11A1 | Jakmip3 |
| SFMBT2 | | CFC1 | Azi2 |
| MED1 | | APOF | Arhgap19 |
| BRSK2 | | AGAP1 | Vmn2r89 |
| VWDE | | ARL15 | Oaf |
| DNAJB1 | | MBNL2 | Fbxl14 |
| DOT1L | | FAM71F2 | Pfkfb2 |
| FAM177A1 | | BTG2 | Tceanc2 |
| ZNF638 | | KCNK6 | Ulbp1 |
| PGAP1 | | EDNRA | Ifng |
| KIAA1549 | | CCNG1 | Glra2 |
| POU2F1 | | TWISTNB | Slc26a1 |
| MN1 | | SIX1 | Ptpn5 |
| N4BP1 | | LINGO4 | Zfp36l2 |
| PDK3 | | CCDC28B | Lox |
| MICALCL | | FAM222A | Ss18 |
| NAIF1 | | BRAP | Fxr2 |
| NMRK1 | | DTNA | Rlim |
| SLK | | SLC39A10 | Impg1 |
| SCAI | | HOXA13 | Sptbn4 |
| APBA2 | | SH3GL3 | Itsn1 |
| SLC5A3 | | ZBTB14 | Cnr1 |
| TMEM178B | | SLC9A7 | Tcim |
| MAFG | | MECP2 | Kif5c |
| BMP3 | | DUSP5 | Agap1 |
| NACC2 | | CREG2 | Prickle1 |
| SLC25A16 | | CSDC2 | Tgfbr3 |
| MEIS2 | | NTRK2 | Wee1 |
| C7orf41 | | TRAF1 | Rnasel |
| TACC1 | | PRLR | Amot |
| CSE1L | | RIMS3 | Pfkfb4 |
| PTBP2 | | SDK2 | Txnl4a |
| NSD1 | | ACOT13 | Mcph1 |
| KIF3A | | WTAP | Rab34 |
| SRCIN1 | | WDTC1 | Ero1lb |
| PHC2 | | APCDD1L | Epha5 |
| SCD | | BHLHB9 | En1 |
| SEMA6D | | CDH11 | Peak1 |

| | | |
|---|---|---|
| EPHB2 | C17orf51 | Gtf2ird2 |
| PHLPP2 | PWWP3A | Zcchc24 |
| ADCY3 | CCM2 | Sema6a |
| MED13L | DHCR24 | Cyp2d22 |
| PRDM16 | PIGZ | R3hcc1l |
| TNRC6B | MBD3 | Nadk2 |
| SLIT1 | PALM2 | Mtmr4 |
| ATXN3 | PLEKHH1 | Sox11 |
| THAP2 | MGAT1 | Mrgprb2 |
| C16orf70 | ADRA1A | Slc12a7 |
| ARFGEF1 | MR1 | Slc35e1 |
| NFAT5 | TRAPPC4 | Efna4 |
| HIPK3 | NECAB1 | Nek6 |
| CCDC88A | IBA57 | Hsp90aa1 |
| ZNF395 | XIRP1 | Fndc4 |
| DLG2 | STOX1 | Mbnl3 |
| PER2 | TMEM121B | Aak1 |
| LOX | KLHL7 | Nr1d2 |
| NEK6 | TSEN34 | Zfp280b |
| ARMC8 | ZNF546 | Rdx |
| BAHD1 | C2CD2 | Fgf12 |
| PRCP | TACR1 | Isg20l2 |
| MARCH5 | HTT | Trappc10 |
| MED12L | SLX4IP | Lpar2 |
| RPL36A-HNRNPH2 | ZNF627 | Gpr17 |
| PHF15 | EGFR | Gmps |
| NCOA1 | HERC3 | Zdbf2 |
| SPTLC2 | PON2 | Ces2e |
| DRP2 | THAP11 | Reps2 |
| FRMD4A | DAD1 | Zfp488 |
| CTH | ATG2A | Crxos |
| OPA1 | AGFG1 | Tmem140 |
| NAV2 | AEN | Ercc4 |
| KIAA1549L | PNRC2 | Kbtbd8 |
| PTPN9 | POU2F3 | Fam169a |
| URGCP | PHLDA2 | Sema7a |
| FAM73B | KSR2 | Ahi1 |
| C11orf84 | PEAK1 | Actbl2 |
| TEX2 | SEMA4D | Hyou1 |
| GPRIN3 | CHST11 | Ctc1 |
| SUZ12 | RGPD4 | Steap2 |
| CRP | ARID5B | Tubgcp3 |
| SIX1 | NAP1L2 | Gm5592 |
| TANC2 | PPARG | Cpne3 |

| | | |
|---|---|---|
| BACE1 | PAPPA | Ppara |
| COL27A1 | PIK3CA | Hnrnpr |
| POTEG | TMEM170B | Cd2ap |
| RAI14 | BCL3 | Mllt10 |
| ZBTB14 | OIT3 | Kndc1 |
| MXI1 | ERO1B | Fem1a |
| PDLIM4 | ABCB5 | Pou2f1 |
| NR1D2 | MFSD6 | Stk4 |
| UNC80 | CAMTA1 | Shc4 |
| POTEM | TYRP1 | Frs3 |
| STK38L | EARS2 | Map3k14 |
| PPP1R9A | TTC17 | Bhlhe22 |
| LRP6 | FCRL2 | Spry2 |
| ZFHX3 | IL17RA | Map2k4 |
| SLC8A1 | NR2F2 | Efhd2 |
| PITPNM2 | FOSB | Rabggta |
| SFRP1 | ZNF20 | Gabrg2 |
| SEMA7A | PTCH1 | Nrarp |
| SFPQ | PRMT8 | Arxes1 |
| GOLGA6L4 | COG7 | Adcyap1r1 |
| NUP210 | MKNK2 | Man1a |
| KCNK12 | EDEM3 | Zfp442 |
| ST8SIA4 | LDLR | Il1r1 |
| ZNF827 | NRIP2 | Chrdl1 |
| PLCE1 | MAVS | Vps13a |
| ACOT11 | GJD3 | En2 |
| DST | WDR36 | Med28 |
| GFPT1 | TMEM237 | Arhgef6 |
| PTCHD1 | SPRY2 | Foxred2 |
| KCTD16 | CD276 | Adam10 |
| FAM192A | KCTD8 | Mtf2 |
| MBD5 | LRRTM2 | Ptger4 |
| FAXC | CAMK1D | Gm5114 |
| SPATA2 | AGRN | Gja3 |
| ASAP1 | RHOT1 | Smad2 |
| NCAM1 | CAND1 | Fndc8 |
| TAS2R5 | SOWAHA | Nol4 |
| DDI2 | ENPP1 | Marveld3 |
| PLXND1 | ADAMTS16 | Lrrc18 |
| TERF2 | PSEN1 | Tmem45a |
| TMED5 | NPTX2 | Man2b1 |
| AGO2 | ERP27 | Mpv17l |
| TXLNG | FSTL3 | Casd1 |
| RCOR3 | CLOCK | Eya4 |

| | | |
|---|---|---|
| FUBP1 | ANHX | Nr2c2ap |
| ATP11B | MOCS3 | Map1s |
| CPD | E4F1 | Gal3st2 |
| TGFBR3 | ARHGAP23 | Ppip5k2 |
| TRERF1 | POLM | Tmem150b |
| UBXN7 | RAD54L2 | Zmym5 |
| MAGI3 | FKBP3 | Rpgrip1l |
| KLHL18 | CLDN22 | Adra1d |
| PLAGL2 | NR1D2 | Amd1 |
| EPB41L1 | PDE6A | Il2ra |
| WDFY2 | STAC | Mfsd9 |
| CDS2 | LMBR1L | Cib1 |
| PTCHD4 | ARID3B | Atp11c |
| SLC22A23 | ZC3H12C | Kcnj8 |
| SHH | EPHA10 | Gpr174 |
| LIFR | DDX3Y | Wipf1 |
| CNTN2 | MYH10 | Wdr92 |
| ARID4B | VASH2 | Mgat1 |
| SH3PXD2A | TGIF2 | Elovl4 |
| SPRY3 | CACNA1C | Cct3 |
| ONECUT3 | ANKRD24 | BC035044 |
| ZNF185 | ADH5 | Fnbp1 |
| DUSP13 | SLC36A1 | 0610040J01Rik |
| ATP9A | ABCA1 | Rassf5 |
| RXRA | HMOX1 | Rxfp3 |
| CDON | RAPGEF2 | Cdh5 |
| SLC9A7 | HOXC6 | Tmem178 |
| FOSL2 | PVR | Gucy2c |
| ZBTB3 | MLPH | Polr2l |
| FOXN3 | MAGEB16 | Ccng1 |
| TMEM248 | CHD2 | Klhl31 |
| SLITRK3 | SPTLC2 | Fem1b |
| ALG9 | C18orf65 | Nedd4l |
| PCDH1 | ARX | Dmac2 |
| P2RY2 | TSPAN13 | Igsf10 |
| CA12 | STYK1 | Eif4e1b |
| ZBTB20 | KCNA6 | Cd244a |
| ILDR2 | RSPO3 | Mfsd4b2 |
| PGP | KDM4C | Aqp11 |
| TMBIM6 | WSB1 | Adcy6 |
| NAPEPLD | SPX | Arhgap1 |
| BZW1 | RGS1 | Srsf12 |
| NEBL | CBX5 | Swt1 |
| IGF1 | MAN2A1 | Pappa |

| | | |
|---|---|---|
| SMAD2 | RARB | Zkscan8 |
| RBP2 | TSTD2 | Trabd2b |
| HTT | HSPD1 | Gk5 |
| RAD51D | TNRC6B | Tbp |
| AP5M1 | ZNF124 | Cspp1 |
| KAT6A | DEPDC4 | Rab11b |
| PPARGC1A | SCN2B | Gprin3 |
| SLC30A7 | SLCO1A2 | Frmd3 |
| KCNMB4 | KIAA1211 | Zfp811 |
| POU3F3 | DLG2 | Igfn1 |
| POFUT1 | NKTR | Terf2 |
| VCAN | TET2 | Tnpo3 |
| ANKFY1 | LOC101928841 | Pgm2l1 |
| MDGA1 | DEPDC1B | Arhgef28 |
| DCAF17 | CEP135 | Tpm3 |
| TTC28 | FAM118A | Ogfrl1 |
| HSD17B8 | LMNB1 | Mycbp |
| FADS1 | NCAN | Slc7a2 |
| KCMF1 | KAT2A | Tmem45a2 |
| HDAC4 | PPP4R2 | Insr |
| IPMK | SUCO | Rinl |
| SFMBT1 | RAB33B | Ldlrad3 |
| IRS4 | PAQR9 | Pkn2 |
| ARID1B | TNFSF8 | Cth |
| PACS1 | RLIM | Prkag2 |
| CNOT1 | SLFN11 | Acta2 |
| TMEM120B | ZC3H13 | Atrnl1 |
| ERP44 | PTGDR | Plxnd1 |
| PIK3CA | EXD1 | Tbx22 |
| MAPRE2 | ZNF385C | Lrit2 |
| SBNO1 | GLRX | Dnajb4 |
| NIPBL | METTL8 | Edem3 |
| CADM1 | ARFGEF3 | Canx |
| DLK1 | TOPBP1 | Utp14a |
| TSPAN14 | SMCR8 | Gnpat |
| TAF3 | TMEM39A | Zfhx4 |
| CPNE2 | MSRB3 | Slc6a17 |
| RBM15B | LHX1 | Spata2 |
| UBE2K | ERI1 | 9530068E07Rik |
| CCDC6 | KBTBD8 | Pcnx |
| PFKM | CYP4F2 | Tnrc6a |
| ROBO1 | CEBPE | Pxdc1 |
| CACNG2 | IL16 | Bambi |
| SLC24A4 | RCAN3 | Gfod1 |

| | | |
|---|---|---|
| PAX6 | PTCD2 | Lrrc31 |
| TRIP4 | DIRAS2 | |
| CDC6 | SLC26A2 | |
| FCHSD1 | RIMS1 | |
| WISP1 | C16orf54 | |
| SMIM17 | CIART | |
| GABRB1 | HDHD2 | |
| DCC | PTGER4 | |
| EHF | NEK1 | |
| EIF2B3 | APAF1 | |
| GOLGA6B | STK3 | |
| GPR174 | EIF4EBP2 | |
| PTGDR | ZNF587B | |
| RGPD4 | CDS2 | |
| CISD2 | ADAM19 | |
| RP13-996F3.4 | GPR35 | |
| MAP3K2 | THRB | |
| TGFBR2 | MPP2 | |
| CACNA1A | MLYCD | |
| COL26A1 | TNPO3 | |
| DDB1 | MARK1 | |
| | EPB41L2 | |
| | UMAD1 | |
| | PTAFR | |
| | IFNA1 | |
| | TBP | |
| | TRIM50 | |
| | LETMD1 | |
| | PIGT | |
| | ZSCAN20 | |
| | CD93 | |
| | SP100 | |
| | XKRY2 | |
| | GVQW2 | |
| | CYB561D1 | |
| | TBCC | |
| | EFNB2 | |
| | HIVEP3 | |
| | RPGRIP1L | |
| | TTC39C | |
| | NCOA5 | |
| | HSPA1L | |
| | ARHGEF11 | |
| | XKRY | |

| |
|---|
| VEGFB |
| STAM |
| TNFAIP3 |
| DPYS |
| PAXBP1 |
| NRIP1 |

**Supplementary Table S3**: qRT-PCR and endpoint PCR Primer

| Gene | Direction | NCBI Reference Sequence* | Sequence (5'- 3') |
|---|---|---|---|
| ***β-Actin*** | for<br>rev | NM_007393.5 | CCAACCGTGAAAAGATGACC<br>ACCAGAGGCATACAGGGACA |
| ***Isl1*** | for<br>rev | NM_021459.4 | CCACGATGTGGTGGAGAGA<br>CTAGCCGAGATGGGTTCG |
| ***Nkx2.5*** | for<br>rev | NM_008700.2 | GAGCCTACGGTGACCCTGA<br>GTGGTCTCTCGGCGCCAT |
| ***Mef2c*** | for<br>rev | NM_025282.3 | ATGGGCGGAGATCTGACA<br>TTCTTGTTCAGGTTACCAGGTG |
| ***Acta2*** | for<br>rev | NM_007392.3 | CCAACCCGGGAGAAAATGAC<br>CAGTTGTACGTCCAGAGGCATA |
| ***Tbx5*** | for<br>rev | NM_011537.3 | GGATGTCTCGGATGCAAAGT<br>GGTTGGAGGTGACTTTGTGC |
| ***Tnnt2*** | for<br>rev | NM_011619.3 | TTCGACCTGCAGGAAAAAGTT<br>CTTCCCAGGAGTTTTGGAGA |
| ***Pecam1*** | for<br>rev | NM_008816.3 | GTTGCAGCCAAATGCTACTT<br>GAAATCTTCTCGCTGTTGGA |
| ***Myh6*** | for<br>rev | NM_010856.4 | CCTATGCTTCTGCTGATACCG<br>TCATCAGCTTGTTCAGATTTTCC |
| ***Cited2*** | for<br>rev | NM_010828.3 | GTTTAACAACTCCCAGTTCATGG<br>AATACTGGTTGTTGAGCTTCTGC |
| ***Hcn4*** | for<br>rev | NM_001081192.2 | CGCATTGTCCGTTTCACTAA<br>CAGGTCATAGGTCATGTGGAAG |
| ***Myl2*** | for<br>rev | NM_010861.4 | GACTGAGCCCTGAACCACAG<br>ACATCATCAACTTGCGGTCA |
| ***Shox2*** | for<br>rev | NM_013665.1 | CCCACTATCCAGACGCTTTC<br>TCGATTTTGAAACCAAACCTG |
| ***Myl7*** | for<br>rev | NM_022879.2 | CCCATCAACTTCACCGTCTT<br>AGGCACTCAGGATGGCTTC |
| ***Sfrp5*** | for<br>rev | NM_018780.3 | GATCTGTGCCCAGTGTGAGA<br>TTAATGCGCATCTTGACCAC |
| ***Ki67*** | for<br>rev | NM_001081117.2 | CCAGGAAAGTCCCTTGGAA<br>CATTTTTGAAGCTTTGGTATCTTG |
| ***Irx4*** | for<br>rev | NM_018885.2 | GATGAGAAGCGCCCCTATG<br>GTCTTTGCCAGCATGACCTT |

| | | | |
|---|---|---|---|
| ***Neurod1*** | for<br>rev | NM_010894.2 | **CGCAGAAGGCAAGGTGTC**<br>**TTTGGTCATGTTTCCACTTCC** |
| ***Nanog*** | for<br>rev | NM_028016.3 | **TTCTTGCTTACAAGGGTCTGC**<br>**AGAGGAAGGGCGAGGAGA** |
| ***Gata4*** | for<br>rev | NM_008092.3 | **GGAAGACACCCCAATCTCG**<br>**CATGGCCCCACAATTGAC** |
| ***Hand1*** | for<br>rev | NM_008213.2 | **CAAGCGGAAAAGGGAGTTG**<br>**GTGCGCCCTTTAATCCTCTT** |
| ***Nestin*** | for<br>rev | NM_016701.3 | **TGCAGGCCACTGAAAAGTT**<br>**TTCCAGGATCTGAGCGATCT** |
| ***Ncam1*** | for<br>rev | NM_001081445.1 | **ACCTGAAACCTGAGACGAGGT**<br>**CTTGGGTGCACTGGGTTC** |
| ***Afp*** | for<br>rev | NM_007423.4 | **GCTTATCTCTAAATCCAAGCCAGT**<br>**AGGAAGGTTGGGGTGAGTTC** |
| ***Sox17*** | for<br>rev | NM_011441.5 | **CAACGCAGAGCTAAGCAAGA**<br>**TTGTAGTTGGGGTGGTCCTG** |
| ***Sox2 (endogene)*** | for<br>rev | NM_011443 | **TCTGTGGTCAAGTCCGAGGC**<br>**TTCTCCAGTTCGCAGTCCAG** |
| ***cMyc (endogene)*** | for<br>rev | NM_001177352 | **TCAAGCAGACGAGCACAAGC**<br>**TACAGTCCCAAAGCCCCAGC** |
| ***Oct3/4 (endogene)*** | for<br>rev | NM_013633 | **CCAACGAGAAGAGTATGAGGC**<br>**CAAAATGATGAGTGACAGACAGG** |
| ***Klf4 (endogene)*** | for<br>rev | NM_010637 | **GGCGAGAACCTTACCACTGT**<br>**TACTGAACTCTCTCTCCTGGCA** |
| ***cMyc/Sox2 (exogene)*** | for<br>rev | NM_011443<br>NM_001177352 | **AGGGCGCCCTGCCAGGC**<br>**GGGAAGCAGCTCGAATTTCTT** |

*Data adopted from https://www.ncbi.nlm.nih.gov/nuccore/ (NCBI Nucleotide)

**Supplementary Table S4**: Antibodies

| Primary Antibody | Dilution | Secondary Antibody | Dilution |
|---|---|---|---|
| Anti-SOX2<br>(rabbit polyclonal IgG)***** | 1:250 | Goat anti rabbit IgG H&L<br>(Alexa Fluor 488)* | 1:500 |
| Anti-NANOG<br>(rabbit polyclonal IgG)***** | 1:100 | | |
| Anti-sarcomeric α-Actinin<br>(mouse monoclonal IgG1**)*** | 1:200 | | 1:500 |
| Anti-αMHC mouse (Myh6)<br>(monoclonal IgG2b)# | 1:100 | | |

| | | | |
|---|---|---|---|
| | | Goat anti mouse IgG H&L (Alexa Fluor 555)* | |
| Anti-αSMA (Acta2) (mouse monoclonal IgG)† | 1:20 | | |
| Anti-CD31 (CD31) (rabbit polyclonal IgG)* | 1:50 | Goat anti rabbit IgG H&L (Alexa Fluor 488)* | 1:500 |

Antibodies are purchased from *Abcam (Cambridge, UK), #R&D Systems (Minneapolis, MN, USA) or †Santa Cruz Biotechnology (Santa Cruz, CA, USA).

# 4. Supplementary Methods

### Isolation of cells for microRNA array analysis

GFP-positive CPCs were isolated from embryonic hearts at E9.5 of NkxCE-GFP mice from timed matings (a positive mating plug indicated E0.5). After animals were euthanized (as described above), embryos were collected and directly placed in ice-cold 1x PBS. Embryos were mechanically dissociated and digested with a Collagenase II (10.000 U/mL, Thermo Fisher Scientific, Waltham, ME, USA)/ DNase I (10.000 U/µL, Sigma Aldrich, St. Louis, MO, USA) solution for 1 h at 37°C. After washing with 1x PBS, cells were resuspended in FACS buffer (1x PBS / 0.5% BSA / 2 mM EDTA). GFP-positive cells and their negative counterparts were then sorted by flow cytometry and collected as described in the "flow cytometry" section (Suppl Fig S1A). In addition, *in vitro* differentiated GFP-positive CPCs were isolated by flow cytometry on day seven of the differentiation of NkxCE-GFP ES cells (ESCs) (Suppl Fig S1B). ESCs were differentiated by the "hanging drop" method, building embryoid bodies (EBs) as described in the respective "Differentiation of murine pluripotent stem cell lines" section. EBs were collected mechanically with a cell scraper on day seven and further digested as described for mouse embryos.

For isolation of tail tip (TTFs) and cardiac fibroblasts (CFs) (Suppl Fig S1C-D), NkxCE-GFP mice (age between six and eight weeks) were euthanized, the tailtip and the heart were removed and digested with the same Collagenase Typ II / DNaseI solution as mentioned above. Then, erythrocyte lysis was performed with red blood cell lysis solution (Miltenyi Biotec, Bergisch-Gladbach, Germany). Digested cells were centrifuged for 5 min at 300 xg and finally plated on gelatine-coated 6-well plates with fibroblast medium consistent of Dulbecco's MEM high glucose and stable glutamine (Biochrom, AG, Billierica, MA, USA), 10% FCS (Thermo Fisher Scientific, Waltham, MA, USA), 1% Penicillin/Streptomycin (Thermo Fisher Scientific, Waltham, MA, USA), 1% Sodium Pyruvate (Thermo Fisher Scientific, Waltham, MA, USA). After 24 hours, the cells were washed twice with 1x PBS and supplied with fresh medium. TTFs and CFs were further cultivated and directly harvested in RLTplus Buffer (Qiagen, Hilden, Germany) containing 10 µL/mL β-mercaptoethanol (Sigma-Aldrich, St. Louis, MO, USA) at low passage for RNA/miR isolation.

### Cell culture of murine pluripotent stem cells

Several murine pluripotent stem cell lines were utilized for this study such as non-transgenic V6.5 ESCs (Novus Biologicals, Centennial, CO, USA), transgenic NkxCE-GFP ESCs as well as self-generated ESCs/iPSCs including miR-128a-overexpressing NkxCE-

GFP ESCs (OE-128), the miR-Ctr-overexpressing NkxCE-GFP ESCs (OE-Ctr) (see below in "Generation of miR-overexpressing NkxCE-GFP ESC lines") and Isl1-Cre/ROSA$^{mTmG}$ iPSCs (iITG-iPSCs) as described in the "Generation of Isl1-Cre/ROSA$^{mTmG}$ iPS line" section. All above-mentioned murine ESCs/iPSCs were cultured on gelatinized 6-well plates coated with mitomycine-inactivated murine embryonic fibroblasts (MEFs) as feeder layer in ESC medium (Dulbecco's MEM high glucose, stable glutamine (Biochrom, AG, Billierica, MA, USA), 20% FCS (Thermo Fisher Scientific, Waltham, MA, USA), 1% Penicillin/Streptomycin (Thermo Fisher Scientific, Waltham, MA, USA), 1% non-essential amino acid solution (NEAA) (Thermo Fisher Scientific, Waltham, MA, USA), 0.1 mM β-mercaptoethanol (Sigma-Aldrich, St. Louis, MO, USA), and $10^3$ U/ml leukemia inhibitory factor (LIF) (Millipore, Billerica, MA, USA)). Cells were cultivated in a $CO_2$ incubator (5% $CO_2$) at 37°C. The ESC medium was changed every day and cells were passaged every second or third day at a ratio of 1:3 to 1:6. For the cell culture of miR-overexpressing NkxCE-GFP ESCs (OE-Ctr, OE-128), the ESC medium was supplied with tetracycline-free FCS (Biochrom, AG, Billierica, MA, USA) and 1µg/ml puromycin (GE Healthcare, Chicago, IL, USA) for a permanent selection pressure to keep established ESC clones. To test miR-induction, 2µg/ml doxycycline (Sigma-Aldrich, St. Louis, MO, USA) was added.

**Differentiation of murine pluripotent stem cells**

Murine ESCs and iPSCs were differentiated according to a standard "hanging drop" protocol as described previously[1-4]. In short, ESCs/iPSCs were grown on feeder free gelatin-coated 6-well plates for two days in IMDM-ES medium (Iscove's Modified Dulbecco's Medium (IMDM) with stable glutamine (Biochrom, AG, Billierica, MA, USA), 20% FCS (Thermo Fisher Scientific, Waltham, MA, USA), 1% Penicillin/Streptomycin (Thermo Fisher Scientific, Waltham, MA, USA), 0.1 mM 1-Thioglycerol (Sigma-Aldrich, St. Louis, MO, USA), and $10^3$ U/ml LIF (Millipore, Billerica, MA, USA)). IMDM-ES medium was supplied with tetracycline-free FCS (Biochrom, AG, Billierica, MA, USA) for iITG-iPSCs and miR-OE-ESCs.

To start differentiation, hanging drops (11 µl per droplet) were prepared on 15 cm cell culture dishes (V6.5 ESCs, NkxCE-GFP ESC and both OE-ESC lines 1,000 cells/droplet ($\sim 0.9*10^5$/ml); iITG-iPS 2000 cells/droplet ($\sim 1.8*10^5$/ml)) with differentiation medium (Iscove's Modified Dulbecco's Medium (IMDM) with stable glutamine (Biochrom, AG, Billierica, MA, USA), 20% heat-inactivated (30 min at 56°C) FCS (Thermo Fisher Scientific, Waltham, MA, USA), 1% Penicillin/Streptomycin (Thermo Fisher Scientific, Waltham, MA, USA), 0.1 mM 1-Thioglycerol (Sigma-Aldrich, St. Louis, MO, USA), 0.05mg/ml L-ascorbic acid (Sigma-Aldrich, St. Louis, MO, USA). The medium was supplied with tetracycline-free FCS (Biochrom, AG, Billierica, MA, USA) for experiments with iITG-iPSCs and miR-OE-ESCs.

The cell culture dishes were stored upside-down at 37°C (5% $CO_2$) for two days until embryoid bodies (EBs) had formed. The EBs were subsequently flooded with differentiation medium and either replated (see below) or differentiated further on 15 cm plates. Flooding of some miR-OE EBs was conducted with differentiation medium supplied with 2 µg/ml doxycycline (Sigma-Aldrich, St. Louis, MO, USA) for miR induction (Suppl Fig.10A).

For all miR knockdown and overexpression experiments, EBs of three to four 15 cm plates were pooled and replated on one gelatin-coated 12-well plate. For ICC stainings during differentiation, one EB was transferred into one well of a gelantin-coated 96 well plate. In general, differentiation medium was changed if necessary, either due to experimental set-up or when the medium was substantially consumed.

**Differentiation of hiPSCs**

HuiPSCs were differentiated according to the protocol of Burridge et al.[5] by modulating the Wnt-signaling pathway with several small molecules. Therefore, huiPSCs were grown as "monolayer" (single cells) until confluent in mTeSR E8 medium (Stem Cell Technologies, Cologne, Germany). The medium was changed at the beginning of differentiation to CDM3 medium (day 0), consisting of RPMI1640 medium (Sigma-Aldrich, St. Louis, MO, USA) supplemented with 500 µg/ml *O. sativa*–derived recombinant human albumin (Sigma-Aldrich, St. Louis, MO, USA) and 213 µg/ml L-ascorbic acid 2-phosphate (Sigma-Aldrich, St. Louis, MO, USA) which had to be freshly added each time the medium was replaced. Additionally, CDM3 was supplemented with 3 µM CHIR99021 (LC Laboratories, Woburn, MA) from day 0 to day 2. From day 2 to day 4, the cells received CDM3 supplemented with 2 µM WNT-C59 (Selleckchem, Munich Germany) (Suppl.Fig.S2B). Thereafter, the CDM3 medium without small molecules was replaced every other day. RNA was isolated every other day for analysis of microRNA kinetics during differentiation.

**RNA and miR isolation**

Isolation of miRs for microRNA array analysis from generated TTFs and CFs as well as FACS-isolated NkxCE-GFP+ CPCs and correspondent GFP-negative fractions was conducted with the miRVana microRNA isolation kit (Thermo Fisher Scientific, Waltham, MA, USA) according to the manufacturer's instructions (Fig. 1C&1E, Suppl.Fig S1A-D).

Total RNA, including small RNAs and microRNA of cultured murine ESCs/iPSCs as well as differentiated murine and human ESCs/iPSCs was isolated using the peqGold total RNA Kit according to manufacturer's instructions (Peqlab, Erlangen, Germany) (Fig. 1G, 3A, 4A, 5A , Suppl.Fig S2B, S7F, S8A, S9F,S10A).

Prior to RNA isolation of zebrafish embryos, embyos were collected at respective timepoints

(Fig. 2A&2C), covered with 400µl peqGold TriFast$^{TM}$ reagent and homogenized with syringes. RNA isolation was then conducted with the peqGold TriFast$^{TM}$ reagent (VWR International, Radnor, PA, USA) in compliance with the manufacturer's protocol.

If required for further experiments (e.g. miR or mRNA qRT-PCR), total RNA concentrations were determined by a Nanodrop 2,000 (VWR International, Radnor, PA, USA) and RNA was then stored at -20°C (short term) or -80°C (long term).

**MicroRNA array analysis**

MicroRNA array analysis was conducted with the above described islolated cells ("Isolation of Cells for MicroRNA Array Analysis") using a TaqmanTM Array Rodent MicroRNA A+B Card Set (Thermo Fisher Scientific, Waltham, MA, USA). Hereby, the expression of about 750 known miRs was evaluated and quantified by a Taqman 7500HT Fast Real-Time PCR System (Applied Biosystems, Foster City, CA, USA). For comparative analysis of miR expression profiles, U6 and sno202 were used as internal controls (ΔCt = Ct (Target) - Ct (Reference) and fold change (FC) was calculated by the $2^{-\Delta\Delta Ct}$ method[6]. miRs were considered differentially-expressed using a threshold of a |1.5| FC and two-sided Student's t-test p-value $\leq$ 0.05 between cell populations of interest (NkxCE-GFP CPCs *in vitro* and *in vivo* vs. respective GFP-negative fractions and NkxCE-GFP CPCs *in vitro* and *in vivo* CPCs vs. fibroblast populations).

**cDNA synthesis**

A total of 100 ng of extracted total RNA from all *in vitro* experiments (cells) was reverse transcribed into cDNA using M-MLV reverse transcriptase (Invitrogen, Darmstadt, Germany) and random hexamer primers (Thermo Fisher Scientific, Waltham, MA, USA) according to the manufacturer's recommendations in a Thermocycler C1000 (BioRad, Hercules, CA, USA). The following conditions were used: 5 min at 65°C, 2 min 37°C, 10 min 25°C, 50 min 37°C and 15 min 75°C.

For miR cDNA synthesis of all murine and human differentiated ESCs and iPSCs, 100 ng of total RNA were reverse transcribed using the Universal cDNA synthesis kit II (Exiqon, Vedbaek, Denmark) according to the manufacturer's recommendations in a Thermocycler C1000. The following conditions were used: 1h at 42°C, 5 min 95°C.

For reverse transcription of miRs isolated from zebrafish, the miScript II RT Kit (Qiagen, Hilden, Germany) was used under the following conditions: 1h at 37°C, 5 min 95°C.

**qPCR analysis**

Semiquantitative real time PCR (qRT-PCR) for evaluation of gene expression was performed with gene-specific primer sets (ELLA Biotech, Munich, Germany, Suppl Tab S3) using the Power SYBR Green PCR master mix (Applied Biosystems, Foster City, CA, USA) under the following conditions: Activation of *Taq* DNA polymerase (2 min at 50°C, 10 min at 95°C) followed by 40 cycles with 15 s at 95°C and 60 s at 60°C. Gene expression was calculated using arbitrary units (AU) and each sample was normalized to *β-actin.*

A real time PCR of miRs of *in vitro* generated cell samples was performed with miR specific primer sets (Exiqon, Vedbaek, Denmark; Suppl Tab S1) and ExiLENT SYBR Green Master mix (Exiqon, Vedbaek, Denmark) according to the miRCURY LNA$^{TM}$ Universal RT protocol under the following conditions: 10 min, 95°C, 10 min, 95°C (40 cycles), 60 min, 60°C. Amplification of miR from zebrafish samples was performed using the miScript Primer Assay (Qiagen, Hilden, Germany) and the miScript Green PCR Kit (Qiagen, Hilden, Germany) according to the manufacturer’s protocol. Each sample was normalized to U6 and internal control and quantification of miRs was calculated by the $2^{-\Delta\Delta C_T}$ method.

All real time PCRs were performed in MicroAmp Optical 96-well plates (Thermo Fisher Scientific, Waltham, MA, USA) on a QuantStudio™ 3 Real-Time PCR System (Thermo Fisher Scientific, Waltham, MA, USA) using the QuantStudio™ Design and Analysis Software v1.4.

**Morpholino microinjection**

Morpholinos (MOs) were synthesized by Gene Tool LLC, OR, USA (MO-miR-20b: TTGCAGTAGATCCACTGGCACTACC; MO-miR-30a: AGCTGAGATTTTCTGCATTACAACT; MO-miR-30b: AACACCCTCCGCCCAGCCTCGTC; MO-miR-128-1: ACTGTGAGAAAGCCTACATGAATCT). MOs were dissolved to a stock solution of 1 mM with Braun water and subsequently diluted with 200 mM KCl (Carl Roth, Karlsruhe, Germany) to the correspondent final concentrations. MOs were loaded into a fine-tipped needle and further injected into the yolk center of zebrafish embryos at the 1 or 2-cell stage (Fig 2C) by a microinjector. We injected 2-3 ng per embryo of MO-miR-20b; 1-2 ng per embryo of MO-miR-30a; 1-2 ng per embryo of MO-miR-30b, and 1-2 ng per embryo of MO-miR-128a. After MO microinjection, embryos were kept and grown in E3 medium as described in the “Transgenic Animals” section. After 24 hpf, the medium was replaced with E3 medium containing 0.003% PTU (1-phenyl-2-thiourea (Sigma-Aldrich, St. Louis, MO, USA) to reduce zebrafish skin pigmentation. Uninjected sibling embryos were processed as controls.

**Heart Rate and Fractional Shortening of Zebrafish Larvae**

Cardiac contraction videos at 72 hpf of MO knockdown and control larvae were recorded by a Leica DM IRB high-resolution video microscope (Leica, Wetzlar, Germany) with an optical

magnification of 20 and visually counted by at least two independent observers. To determine the diameter of ventricles during systole and diastole the Meazure™ v2.0 tool (C Thing Software, Mountain View, CA, USA) was used. The percentage of ventricular fractional shortening (VFS) was finally calculated by ((Diastole-Systole)/Diastole).

**Knockdown of miR-128a during *in vitro* differentiation by LNA probes**

Knockdown of miR-128 during *in vitro* differentiation of NkxCE-GFP ESCs and iITG-iPSCs was conducted using the miRCURY LNA™ Power microRNA inhibitor System (Exiqon, Vedbaek, Denmark). ESCs/iPSCs were either transfected with mmu-miR-128-3p probes (LNA-128; UCACAGUGAACCGGUCUCUUU, MIMAT0000140) or a control LNA probe (LNA-Ctr) as corresponding negative control (Exiqon, Vedbaek, Denmark) without any homology (>70%) to any sequence in any organism.

For transfection, ESCs/iPSCs were differentiated in 12-well plates using the hanging drop method as described above ("Differentiation of murine Pluripotent Stem Cell lines"). At specific time points during differentiation (Fig.3A& 4A), ESCs/iPSCs were transfected with LNA probes at a final concentration of 50 nM. In detail, medium was replaced by 500µl fresh differentiation medium without Pen/Strep. For transfection, 25 pmol of LNA probes were resuspended in pure IMDM (Biochrom, AG, Billierica, MA, USA) and supplied with 5µl of Fugene (Madison, WI, USA). After 15 min of incubation, the transfection mixture was added dropwise to the cells. After two days, the medium was changed to fresh differentiation medium with Pen/Strep and further analysis was performed (Fig.3A & 4A).

**Cell proliferation - MTT assay**

To evaluate influences of LNA probes, doxycycline addition or miR-128 overexpression on proliferation capacity of NkxCE-GFP ESCs or miR-OE ESCs MTT assay was used as previously described[2, 4].

NkxCE-GFP ESCs were cultured and differentiated as described above ("Differentiation of murine Pluripotent Stem Cell lines") and transfected with LNA-probes (LNA-Ctr) according to the respective protocol (Suppl.Fig4A). Differentiating cells were replated on gelatinized 48 well plates two days before the MTT assay was performed one week after LNA-transfection.

miR-OE ESCs were cultured as described above ("Cell Culture of murine Pluripotent Stem Cell lines") either with or without doxycyxline for three days on a gelatine-coated 96-well plate.

For MTT assays, ESCs were incubated with 10 µl of 3-[4.5-Dimethylthiazol-2-yl]-2.5-di phenyltetrazolium bromide (MTT) solution (conc. 5 mg/ml) (Sigma-Aldrich, St. Louis, MO,

USA) diluted in 90 µl DMEM basal medium (final conc. 0.5 mg/ml) for 1h at 37°C, 5% $CO_2$. For differentiating ESCs, MTT solution was diluted in IMDM basal medium. The MTT solution was then aspirated carefully and 100 µl of dimethyl sulfoxide (DMSO, Sigma-Aldrich, St. Louis, MO, USA) were added to each well to dissolve formazan. DMSO was thoroughly mixed in each well and then transferred to a fresh 96-well plate. The absorbance was measured at 570 nm on a Tecan Safire 2 Multimode Microplate Reader with the software XFLUOR4 version 4.51. The reference wavelength was set at 690 nm.

**Immunocytochemistry (ICC)**

Pluripotency of newly generated iITG-iPSCs and miR overexpressing ESCs (OE-Ctr, OE-128) was verified by immunostainings with anti-Sox2 and anti-Nanog antibodies (Suppl.Fig.S7G, Suppl.Fig. S9D, Suppl.Tabl.S3) as described previously[4]. ESCs and iPSCs were seeded on gelatine-coated 8-well chamber slides (BD, Franklin Lakes, NJ, USA) on a mitomycin-inactivated MEF feeder layer in ESC medium. Cells were fixed with ice-cold acetone (Sigma-Aldrich, St. Louis, MO, USA) for 10 min at -20°C and subsequently washed 3 times with 1x PBS. Permeabilization was conducted by incubation with 0.25% v/v Triton-X-100 (Carl Roth, Karlsruhe, Germany) diluted in 1x PBS (PBS-T) for 10 min. After another washing step with 0.1% v/v PBS-T, blocking was performed by incubation with 5% v/v normal goat serum (Santa Cruz, Dallas, TX, USA) in 1% v/v PBS-T (1x PBS-T) for 30 min at room temperature. In a first step, anti-Sox2 and anti-Nanog antibodies (both Abcam, Cambridge, UK) were diluted in 1.5% v/v goat serum in 1x PBS-T and incubated with the cells for 1 h at 37°C. After washing with 0.1% v/v PBS-T, an appropriate diluted Alexa-Fluor labeled secondary antibody (in 1.5% v/v goat serum in 1x PBS-T) (Suppl.Tabl.S3) was added to the cells at room temperature for 1 h in the dark. As a negative control, ESCs or iPSCs were stained with correspondent secondary antibodies only. Nuclear staining was conducted by covering the dried chamber slides in mounting medium containing 4′,6-Diamidin-2-phenylindol (DAPI) (Abcam, Cambridge, UK).

Cardiac linages including cardiomyocytes (CMs), smooth muscle cells (SMCs) and endothelial cells (ECs) were stained according to the above-mentioned protocol with antibodies for sarcomeric α-actinin, α-myosin heavy chain (α-MHC, MYH6), α-smooth muscle actin (α-SMA, ACTA2) and for platelet endothelial cell adhesion molecule (PECAM1, CD31). Cells were however fixed with 4% v/v paraformaldehyde in 1x PBS at room temperature, for these stainings. Correspondent antibodies as well as dilutions are listed in Suppl Tab S4.

**Flow cytometry**

For isolation of NkxCE-GFP-positive CPCs and their correspondent GFP-negative fractions for MicroRNA Array analysis (from E9.5 NkxCE-GFP embryos and day 7 differentiated

NkxCE-GFP ESCs), single cell suspensions were prepared as described in the respective “Isolation of Cells for MicroRNA Array Analysis” section (Fig. 1C, Suppl.Fig.1-A-B). Additionally, day seven differentiated NkxCE-GFP ESCs (independent samples for verification of MicroArray results) were harvested and single cell suspensions were prepared as described above (Fig. 1E).

Before this, flow cytometry cells were resuspended in FACS buffer (1x PBS / 0.5% w/v Bovine Serum Albumin (BSA, Thermo Fisher, Scientific, Waltham, MA, USA), 2 mM EDTA (Carl Roth, Karlsruhe, Germany)) and subsequently filtered through a 30 µm filter to prevent cell clumping. Dead cells were stained with propidium iodide (PI, final conc. 2 µg/ml) (Sigma-Aldrich, St. Louis, MO, USA) or DAPI (final conc. 1 µg/ml) (BioLegend, San Diego, CA, USA). Cell fractions were sorted directly into RLTplus Buffer (Qiagen, Hilden, Germany) containing 10 µl/ml β-mercaptoethanol (Sigma-Aldrich, St. Louis, MO, USA) for further RNA/miR isolation as described in the “RNA and miR isolation” section by a BD FACS ARIA III (BD Biosciences, San Jose, CA, USA) using the BD FACS Diva software version 6.1.2 (BD Biosciences).

For quantitative analysis of GFP-positive CPCs during *in vitro* differentiations (Fig.3D, Fig.4D, Suppl.Fig.S4D, Fig.5D, Suppl.Fig.S10C) or RFP-positive miR-OE-ESCs (Suppl.Fig.9E), flow cytometry was performed on a BD LSR Fortessa™ using the BD FACS Diva software version 6.2.

For quantification of GFP-positive CPCs during ESC/iPSC *in vitro* differentiations, EBs were scraped mechanically from cell culture dishes and enzymatically digested as described in the “Isolation of Cells for MicroArray Analysis” section, followed by resuspension in FACS buffer. For quantification of RFP-positive miR-OE-ESCs (cultured with 2µg/ml or without doxycycline), ESCs were detached with 0.25% Gibco™Trypsin-EDTA solution (Thermo Fisher Scientific, Waltham, MA, USA), and subsequently resuspended in FACS buffer. All samples were filtered through a 30 µm filter before flow cytometry and dead cells were stained with DAPI (final conc. 1 µg/ml).

The final analysis of results was conducted with the FlowJo 7.6.5 software (FlowJo, LLC, Ashland, OR).

**Analysis of beating frequency of cell clusters during *in vitro* differentiation**

Beating frequency (or beating rate) of differentiating miR-128 knockdown and overexpression ESCs/iPSCs and corresponding controls were evaluated using the Nikon Eclipse Ts2 inverted microscope (Software TiControl Ver. 4.4.2.) video feature at respective timepoints (Fig 3J& 4F& 5G). Nine videos of different beating foci per condition per time point with a

length of 20 sec were recorded and beats of the beating areas were subsequently counted visually by at least two independent observers. Beating rates were calculated by dividing the number of counts by time (beats/min, bpm).

**RNA sequencing (RNAseq) analysis**

RNAseq data from a previous study were reanalyzed[7]. Original sequencing data were deposited at the Sequencing Read Archive (SRA, PRJNA229481). For this study, NkxCE-GFP-positive CPCs from embryonic hearts at E9 and E11 of NkxCE-GFP mice were obtained by enzymatic digestion as described in detail in the “Cell isolation for MicroRNA Array analysis” section. For embryonic stages > E10, erythrocyte lysis (red blood cell lysis solution, Miltenyi Biotec, Bergisch-Gladbach, Germany) was additionally performed. Single cell suspensions were resuspended in FACS buffer, filtered (30 µm) and dead cells were stained with PI (final conc. 2 µg/ml) before GFP-positive CPCs were subsequently sorted directly into RLTplus buffer (Qiagen) containing 10 µl/ml β-mercaptoethanol (Sigma-Aldrich, St. Louis, MO, USA) by a BD FACS ARIA III (BD Biosciences, San Jose,CA, USA) as described in the “Flow Cytometry” section. DNA and total RNA were then extracted using the AllPrep Kit (Qiagen, Hilden, Germany) in accordance with the manufacturer´s instructions. In addition, cardiomyocytes (CMs) were isolated from C57/Bl6 mice on postnatal day 21 (P21). In brief, hearts were retrogradially perfused with digestion buffer for 12 min until the enzymatic digestion was stopped by the addition of 5% FCS followed by a filtration step (100µm filter). CMs were identified by a high FSC signal and viable cells were discriminated by Draq5 (Cell Signaling Technology, Frankfurt, Germany).

For RNAseq, polyadenylated RNA was isolated from total RNA using magnetic beads (NEBNext Poly(A) mRNA Magnetic Isolation Module, NEB, Frankfurt, Germany). Libraries were constructed using the NEBNext Ultra RNA Library Prep Kit for Illumina (NEB) according to the manufacturer´s instructions and corresponding relative mRNA levels were calculated.

**Lentiviral production**

Lentivirus production was previously described by Doppler et al.[2]. In short, muSTEMCCA and rtTA lentiviruses were produced by 293FT cells (Thermo Fisher Scientific, Waltham, MA, USA). $7x10^6$ 293FT cells on gelatine coated 10 cm dishes were transfected after 24h either with the pHAGE-STEMCCA (muSTEMCCA) expression vector[8] or the FUdeltaGW-rtTA plasmid (referred to as rtTA)[9], each combined with the CMV-VSV-G plasmid[10] for viral envelope protein expression as well as the pCMV-dR8.2 dvpr for viral packaging[10]. Transfections were performed in 10 ml of Pen/Strep-free fibroblast medium with the FuGENE™ transfection reagent (Promega, Madison, WI, USA). FuGENE-DNA complexes were prepared containing 11 µg of the respective lentiviral plasmid DNA (either pHAGE-

STEMCCA or FUdeltaGW-rtTA), 5.5 µg of pCMV-VSV-G as well as 8.25 µg of pCMV-R8.2 diluted in 600 µl basal DMEM (Biochrom, AG, Billierica, MA, USA) supplied with 50 µl (ratio 4:2, 50 µl to 25 µg total DNA) of the transfection reagent. After 15 min of incubation at room temperature, FuGENE-DNA complexes were added drop-wise to the 293FT cells.

Virus-containing supernatants of 293FT cells were collected after 72 h, centrifuged for 10 min at 300 x g, filtered (0.45 µm) and then directly applied to ITG fibroblasts for reprogramming as described in detail in the "Generation if Isl1-Cre/ROSA$^{mTmG}$ iPS line' section below.

**Generation of Isl1-Cre/ROSA$^{mT/mG}$ iPSCs (iITG-iPSCs)**

Isl1-Cre/ROSA26$^{mT/mG}$ is a double-fluorescent Cre reporter mouse line, which contains membrane-tagged tandem dimer Tomato (mT) and membrane-tagged green fluorescent protein (mG) (Suppl.Fig.S7A), which was kindly provided by Prof. Sylvia Evans (University of California, San Diego). Upon Cre-mediated excision of mT, *Isl1*-positive CPCs will be marked by GFP from E8.5 of embryonic development[11, 12]. Once Isl1-positive CPCs are marked, their progeny stays GFP-positive. For reprogramming into iPSCs (further referred to as iITG-iPSCs), primary tailtip fibroblasts (TTFs) were used at a low passage (p 3) from an Isl1-Cre/ROSA26$^{mT/mG}$ mouse. Reprogramming was achieved by co-transducing fibroblasts with doxycycline inducible muSTEMCCA expressing mOct-4, mKlf4, mSox-2, and mc-Myc[8] ((Suppl.Fig.S7B) and rtTA[9] lentiviral particles, which were generated as described above in the "lentiviral production" section. For transduction, $2*10^5$ TTFs were resuspended in 500 µl fibroblast medium without Pen/Strep, supplied with polybrene (Sigma-Aldrich, St. Louis, MO, USA) at a final concentration of 8 µg/ml and mixed with 1,000 µl of each lentiviral supernatant from 293FT cells (muSTEMCCA and rtTA) ("lentiviral production"). Transduced fibroblasts were then seeded on gelatin-coated 6-well plates. After 24 h, the cells were washed twice and supplied with fresh fibroblast medium (with Pen/Strep) containing 2 µg/ml doxycycline for viral induction. After reaching 80-90% confluency, the cells were split at a 1:3 ratio to a mitomycin-inactivated MEF feederlayer with ESC medium supplied with 2 µg/ml doxycycline and the double amount of LIF ($10^6$ U/ml). The medium was changed every other day until iPSC clones first started evolving (Suppl. Fig. S7C, D (left pane)). After two to three weeks, selected clones were picked using a 20 µl pipette tip and transferred into 50 µl 0.25% Gibco™Trypsin-EDTA solution (Thermo Fisher Scientific, Waltham, MA, USA) for dissociation (5 min at 37°C). After centrifugation (5 min at 300 x g), each clone was resuspended in 500 µl ESC medium supplied with 1 µg/ml doxycycline and the double amount of LIF ($10^6$ U/ml) and seeded into one well of a 24 well plate on an inactivated MEF feeder layer. The ESC medium was changed every day and iPSC clones were further cultivated as described in the "Cell culture of murine pluripotent stem cell lines" section. Before being used for knockdown experiments (Fig. 4A), generated iITG-iPSC clones were

verified regarding their pluripotency (Suppl.Fig.S7E-G) as well as for suitable differentiation capacity into all three germ layers (Suppl.Fig.S8).

**Generation of miR-overexpressing NkxCE-GFP ESC lines**

The NkxCE-GFP miR-128a-overexpressing ESCs (OE-128) as well as the miR-Ctr-overexpressing (OE-Ctr) ESCs were generated by transduction with either shMIMIC mouse doxycycline-inducible microRNA mmu-miR-128-3p mCMV-TurboRFP (shMIM-128) or SMARTvector non-targeting Control mCMV/TurboRFP lentiviruses (shMIM-Ctr) (Dharmacon, Lafayette, CO, USA) (Suppl.Fig.S9A).

Initially, transgenic NkxCE-GFP ESCs were cultured as described in the respective "Cell Culture of murine Pluripotent Stem Cell lines" section. For transduction, $2*10^5$ ESCs (p16) were resuspended in 50 µl transduction medium (ESC medium without FCS and Pen/Strep) and finally mixed with $3.32*10^7$ transfection units (TU) of shMIM-128 or $2.9*10^6$ TU of shMIM-Ctr lentiviruses. Polybrene (Sigma-Aldrich, St. Louis, MO, USA) was added at a final concentration of 8 µg/ml and transduced NkxCE-GFP ESCs were seeded on one well of a 48-well plate on an inactivated MEF feeder layer and incubated for 24h. The transduction medium was subsequently aspirated and changed into ESC medium containing teteracycline-free FCS (Biochrom, AG, Billierica, MA, USA) and 2 µg/ml doxycycline to induce lentiviral constructs. Efficiently transduced ESCs, also indicated by RFP-fluorescence, were additionally selected by adding 1 µg/ml puromycin to the above-mentioned medium after three days of culture. The medium was changed every day until day seven when each well of the 48-well plate was transferred to one well of a 6-well plate on inactivated MEF feeder layers. After two weeks, RFP-positive clones from generated miR overexpression ESCs (OE-Ctr, OE-128) were picked (Suppl.FigS9B-C) as described in detail above in the "Generation if Isl1-Cre/ROSA$^{mTmG}$ iPS line' section. All picked miR-OE-ESC clones were further cultivated/expanded in tetracycline-free ESC medium supplied with 1µg/ml puromycin (in addition to doxycycline, if required for induction). After verification of pluripotency (Suppl.FigS9D), RFP- and miR-128a expression (Suppl.Fig.S9E-F), proliferation capacity (Suppl.FigS9G) as well as differentiation behavior (Suppl.Fig.S10) selected miR-OE-ESC clones were used for further *in vitro* differentiation experiments as described in (Fig.5A).

**Microscopic Imaging**

All phase contrast and fluorescent images of fibroblasts, ESCs/iPSCs and immunostainings were taken with an AxioCam MR camera on an Axiovert 200M microscope (Zeiss, Oberkochen, Germany) equipped with 10x and 40x objectives, combined with a 10x ocular resulting in 100x to 400x magnification, supported by the AxioVison Rel 4.8 software (Zeiss).

Images of zebrafish embryos (24 hpf 6.3x magnification; 48 hpf or 72 hpf 2.5 or 3.2x magnifications) were taken by using the microscope Olympus SZX16 and Olympus XC30 camera (Olympus, Hamburg, Germany) supported by the CeliSens Entry software (Olymous).

**Data Analysis and Statistical Analysis**

All graphs were processed using GraphPad Prism Software Version 7.0d. Descriptive data analysis was performed using IBM SPSS Statistics (Version 22, SPSS Inc, Chicago, USA). Comparisons between two groups were made with the two-tailed t-test or Mann-Whitney Rank Sum test. Data are shown as mean values ± standard error of the mean (S.E.M). Values of $p \leq 0.05$ were considered to be statistically significant. The statistic in all figures is indicated by * $p \leq 0.05$, ** $p \leq 0.01$ and *** $p \leq 0.001$.

**Data Availability**

The authors confirm that all data underlying the presented findings are fully available without restriction upon reasonable request to the authors. Original RNA sequencing data[7] were deposited at the Sequencing Read Archive (SRA, PRJNA229481). All relevant data are in the paper and its supporting information files.

# 6. Supplementary References


1. Deutsch, M. A.; Doppler, S. A.; Li, X.; Lahm, H.; Santamaria, G.; Cuda, G.; Eichhorn, S.; Ratschiller, T.; Dzilic, E.; Dressen, M.; Eckart, A.; Stark, K.; Massberg, S.; Bartels, A.; Rischpler, C.; Gilsbach, R.; Hein, L.; Fleischmann, B. K.; Wu, S. M.; Lange, R.; Krane, M., Reactivation of the Nkx2.5 cardiac enhancer after myocardial infarction does not presage myogenesis. *Cardiovasc Res* **2018,** 114, (8), 1098-1114.
2. Doppler, S. A.; Werner, A.; Barz, M.; Lahm, H.; Deutsch, M. A.; Dressen, M.; Schiemann, M.; Voss, B.; Gregoire, S.; Kuppusamy, R.; Wu, S. M.; Lange, R.; Krane, M., Myeloid zinc finger 1 (Mzf1) differentially modulates murine cardiogenesis by interacting with an Nkx2.5 cardiac enhancer. *PLoS One* **2014,** 9, (12), e113775.
3. Huang, X.; Wu, S. M., Isolation and functional characterization of pluripotent stem cell-derived cardiac progenitor cells. *Curr Protoc Stem Cell Biol* **2010,** Chapter 1, Unit 1F 10.
4. Lahm, H.; Doppler, S.; Dressen, M.; Werner, A.; Adamczyk, K.; Schrambke, D.; Brade, T.; Laugwitz, K. L.; Deutsch, M. A.; Schiemann, M.; Lange, R.; Moretti, A.; Krane, M., Live fluorescent RNA-based detection of pluripotency gene expression in embryonic and induced pluripotent stem cells of different species. *Stem cells* **2015,** 33, (2), 392-402.
5. Burridge, P. W.; Matsa, E.; Shukla, P.; Lin, Z. C.; Churko, J. M.; Ebert, A. D.; Lan, F.; Diecke, S.; Huber, B.; Mordwinkin, N. M.; Plews, J. R.; Abilez, O. J.; Cui, B.; Gold, J. D.; Wu, J. C., Chemically defined generation of human cardiomyocytes. *Nat Methods* **2014,** 11, (8), 855-60.
6. Schmittgen, T. D.; Livak, K. J., Analyzing real-time PCR data by the comparative C(T) method. *Nat Protoc* **2008,** 3, (6), 1101-8.

7. Nothjunge, S.; Nuhrenberg, T. G.; Gruning, B. A.; Doppler, S. A.; Preissl, S.; Schwaderer, M.; Rommel, C.; Krane, M.; Hein, L.; Gilsbach, R., DNA methylation signatures follow preformed chromatin compartments in cardiac myocytes. *Nat Commun* **2017,** 8, (1), 1667.
8. Sommer, C. A.; Stadtfeld, M.; Murphy, G. J.; Hochedlinger, K.; Kotton, D. N.; Mostoslavsky, G., Induced pluripotent stem cell generation using a single lentiviral stem cell cassette. *Stem Cells* **2009,** 27, (3), 543-9.
9. Maherali, N.; Ahfeldt, T.; Rigamonti, A.; Utikal, J.; Cowan, C.; Hochedlinger, K., A high-efficiency system for the generation and study of human induced pluripotent stem cells. *Cell Stem Cell* **2008,** 3, (3), 340-5.
10. Stewart, S. A.; Dykxhoorn, D. M.; Palliser, D.; Mizuno, H.; Yu, E. Y.; An, D. S.; Sabatini, D. M.; Chen, I. S.; Hahn, W. C.; Sharp, P. A.; Weinberg, R. A.; Novina, C. D., Lentivirus-delivered stable gene silencing by RNAi in primary cells. *RNA* **2003,** 9, (4), 493-501.
11. Muzumdar, M. D.; Tasic, B.; Miyamichi, K.; Li, L.; Luo, L., A global double-fluorescent Cre reporter mouse. *Genesis* **2007,** 45, (9), 593-605.
12. Yang, L.; Cai, C. L.; Lin, L.; Qyang, Y.; Chung, C.; Monteiro, R. M.; Mummery, C. L.; Fishman, G. I.; Cogen, A.; Evans, S., Isl1Cre reveals a common Bmp pathway in heart and limb development. *Development* **2006,** 133, (8), 1575-85.
